# A novel peptide 66CTG stabilizes Myc proto-oncogene protein to promote triple-negative breast cancer growth

**DOI:** 10.1038/s41392-025-02298-5

**Published:** 2025-07-09

**Authors:** Huichun Liang, Fubing Li, Huan Fang, Wenlong Ren, Zhongmei Zhou, Jiecheng Wang, Jialing Liu, Yongjia Tang, Xue Liu, Yingying Wu, Jing Peng, Chuanyu Yang, Jiayi Chen, Yuting Fei, Yujie Shi, Dewei Jiang, Nu Zhang, Ceshi Chen

**Affiliations:** 1 Key Laboratory of Animal Models and Human Disease Mechanisms of the Chinese Academy of Sciences and Yunnan Province, Kunming Institute of Zoology, Chinese Academy of Sciences, Kunming, China; 2 Yunnan Key Laboratory of Breast Cancer Precision Medicine, Academy of Biomedical Engineering, Kunming Medical University, Kunming, China; 3 The School of Continuing Education, Kunming Medical University, Kunming, China; 4 Yunnan Key Laboratory of Breast Cancer Precision Medicine, The Third Affiliated Hospital, Kunming Medical University, Kunming, China; 5 Hangzhou Institute of Medicine, Chinese Academy of Sciences, Hangzhou, China; 6 Center of Medical Laboratory, General Hospital of Ningxia Medical University, Yinchuan, China; 7 Department of Pathology, The First Affiliated Hospital, Kunming Medical University, Kunming, China; 8 Department of Pathology, Henan Provincial People’s Hospital, Zhengzhou University, Zhengzhou, China; 9 Department of Neurosurgery, First Affiliated Hospital of Sun Yat-sen University, Guangdong Provincial Key Laboratory of Brain Function and Disease, Guangdong Translational Medicine Innovation Platform, Guangzhou, China

**Keywords:** Breast cancer, Non-coding RNAs, Breast cancer

## Abstract

Triple-negative breast cancer (TNBC) is the most malignant subtype of breast cancer that lacks reliable targets for diagnosis and therapy. Non-coding RNA (ncRNA)-encoded products hold promise for addressing this unmet need. By analyzing the reported ribosomal RNA sequencing data, combined with the TCGA, ORFfinder, SmProt databases, we identified CDKN2B-AS1, a TNBC-upregulated lncRNA encoding a 66-amino-acid peptide via CUG-initiated translation. CRISPR-Cas9 gene editing and mass spectrometry confirmed endogenous expression of this peptide, designated 66CTG, in TNBC cells. Functionally independently of its host RNA, 66CTG promoted the proliferation of TNBC cells and the tumor growth of TNBC xenograft by stabilizing c-Myc protein and enhancing Cyclin D1 transcription. Immunohistochemistry of 89 clinical TNBC paraffin samples revealed positive correlations among 66CTG, c-Myc, and Cyclin D1 expression levels. Mechanistically, co-immunoprecipitation and ubiquitination assays revealed that 66CTG stabilized c-Myc by competitively interacting with FBW7α, an E3 ligase responsible for recognizing 66CTG CPD^S56/S60^ motif which phosphorylated by GSK-3β during the late G1 phase. In conclusion, our findings suggest 66CTG has potential to be developed as a target for TNBC diagnosis and therapy. Furthermore, it unveils a regulatory axis wherein 66CTG stabilizes c-Myc by interacting with FBW7α, offering a new mechanistic explanation for c-Myc overexpression in TNBC. Patients co-overexpressing 66CTG, c-Myc, and Cyclin D1 may benefit from therapies targeting this axis.

## Introduction

Triple-negative breast cancer (TNBC) represents a distinct subtype of breast cancer which is defined by the absence of estrogen receptor (ER), progesterone receptor (PR), and human epidermal growth factor receptor 2 (HER2) expression.^1–3^ Although comprising only 12–17% of total breast cancers, this subtype is the most aggressive, characterized by rapid growth, poor differentiation, drug resistance, high recurrence rates, and significant metastasis capability.^4^ Due to a lack of specific targets for therapy, the clinical therapeutic approaches for TNBC mainly include surgery, radiation and chemotherapy.^5^ For a portion of TNBC patients harboring BRCA1/2 germline mutations or with PDL1-positive expression, chemotherapy combined with poly (ADP-ribose) polymerase (PARP) inhibitors or immune checkpoint inhibitors is used in clinical treatment. However, this combination therapy has neither significantly improved the therapeutic efficacy for TNBC nor notably reduced the toxic side effects.^6^ Therefore, exploring the molecular pathways driving the development of TNBC and identifying specific therapeutic targets remains an urgent and challenging task in TNBC research.

Increasing evidence reveals that non-coding RNAs (ncRNAs), which include circular RNAs (circRNAs) and long non-coding RNAs (lncRNAs), are capable of encoding functional polypeptides.^7,8^ The proteins encoded by ncRNAs contribute significantly to the regulation of both physiological and pathological states.^9–11^ In TNBC, some micropeptides encoded by ncRNAs have been identified as being involved in some critical oncogenic processes, including tumor growth, metastatic spread, and the development of therapeutic resistance.^12–14^ These suggest that the coding function of ncRNAs offers a valuable resource for discovering TNBC-specific therapeutic targets. Additionally, canonical ORFs typically begin with the AUG start codon. However, many coding products in eukaryotic cells use non-AUG codons (such as CUG, UUG, GUG, ACG, AUA, and AUU) for translation initiation.^7,15–17^ These products encoded by non-AUG start codons are worthy of our attention. A prominent illustration of this phenomenon is the oncoprotein c-Myc. Its synthesis commences from a CUG initiation codon located within the transcript of the *MYC* oncogene,^18^ and it fulfills essential functions in both the initiation and progression of diverse cancer types.

Amplification of the *MYC* gene is observed in 60% of TNBC cases, which is higher than in other subtypes of breast cancer and indicates a poor prognosis.^19^ Its encoded protein, c-Myc, significantly promotes TNBC cells to proliferate, migrate, invade, and resist to drug. Therefore, targeting c-Myc is a viable strategy for TNBC treatment.^20,21^ Several small-molecule inhibitors targeting c-Myc, such as C1572 and MYCi975, have been developed and shown efficacy in pre-clinical or clinical tails of TNBC.^22,23^ However, long-term use of these inhibitors results in systemic toxicity due to the inhibition of c-Myc physiological functions.^24^ Thus, further investigation into the specific mechanisms by which c-Myc promotes TNBC is necessary. In cell proliferation, c-Myc is tightly regulated by mitotic signals^25^ and promotes cell to cross the R checkpoint in the late G1 phase by enhancing the transcription of Cyclin D1/D2 and suppressing the transcription of p15, p16, and p21.^26,27^ At this stage, with the shrinking of mitotic signals, the T58 and T244 sites of c-Myc are phosphorylated by GSK-3β (glycogen synthase kinase 3β), which are then specifically recognized by FBW7 (F-box and WD repeat domain containing 7) and recruited to the SCF (Skp1-Cullin-F-box-protein) complex for ubiquitination and degradation.^28–30^ In several types of cancer, due to the deletion, mutation and silencing of *FBW7* gene, c-Myc is highly expressed.^31,32^ However, in clinical breast cancer^32^ and breast cancer cell lines,^33^ the frequency of *FBW7* gene mutation is very low. Although one study showed that the probability of *FBW7* gene silencing by promotor methylation is approximately 50% in breast cancer, it only included three cell lines of breast cancer and four samples of primary breast cancer.^34^ Therefore, the expression level of FBW7 in TNBC and the mechanisms underlying c-Myc overexpression in TNBC remain unclear and warrant further investigation.

In this study, to identify specific biomarkers and therapeutic targets for TNBC, we focused on ncRNA-encoded products by removing the restriction that the start codon must be AUG. Consequently, we discovered a noveland unannotated small peptide, named 66CTG, which is encoded by lncRNA CDKN2B-AS1 overexpressed in TNBC using a CUG start codon. This peptide enhanced the transcription of Cyclin D1 by stabilizing c-Myc, thereby promoting cell proliferation and the tumor growth of TNBC. In 89 samples of clinical TNBC, 66CTG was highly expressed, and the expression level of this peptide was positively correlated with that of c-Myc and Cyclin D1. We further demonstrated that 66CTG stabilized c-Myc through competitive interaction with FBW7α during the late G1 phase, while FBW7α mediated the ubiquitination and degradation of 66CTG by recognizing its CPD^S56/S60^ motif. Our work not only identified a new and potential biomarker and target for TNBC diagnosis and therapy, but also elucidated the mechanisms of c-Myc overexpression in TNBC. This may benefit the treatment of TNBC, especially for those with high expression levels of 66CTG, c-Myc, and Cyclin D1.

## Results

### A novel peptide 66CTG encoded by CDKN2B-AS1 promotes TNBC cell proliferation

**Fig. 1:**
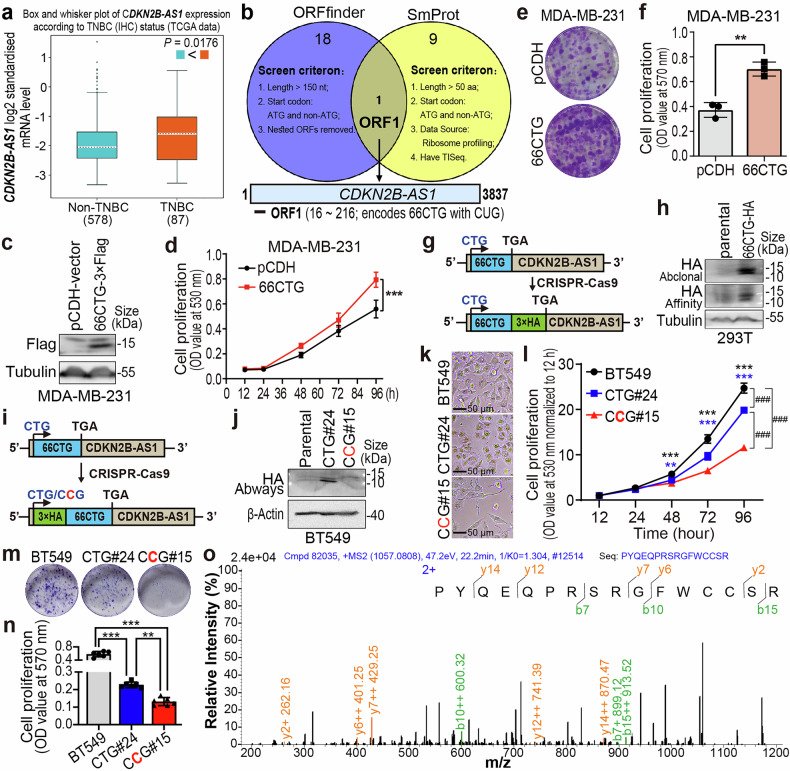
66CTG encoded by CDKN2B-AS1 promotes TNBC cell proliferation. **a** CDKN2B-AS1 expression levels in non-TNBC (*n* = 578) and TNBC (*n* = 87) clinical samples from the TCGA database were analyzed using bc-GenExMiner v5.0. **b** Illustration of the screen process of ORF1 obtained from ORFfinder and SmProt, as well as the localization of ORF1 on CDKN2B-AS1. **c** Overexpression of 66CTG-3×Flag was detected in MDA-MB-231 cells via Western blotting. **d** SRB assays assessed the effect of 66CTG-3×Flag overexpression on the proliferation of MDA-MB-231 cells (*n* = 10). Error bars show the mean ± SD, ***P < 0.001 by two-way ANOVA. **e** The images of the colony formation of MDA-MB-231 cells with 66CTG-3×Flag overexpression. **f** Statistical results of colony formation assay of (**e**) (*n* = 3). Error bars show the mean ± SD, **P < 0.01 by two-tailed Student’s *t*-test. **g** Schematic representation of the insertion of a 3xHA tag sequence at the C-terminus of the 66CTG gene via CRISPR-Cas9-mediated homologous recombination. **h** WB was employed to detect HA expression, which was knocked in at the C-terminus of 66CTG in the HEK293T clonal cell line using CRISPR-Cas9. The anti-HA-tag antibodies used in this experiment included anti-HA-tag (Abclonal, Cat#AE008), anti-HA-tag (Affinity Biosciences, Cat#T0008). **i** Schematic representation of the insertion of a 3xHA tag sequence at the N-terminus of the 66CTG gene via homologous recombination using CRISPR-Cas9, along with the mutation of the start codon from CTG to CCG. **j** Western blotting was employed to detect the expression of HA, which was inserted at the N-terminal of 66CTG, along with the mutation from CTG to CCG via CRISPR-Cas9 in BT549 clonal cell lines. The anti-HA-tag antibody used in this experiment was obtained from Abways (Cat#AB0004). **k** Cell morphology images were obtained for BT549 parental and the two clonal cell lines: BT549-N-3×HA-66CTG#24 and BT549-N-3×HA-66CCG#15. **l** The SRB assay assessed the proliferation of BT549-parental, BT549-N-3×HA-66CTG#24 and BT549-N-3×HA-66CCG#15 (*n* = 12). Error bars show the mean ± SD, **P < 0.01, ***P < 0.001 compared to BT549-N-3×HA-66CCG#15, and ^###^*P* < 0.001 compared to each other by two-way ANOVA. **m** The images of the colony formation of BT549-parental, BT549-N-3×HA-66CTG#24 and BT549-N-3×HA-66CCG#15. **n** Statistical results of colony formation assay of (**m**) (*n* = 6). Error bars show the mean ± SD, **P < 0.01, ***P < 0.001 by two-tailed Student’s *t*-test. **o** Mass spectrometry analysis of endogenous 66CTG expression in BT549 cells

It is reported that LncRNA-encoded proteins have a crucial role in the progression of many kinds of cancer.^35^ In order to find specific diagnostic and therapeutic targets for TNBC, we focused on the coding functions of ncRNAs. According to a report on ribosome sequencing of breast cancer cells, CDKN2B-AS1 is one of the lncRNAs with coding potential.^36^ This lncRNA was significantly overexpressed in clinical breast cancer tissues compared with adjacent normal tissue (Supplementary Fig. 1a–c). Notably, in TNBC, this lncRNA showed significant upregulation (Fig. 1a and Supplementary Fig. 1c, d) by using bc-GenExMiner v5.2^37^ and BCIP,^38^ and was associated with poor prognosis (Supplementary Fig. 1e) by using cBioPortal.^39^ In order to study the coding function of CDKN2B-AS1, we utilized ORFfinder^40^ to predict potential ORFs of CDKN2B-AS1 (NR_003529.4) with a specific screening criterion (including: nucleotide length more than 150 nt, exclusion of ATG as the start codon, and non-inclusion of nested ORFs), and identified 19 ORFs in total (Supplementary Table 3). Using SmProt,^41^ we screened for unannotated coding products of CDKN2B-AS1 (NC_000009.12) that were confirmed by ribosome sequencing. The specific criterion included excluding ATG as the start codon, using data from ribosome RNA sequencing, requiring an amino acid length of more than 50 aa, and the presence of translation initiation sequencing (TISeq) data. As a result, we identified 9 unannotated small peptides translated from CDKN2B-AS1 (Supplementary Table 4). We further conducted overlapping alignment analysis on the results obtained from ORFfinder and SmProt, ultimately identifying ORF1, which translates a 66-amino-acid peptide with CUG as the start codon (Fig. 1b). Then, we cloned ORF1 with a C-terminal 3×Flag tag fusion into the pCDH plasmid and confirmed that ORF1 indeed utilizes CUG to begin encoding a small peptide (Supplementary Fig. 1f). We named this peptide 66CTG.

In order to explore the function of 66CTG in TNBC, 66CTG-3×Flag was overexpressed in two cell lines of TNBC, MDA-MB-231 and HCC1806 (Fig. 1c and Supplementary Fig. 1k), as the expression levels of the 66CTG-encoding gene *CDKN2B-AS1* are relatively low in these two cell lines (Supplementary Fig. 1g). Results from SRB (Fig. 1d and Supplementary Fig. 1l) and colony formation (Fig. 1e, f and Supplementary Fig. 1m, n) assays demonstrated that 66CTG promoted the proliferation of TNBC cells. Next, to determine if endogenous 66CTG peptide is expressed in cells, we inserted a 3×HA tag sequence at the C-terminus of ORF1 in the HEK293T cell line through homologous recombination by using CRISPR-Cas9 (Fig. 1g). Through the isolation, screening, and sequencing of clones, we successfully generated a clonal cell line with a 3×HA tag inserted at the C-terminus of ORF1. The expression level of HA tag was verified by using Western blot, which confirmed the presence of endogenous 66CTG peptide in the cells (Fig. 1h). The expression of endogenous 66CTG in BT549 cells was also confirmed by mass spectrometry analysis (Fig. 1o). Because the knockdown of CDKN2B-AS1 in three cell lines of TNBC, including BT549, MDA-MB-468, and Hs578T (Supplementary Fig. 1h, i), failing to show a significant and consistent impact on cell proliferation (Supplementary Fig. 1j), we speculate that the ability of 66CTG to promote the proliferation of cancer cells is independent of CDKN2B-AS1. To assess the biological activity of the endogenous 66CTG, we inserted a 3xHA tag sequence at the N-terminus of ORF1 in BT549 cells by using CRISPR-Cas9. Simultaneously, we mutated CTG to CCG (Fig. 1i) and obtained two edited clonal cell lines, BT549-66CTG#24 and BT549-66CCG#15 (Fig. 1j, k). SRB (Fig. 1l) and colony formation (Fig. 1m, n) assays revealed that the loss of 66CTG expression significantly inhibits the proliferation of TNBC cells. These findings indicate that 66CTG promotes the proliferation of TNBC cells independently of CDKN2B-AS1.

### 66CTG promotes TNBC cells to cross from G1 into S phase by enhancing Cyclin D1 expression

**Fig. 2:**
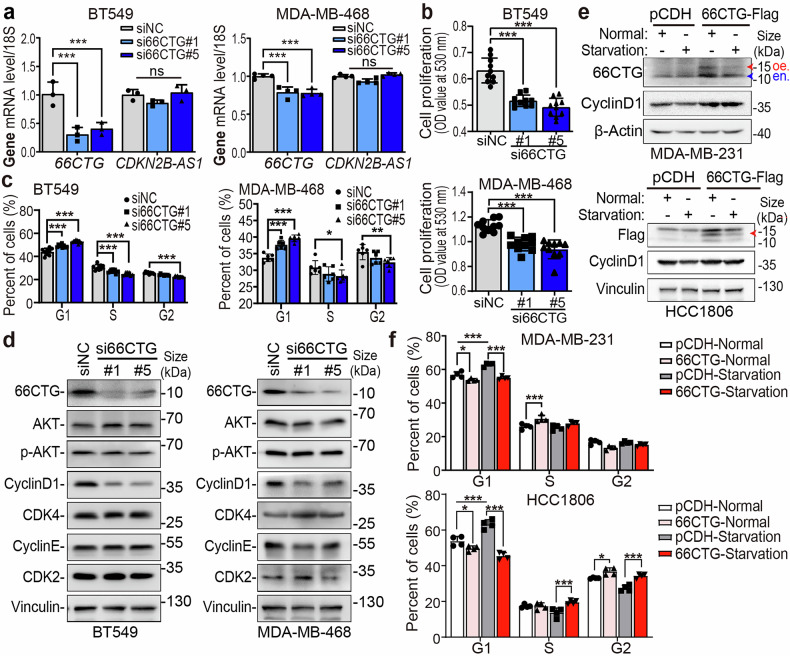
66CTG promotes TNBC cell proliferation by upregulating Cyclin D1. **a** qPCR detects the knockdown of 66CTG in BT549 (*n* = 3) and MDA-MB-468 (*n* = 4) cell lines. Error bars show the mean ± SD, “ns” means no significant, ***P < 0.001 by two-way ANOVA followed by Dunnett’s tests. **b** Using SRB assays to detect the proliferation of BT549 and MDA-MB-468 cells when 66CTG is knocked down (*n* = 10). Error bars show the mean ± SD, ***P < 0.001 by one-way ANOVA followed by Dunnett’s tests. **c** Statistical results of cell cycle of BT549 (*n* = 9) and MDA-MB-468 (*n* = 6) when 66CTG is knocked down. Error bars show the mean ± SD, *P < 0.05, **P < 0.01, ***P < 0.001 by two-way ANOVA followed by Dunnett’s tests. **d** WB analysis of G1 phase-related regulatory proteins in BT549 and MDA-MB-468 cells with 66CTG knocking down. **e** WB analysis of Cyclin D1 in MDA-MB-231 and HCC1806 cells with 66CTG-3×Flag overexpressing, followed by serum starvation for 36 h. The red arrow and “oe” indicate the position of 66CTG-3×Flag, and the blue arrow and “en” indicate the endogenous 66CTG. **f** Statistical results of cell cycle of MDA-MB-231 and HCC1806 cells with 66CTG-3×Flag overexpression followed by serum starvation for 36 h (*n* = 4). Error bars show the mean ± SD, *P < 0.05, ***P < 0.001 by two-way ANOVA followed by Dunnett’s tests

To explore the function of 66CTG in the proliferation of cancer cells, we prepared a specific antibody for 66CTG, and utilized two siRNAs to specifically knock down 66CTG expression in two cell lines of TNBC, including BT549 and MDA-MB-468 (Fig. 2a, d), as the expression levels of the 66CTG-encoding gene *CDKN2B-AS1* are relatively high in these two cell lines (Supplementary Fig. 1g). Then, we performed the SRB assay and analyzed cell cycle progression via flow cytometry. These results revealed that 66CTG silencing suppressed the proliferation of cancer cells (Fig. 2b) and caused cells to be arrested in the G1 phase (Fig. 2c). Among the regulators which are playing a crucial role at the G1/S checkpoint, the expression of Cyclin D1 was significantly downregulated following 66CTG knockdown (Fig. 2d). We treated MDA-MB-231-66CTG-3×Flag and HCC1806-66CTG-3×Flag cells with serum starvation for 36 h (Fig. 2e). Flow cytometry results indicated that serum starvation-induced G1 phase arrest; however, ectopic overexpression of 66CTG allowed cancer cells to withstand starvation stress and progress through the G1/S checkpoint into the S phase (Fig. 2f). This phenomenon may be associated with the upregulation of Cyclin D1 (Fig. 2e). These findings indicate that 66CTG is a critical regulator of TNBC cells during the transition of G1 to S, with Cyclin D1 acting as an important downstream factor in the proliferative function of 66CTG.

### 66CTG enhances Cyclin D1 transcription by stabilizing c-Myc in TNBC

**Fig. 3:**
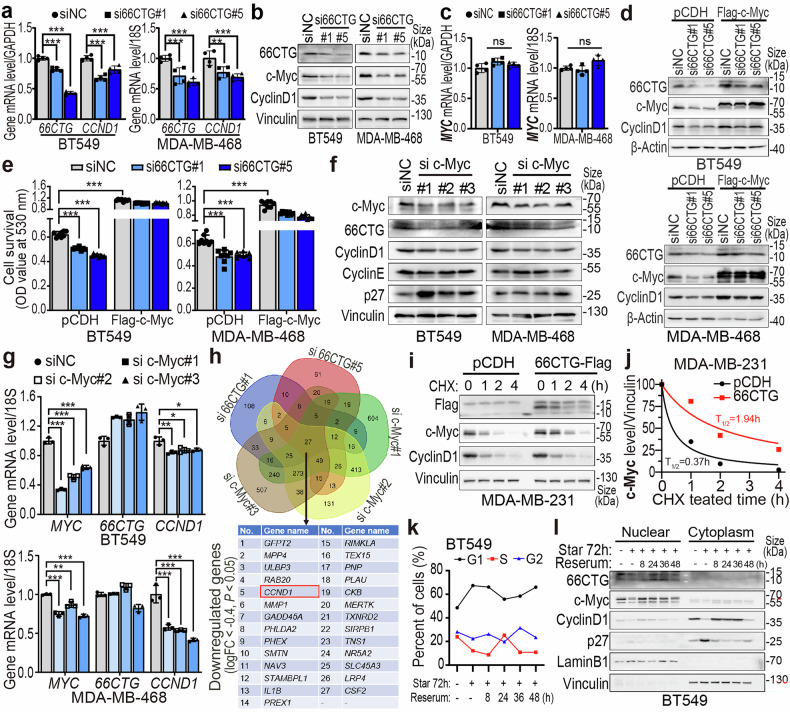
66CTG upregulates Cyclin D1 by stabilizing the protein level of c-Myc. **a** qPCR detection of transcription levels of 66CTG and Cyclin D1 in BT549 and MDA-MB-468 cells with 66CTG knocking down (*n* = 4). Error bars show the mean ± SD, **P < 0.01, ***P < 0.001 by two-way ANOVA followed by Dunnett’s tests. **b** WB analysis of c-Myc and Cyclin D1 in BT549 and MDA-MB-468 cells with 66CTG knocking down. **c** qPCR detection of transcription level of c-Myc in BT549 and MDA-MB-468 cells with 66CTG knocking down (*n* = 4). Error bars show the mean ± SD, “ns” means no significant by two-way ANOVA followed by Dunnett’s tests. **d** WB analysis of Cyclin D1 in BT549 and MDA-MB-468 cells with 3×Flag-c-Myc overexpression, followed by 66CTG knocking down. **e** Using SRB assays to detect the proliferation of BT549 and MDA-MB-468 cells with 3×Flag-c-Myc overexpressing followed by 66CTG knocking down (*n* = 8). Error bars show the mean ± SD, ***P < 0.001 by one-way ANOVA followed by Dunnett’s tests. **f** WB analysis of 66CTG and Cyclin D1 in BT549 and MDA-MB-468 cells with c-Myc knocking down. **g** qPCR detection of transcription level of c-Myc, 66CTG, and Cyclin D1 in BT549 and MDA-MB-468 cells with c-Myc knocking down (*n* = 3). Error bars show the mean ± SD, *P < 0.05, **P < 0.01, ***P < 0.001 by two-way ANOVA followed by Dunnett’s tests. **h** Downregulated genes were identified by RNA sequencing in BT549 cells of 66CTG or c-Myc knockdown, in which 27 co-downregulated genes were showed (logFC < −0.4, and *P* < 0.05). The venn diagram was generated by Draw Venn Diagram. **i** WB analysis of c-Myc protein expression levels in MDA-MB-231 cells after overexpressing 66CTG-3×Flag and subsequent treatment with CHX (50 μg/ml) for 0, 1, 2, 4 h. **j** The grayscale values of c-Myc protein levels and the fitting results of the half-life curve of (**i**). **k** Cell cycle analysis of BT549 cells subjected to serum starvation for 72 h, followed by reserum treatment for 8, 24, 36, and 48 h. “Star” means starvation. **l** After subjecting BT549 cells to serum starvation for 72 h, followed by reserum treatment for 8, 24, 36, and 48 h, the distribution and expression of 66CTG, c-Myc, and Cyclin D1 proteins in the nucleus and cytoplasm were analyzed using Western blotting. “Star” means serum starvation

To elucidate the mechanism of 66CTG upregulating Cyclin D1, we analyzed the transcription of the *CCND1* gene, which encodes Cyclin D1, following 66CTG knockdown. RT-qPCR results indicated that 66CTG knockdown significantly inhibited *CCND1* transcription (Fig. 3a and Supplementary Fig. 3a, b), and 66CTG overexpression enhanced *CCND1* transcription (Supplementary Fig. 2b). Since c-Myc is well known to increase the transcription of the *CCND1* gene during the late G1 phase.^42,43^ We examined c-Myc expression and found that 66CTG silencing significantly downregulated c-Myc protein level in both BT549 and MDA-MB-468 cell lines (Fig. 3b), 66CTG overexpression upregulated c-Myc protein level in both MDA-MB-231 and HCC1806 cells (Supplementary Fig. 2a). However, RT-qPCR results indicated that neither 66CTG knockdown nor overexpression affected the transcription of the *MYC* gene (Fig. 3c, and Supplementary Figs. 3a, b, 2b). To investigate whether the mechanism of 66CTG regulating Cyclin D1 expression and cell proliferation is through c-Myc, we overexpressed 3xFlag-c-Myc and knocked down 66CTG in BT549 and MDA-MB-468 cells. It turned out that c-Myc overexpression markedly rescued the inhibition of 66CTG silencing on Cyclin D1 expression (Fig. 3d) and cell proliferation (Fig. 3e).

Upon c-Myc overexpression, we observed a restoration of Cyclin D1 protein levels and an upregulation of 66CTG protein levels, which reversed the effects of 66CTG knockdown (Fig. 3d). To explore the potential mutual regulation between 66CTG and c-Myc, we utilized siRNAs to silence the expression of c-Myc in BT549 and MDA-MB-468 cells and found that c-Myc knockdown, similar to 66CTG knockdown, significantly induced G1 phase arrest in cancer cells (Supplementary Fig. 2c–f). As expected, c-Myc knockdown led to a downregulation of both CyclinD1 and 66CTG proteins (Fig. 3f). However, RT-qPCR results indicated that c-Myc knockdown significantly downregulated Cyclin D1 transcription, with no effect on 66CTG transcription (Fig. 3g and Supplementary Fig. 3a, b). These indicate that 66CTG and c-Myc regulate the expression of each other at the level of post-transcription. In addition, *CCND1*, identified as their downstream gene, was further validated through RNA-seq analysis conducted after the knockdown of 66CTG or c-Myc in BT549 cells (Fig. 3h).

Since c-Myc is primarily degraded at the protein level via the ubiquitin-proteasome pathway,^29^ we used siRNAs to knock down 66CTG in BT549 and MDA-MB-468 cells and subsequently treated these cells with 20 µM MG132 (a proteasome inhibitor) for 6 h. MG132 effectively restored the c-Myc downregulation induced by 66CTG knockdown (Supplementary Fig. 2g). Additionally, we treated MDA-MB-231 cells overexpressing 66CTG-3xFlag with 50 µg/ml cycloheximide (CHX, a protein synthesis inhibitor) for 0, 1, 2, and 4 h. We showed that 66CTG overexpression significantly prolonged the protein degradation half-life of c-Myc (Fig. 3i, j). These results indicate that 66CTG upregulates c-Myc expression by enhancing the stability of its protein.

Similarly, we used three siRNAs to knock down c-Myc in BT549 and MDA-MB-468 cells and then used MG132 to treat these cells. The results of Western blot showed that MG132 could reverse the downregulation of 66CTG caused by c-Myc knockdown (Supplementary Fig. 2h). Additionally, we performed CHX chase assay in BT549 cells stably overexpressing 3xFlag-c-Myc and demonstrated that c-Myc overexpression significantly prolonged the protein degradation half-life of 66CTG (Supplementary Fig. 2i, j). These findings imply that 66CTG and c-Myc can mutually stabilize each other at the protein level.

We treated BT549 cells with serum starvation for 72 h to arrest the cells at the G1 phase, followed by reserum for 8, 24, 36, and 48 h to release the cells into the progression of cell cycle (Fig. 3k and Supplementary Fig. 2k). The nuclear-cytoplasmic separation assay showed that 66CTG and c-Myc are mainly distributed in the nucleus. After reserum for 8, 24, and 36 h, the expression levels of c-Myc and Cyclin D1 were upregulated. Interestingly, the expression level of 66CTG was upregulated after reserum for 24 and 36 h, but remained down after reserum for 8 h when most cells were still at the G1 phase (Fig. 3l). These results suggest that at this time, 66CTG may be degraded to stabilize c-Myc, subsequently enhancing Cyclin D1 expression and promoting TNBC cells to cross the R checkpoint at the G1 phase.

### 66CTG promotes TNBC growth by upregulating the c-Myc/Cyclin D1 axis

**Fig. 4:**
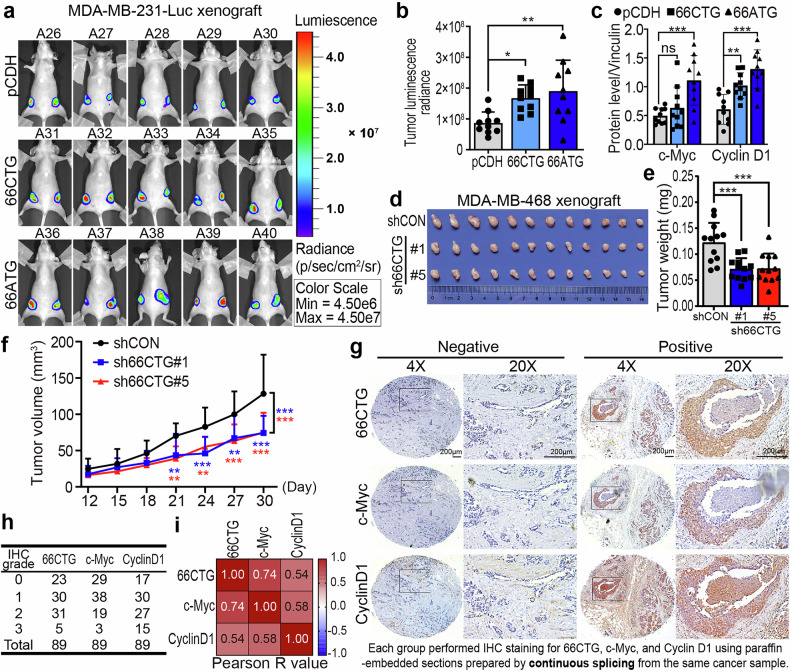
66CTG promotes TNBC tumor growth by upregulating the c-Myc/Cyclin D1 axis. **a** In vivo images of the orthotopic breast tumor model in nude mice established using MDA-MB-231-Luc-pCDH, MDA-MB-231-Luc-66CTG-3×Flag, and MDA-MB-231-Luc-66ATG-3×Flag cells. The original images could be found in Supplementary Dataset 9. **b** Statistical results of tumor luminescence radiance (*n* = 10). Error bars show the mean ± SD, *P < 0.05, **P < 0.01 by one-way ANOVA followed by Dunnett’s tests. **c** Statistical analysis of grayscale values for c-Myc and Cyclin D1 protein levels in (Supplementary Fig. 4e) (*n* = 10). Error bars show the mean ± SD, “ns” means no significant, **P < 0.01, ***P < 0.001 by two-way ANOVA followed by Dunnett’s tests. (**d**) Image of MDA-MB-468 xenograft with stable 66CTG knockdown. **e** Tumor weight of MDA-MB-468 xenograft with stable 66CTG knockdown (*n* = 12). Error bars show the mean ± SD, ***P < 0.001 by one-way ANOVA followed by Dunnett’s tests. **f** Statistical results of tumor volumes in MDA-MB-468 xenografts with 66CTG knockdown measured at different time points (*n* = 12). Error bars show the mean ± SD, **P < 0.01, ***P < 0.001 by two-way ANOVA followed by Dunnett’s tests. **g** Images of 66CTG, c-Myc, and Cyclin D1 expression in clinical TNBC paraffin-embedded continuous slicing samples using IHC. Scale bar: 200 μm. The original images could be found in Supplementary Dataset 10. **h** Pathological scoring results of 66CTG, c-Myc, and Cyclin D1 in 89 clinical TNBC paraffin samples. **i** Pearson correlation analysis of the expression levels of 66CTG, c-Myc, and Cyclin D1 in 89 clinical TNBC paraffin samples

To elucidate the oncogenic activity of 66CTG in vivo, a TNBC orthotopic xenograft tumor model was established by injecting the MDA-MB-231-Luc cells stably overexpressing 66CTG-3×Flag and 66ATG-3×Flag (Supplementary Fig. 4a) into the fourth pair of nude mice mammary fat pads. The reason for establishing the 66ATG-3×Flag overexpression group is that the expression level of pCDH-66ATG-3×Flag was higher than that of pCDH-66CTG-3×Flag (Supplementary Fig. 1f). We aim to explore whether the effect of 66CTG on regulating xenograft growth and the c-Myc/Cyclin D1 signaling pathway in tumor tissues will be enhanced with increased 66CTG expression levels. The results showed that 66CTG overexpression significantly promoted xenograft tumor growth according to the in vivo imaging (Fig. 4a, b), xenograft tumor sizes (Supplementary Fig. 4b, c), and tumor weight (Supplementary Fig. 4d). Meanwhile, 66CTG overexpression in xenograft tumor tissues enhanced the c-Myc and Cyclin D1 expression levels (Fig. 4c and Supplementary Fig. 4e). 66ATG-3×Flag exhibited a more potent regulatory effect on transplanted tumors and the c-Myc/Cyclin D1 signaling pathway compared to 66CTG-3×Flag. Specifically, in terms of regulating c-Myc expression level in the transplanted tumor tissues, although 66CTG-3×Flag showed a tendency to upregulate c-Myc level, this effect was not as significant as 66ATG-3×Flag (Fig. 4c and Supplementary Fig. 4e), as the expression of 66CTG-3×Flag was decreased with tumor growth. Additionally, we used the MDA-MB-468 cell line with stable 66CTG knockdown (Supplementary Fig. 4f, g) to generate a tumor model of TNBC orthotopic xenograft in nude mice, and found that the knockdown of 66CTG significantly suppressed the growth of TNBC xenograft tumor (Fig. 4d–f) and diminished the c-Myc and Cyclin D1 expression in xenograft tumor tissues (Supplementary Fig. 4h, i).

After validating the 66CTG antibody for IHC by using MDA-MB-231-66ATG-3xFlag cells (Supplementary Fig. 4j, k), we further explored the correlation among the expression of 66CTG, c-Myc, and CyclinD1 in clinical TNBC tissue samples. We stained paraffin-embedded tissue chips containing 89 cases of TNBC and demonstrated that there were significant correlations among 66CTG, c-Myc and CyclinD1, and the R-value of the correlation coefficient between 66CTG and c-Myc reached 0.74 (Fig. 4g–i). Additionally, as the pathological score of 66CTG increased in TNBC tissues, the pathological score of c-Myc also increased (Supplementary Fig. 4l).

### FBW7 mediates 66CTG and c-Myc degradation during the late G1 phase

**Fig. 5:**
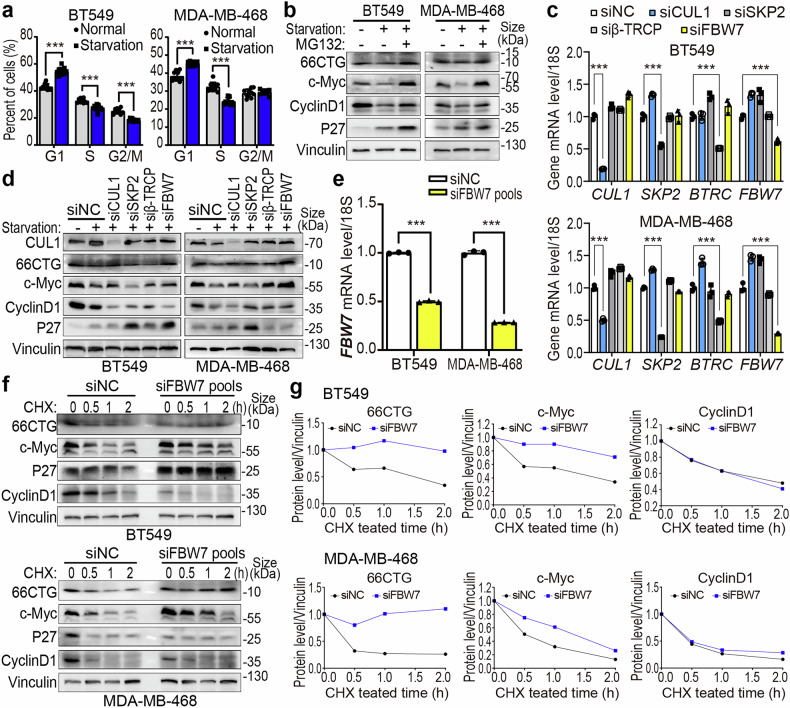
FBW7 mediates the degradation of c-Myc and 66CTG. **a** Statistical results of cell cycle of BT549 and MDA-MB-468 cells with serum starvation for 36 h (*n* = 10). Error bars show the mean ± SD, ***P < 0.001 by two-way ANOVA followed by Dunnett’s tests. **b** WB analysis of 66CTG and c-Myc in BT549 and MDA-MB-468 cells with serum starvation for 36 h, followed by treatment with MG132 (20 μM) for 6 h. **c** qPCR detection of the knockdown of CUL1, SKP2, β-TRCP, and FBW7 in BT549 and MDA-MB-468 cells (*n* = 3). Error bars show the mean ± SD, ***P < 0.001 by two-way ANOVA followed by Dunnett’s tests. **d** WB analysis of 66CTG and c-Myc in BT549 and MDA-MB-468 cells with knockdown of CUL1, SKP2, β-TRCP, and FBW7 followed by serum starvation for 36 h. **e** qPCR detection of the knockdown of FBW7 by using siRNA pools (siFBW7#1 and siFBW7#2) in BT549 and MDA-MB-468 cells (*n* = 3). Error bars show the mean ± SD, ***P < 0.001 by two-tailed Student’s *t*-test. **f** WB analysis of the pro*t*ein expression levels of 66CTG, c-Myc, and Cyclin D1 in BT549 and MDA-MB-468 cells with FBW7 knockdown, followed by treatment with CHX (50 μg/ml) for 0, 0.5, 1, 2 h. **g** The grayscale values of 66CTG, c-Myc, and Cyclin D1 protein levels and the protein degradation half-life curve of (**f**)

It’s known that mitotic signaling critically governs c-Myc protein stability, as attenuated mitogenic signals trigger its ubiquitination and proteasomal degradation.^29^ Interestingly, we found that serum starvation downregulated 66CTG expression (Figs. 2e, 3l). Therefore, we suspected that during serum starvation-induced G1 phase arrest, 66CTG is also degraded through the proteasome pathway. To test this, we subjected BT549 and MDA-MB-468 cells to serum starvation for 36 h, followed by the treatment of MG132 (20 μM) for 6 h. We showed that serum starvation-induced G1 phase arrest in cancer cells (Fig. 5a and Supplementary Fig. 5a) and diminished the protein levels of 66CTG, c-Myc, and Cyclin D1, which were blocked by MG132 (Fig. 5b).

During the late G1 phase or G1 phase arrest, SCF complex is the primary E3 ligase, mediating the ubiquitination and degradation of cell cycle regulatory proteins.^44,45^ To investigate whether 66CTG and c-Myc are co-regulated by this E3 complex at this stage, we employed siRNA to knock down the skeleton protein CUL1 and the adapter F-box proteins that recognize specific substrates (including SKP2, β-TRCP, and FBW7) in three TNBC cell lines, including BT549, MDA-MB-468, and MDA-MB-231-66ATG-3xFlag (Fig. 5c and Supplementary Fig. 5b), followed by 36 h of serum starvation. We found that knockdown of CUL1 and FBW7 rescued the downregulation of 66CTG and c-Myc induced by serum starvation (Fig. 5d and Supplementary Fig. 5c). To further confirm that FBW7 is the common F-box protein for 66CTG and c-Myc, we further performed a CHX chase assay, which revealed that FBW7 silencing (Fig. 5e and Supplementary Fig. 5d) significantly extended the degradation half-life of 66CTG and c-Myc proteins (Fig. 5f, g and Supplementary Fig. 5e, f). These results indicated that during serum starvation-induced G1 phase arrest, SCF^FBW7^ mediated the degradation of 66CTG and c-Myc proteins.

### 66CTG stabilizes c-Myc in TNBC through competitive interaction with FBW7α

c-Myc, as a substrate of FBW7, is highly expressed in TNBC, but the regulatory mechanisms of its expression remain unclear. A study involving 186 primary breast cancer cases reported that *FBW7* mRNA levels in TNBC were lower than in other breast cancer subtypes.^46^ However, by analyzing the TCGA and Metabric databases using bc-GenExMiner v5.0 and BCIP, we found that the transcription level of the *FBW7* gene in TNBC is higher than in non-TNBC (Supplementary Fig. 6a–d). Real-time PCR results also confirmed that the mRNA levels of *FBW7* in the cell lines of TNBC were higher than in the cell lines of non-TNBC (Supplementary Fig. 6e). This is interesting and valuable to further investigate the regulatory mechanism between FBW7 and c-Myc in TNBC.

**Fig. 6:**
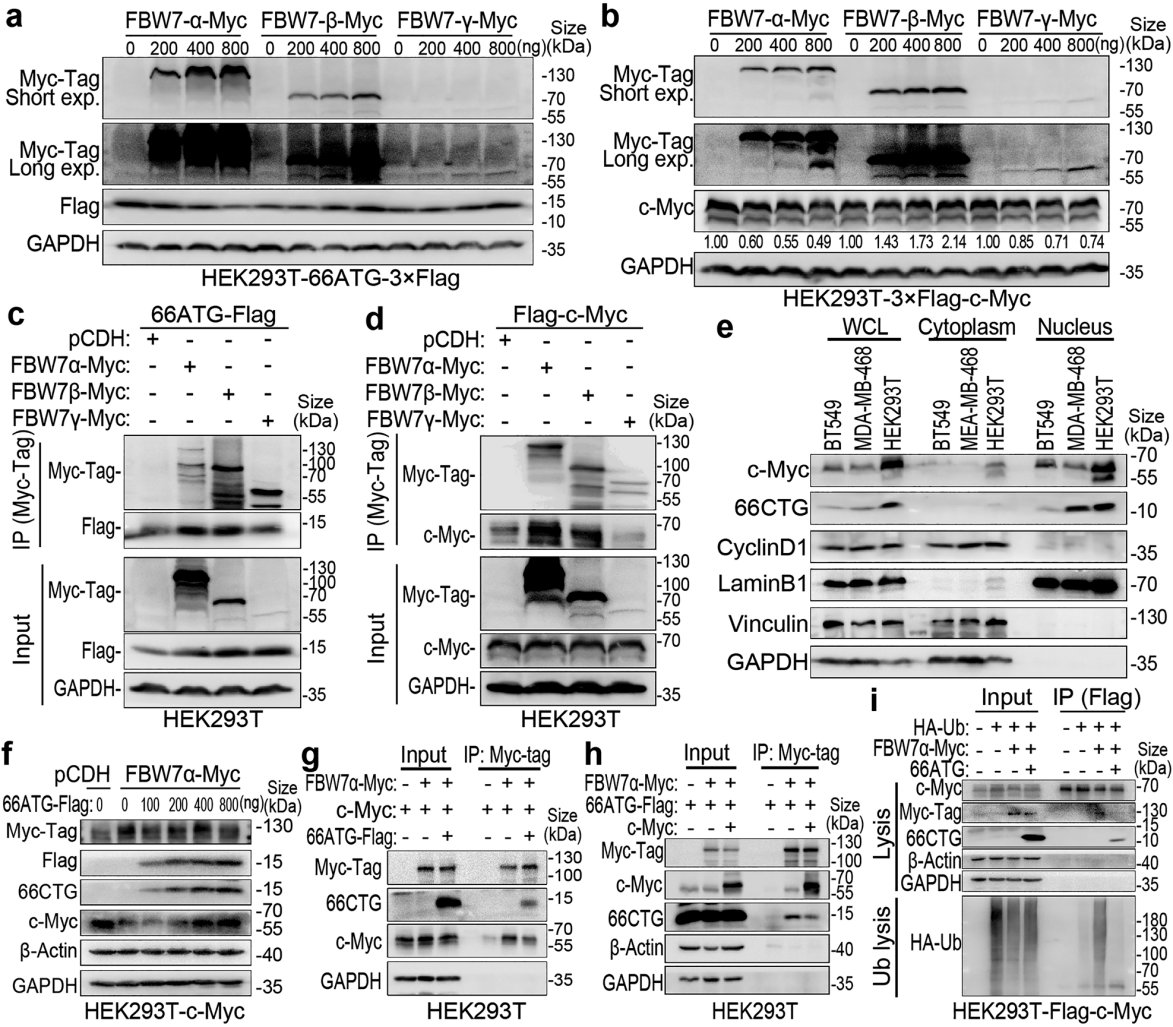
66CTG stabilizes c-Myc via interacting with FBW7α. **a** WB analysis of 66CTG in HEK293T-66ATG-3×Flag cells transfected with pCDH-FBW7α-Myc(tag), pCDH-FBW7β-Myc(tag), and pCDH-FBW7γ-Myc(tag) at serial doses of 0, 200, 400, and 800 ng. **b** WB analysis of c-Myc in HEK293T-3×Flag-c-Myc cells transfected with pCDH-FBW7α-Myc(tag), pCDH-FBW7β-Myc(tag), and pCDH-FBW7γ-Myc(tag) at serial doses of 0, 200, 400, and 800 ng. **c** IP and WB analyses of the interaction between 66CTG and three isoforms of FBW7 in HEK293T cells under MG132 (20 μM) treatment for 6 h. **d** IP and WB analyses of the interaction between c-Myc and three isoforms of FBW7 in HEK293T cells treated with MG132 (20 μM) for 6 h. **e** After the nuclear-cytoplasmic fractionation assay, the distribution of c-Myc and 66CTG protein in the nucleus and cytoplasm was analyzed using Western blot. “WCL” means whole cell lysate. **f** WB analysis of c-Myc expression levels in HEK293T-c-Myc (no tag) cells transfected with pCDH-FBW7α-Myc(tag) and varying doses of pCDH-66ATG-3×Flag (0, 100, 200, 400, and 800 ng). **g** IP and WB analyses of the interaction between c-Myc (notag) and FBW7α-Myc in HEK293T cells with or without 66CTG-3×Flag overexpression, treated with MG132 (20 μM) for 6 h. **h** IP and WB analyses of the interaction between 66CTG-3×Flag and FBW7α-Myc in HEK293T cells with or without c-Myc (notag) overexpression, treated with MG132 (20 μM) for 6 h. **i** IP and WB analyses of the ubiquitination level of c-Myc mediated by FBW7α in HEK293T-3×Flag-c-Myc cells with or without 66CTG overexpression

FBW7 has three isoforms: α (nucleoplasm), β (cytoplasm), and γ (nucleolus).^47^ To identify which isoform mediates the ubiquitination and degradation of 66CTG and c-Myc, we overexpressed all three isoforms together with 66ATG-3xFlag or 3xFlag-c-Myc into HEK293T cells. The results of western blot showed that only the isoform of nucleoplasmic FBW7α dose-dependently downregulated the 66CTG and c-Myc protein levels. In contrast, the cytoplasmic β isoform dose-dependently upregulated the levels of 66CTG and c-Myc (Fig. 6a, b). Meanwhile, immunoprecipitation (IP) experiments revealed significant interactions between both the FBW7α/β isoforms with 66CTG and c-Myc (Fig. 6c, d). However, given that 66CTG and c-Myc were primarily distributed in the nucleus (Fig. 6e) and FBW7β is known mainly to be localized in the cytoplasm, this may explain why the β isoform cannot degrade 66CTG and c-Myc.

We further investigated that FBW7α mediated c-Myc ubiquitination via its F-box (Supplementary Fig. 6f), and the overexpression of 66CTG could rescue the FBW7α-mediated degradation of c-Myc (Fig. 6f and Supplementary Fig. 6g). To determine whether 66CTG can competitively interact with FBW7α to stabilize c-Myc, we conducted competitive IP experiments and ubiquitination assays. The results showed that 66CTG overexpression significantly weakened the interaction between FBW7α and c-Myc (Fig. 6g), and also substantially reduced the FBW7α-mediated c-Myc ubiquitination (Fig. 6i). These indicate that 66CTG indeed stabilizes c-Myc by competitively binding to FBW7α.

Furthermore, we also found that c-Myc overexpression rescued the downregulation of 66CTG by FBW7α (Supplementary Fig. 6h) and weakened the interaction between FBW7α and 66CTG (Fig. 6h), which suggests that there is a mutually stabilizing protective effect between 66CTG and c-Myc when facing the degradation of FBW7α.

### FBW7α mediates 66CTG ubiquitination by recognizing its CPD^S56/S60^ motif

**Fig. 7:**
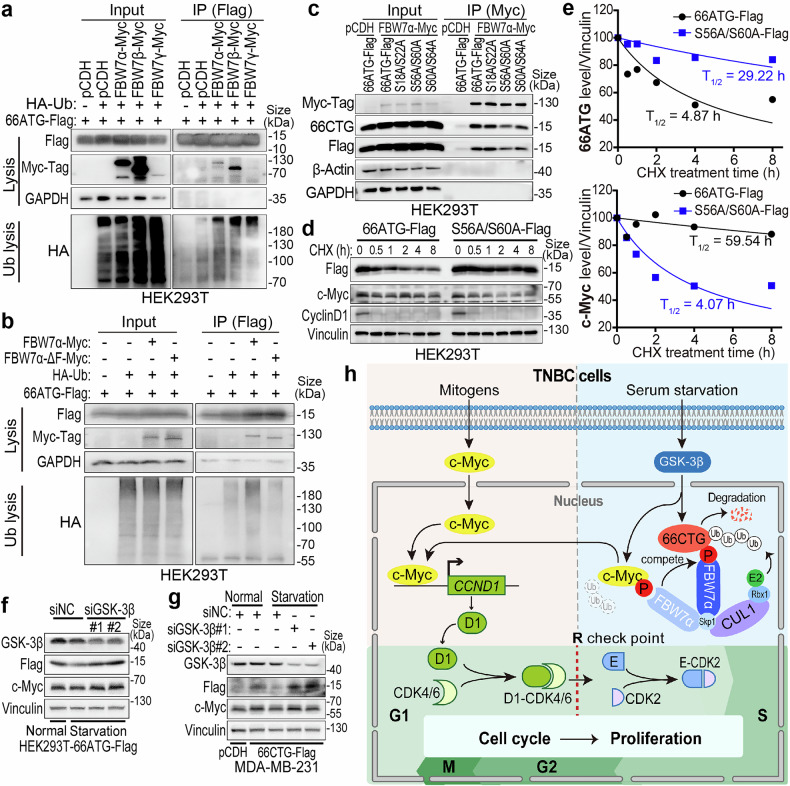
FBW7α mediates the ubiquitination and degradation of 66CTG through recognizing its CPD^S56/S60^ motif. **a** IP and WB analyses of the ubiquitination level of 66ATG-3×Flag mediated by three isoforms of FBW7 in HEK293T cells treated with MG132 (20 μM) for 6 h. **b** IP and WB analyses of the ubiquitination level of 66ATG-3×Flag mediated by FBW7α and FBW7α-ΔF-box in HEK293T cells treated with MG132 (20 μM) for 6 h. **c** IP and WB analyses of the interaction between FBW7α and 66CTG in HEK293T cells co-transfected with pCDH-FBW7α-Myc(tag) and pCDH-66ATG-3×Flag or its three CPD motif mutants. **d** WB analysis of 66CTG and c-Myc protein levels in HEK293T cells transfected with pCDH-66ATG-3×Flag or pCDH-66ATG^S56A/S60A^-3×Flag, followed by treatment with CHX (50 μg/ml) for 0, 0.5, 1, 2, 4, 8 h. **e** The grayscale values of 66CTG and c-Myc protein levels and the fitting results of the half-life curve of (**d**). **f** WB analysis of 66CTG and c-Myc protein levels in HEK293T-66ATG-3×Flag cells transfected with GSK-3β siRNAs, followed by serum starvation for 36 h. **g** WB analysis of 66CTG and c-Myc protein levels in MDA-MB-231-66CTG-3×Flag cells transfected with GSK-3β siRNAs, followed by serum starvation for 36 h. **h** The working model of this study was drawn by ourselves using Adobe Illustrator CS6. 66CTG stabilizes c-Myc by competitively interacting with FBW7α, thereby promoting the proliferation of TNBC cells. “D1” means Cyclin D1, and “E” means Cyclin E

We observed that FBW7α promotes the ubiquitination and degradation of 66CTG relying on its F-box domain (Fig. 7a, b and Supplementary Fig. 7a). Since FBW7 recognizes the CPD motifs of its substrates,^48^ we analyzed the amino acid sequence of 66CTG and identified three potential CPD motifs (including S18/S22, S56/S60, and S60/S64) (Supplementary Fig. 7b). We mutated the serine (S) residues at positions 0 and 4 of these motifs to alanine (A) and constructed their overexpression plasmids. As a result, 66CTG^S56A/S60A^ reduced its interaction with FBW7α (Fig. 7c). Furthermore, 66CTG^S56A/S60A^ showed resistance the ubiquitination (Supplementary Fig. 7c) and degradation (Supplementary Fig. 7d) mediated by FBW7α. Consistently, 66CTG^S56A/S60A^ was more stable and could no longer stabilize the c-Myc protein (Fig. 7d, e).

It is well known that CPD motifs recognized by FBW7 are usually phosphorylated by GSK-3β.^49–51^ Therefore, we knocked down GSK-3β in both HEK293T cells transiently overexpressing 66ATG-3xFlag and MDA-MB-231 cells stably overexpressing 66CTG-3xFlag, and found that GSK-3β knockdown blocked the downregulation of 66CTG induced by serum starvation (Fig. 7f, g). Similarly, the GSK-3β enzyme activity inhibitor LiCl also attenuated the downregulation of 66CTG induced by serum starvation (Supplementary Fig. 7e). These findings suggest that FBW7α mediates the ubiquitination and degradation of 66CTG by recognizing the CPD^S56/S60^ motif, which may be phosphorylated by GSK-3β.

## Discussion

TNBC is a malignant cancer that lacks specific therapeutic targets.^6^ c-Myc is a pivotal oncogenic driver in TNBC, and pharmacological inhibition of this target demonstrates anti-tumor efficacy.^19,22,23^ However, due to the physiological functions of c-Myc, the long-term application of its inhibitors is limited in TNBC treatment.^24^ Although c-Myc is overexpressed in TNBC, the underlying mechanisms remain unclear. Therefore, understanding the regulatory mechanisms of c-Myc overexpression in TNBC could lead to better therapeutic strategies targeting c-Myc and its associated pathways. Herein, we have discovered a novel peptide encoded by CDKN2B-AS1, which initiates translation with a CUG codon. This peptide promotes TNBC tumor growth by stabilizing the c-Myc protein through competitive interaction with FBW7α. Our findings not only reveal a new mechanism for c-Myc overexpression in TNBC but also provide a novel and reliable target for TNBC diagnosis and therapy.

### 66CTG exerts pro-oncogenic activity independent of CDKN2B-AS1

LncRNA CDKN2B-AS1 is highly expressed in clinical TNBC samples and is related to poor prognosis.^52^ Beyond breast cancer, CDKN2B-AS1 has also been implicated in promoting cancer in various tumor types.^52–55^ Several studies emphasize that the pro-cancer activity of CDKN2B-AS1 is through its ability to act as a sponge for microRNAs.^56–59^ Additional studies suggest that CDKN2B-AS1-mediated recruitment of transcription factors to oncogene promoters (including *NUF2* and *FSCN1*) potentiates transcriptional activation and accelerates malignant progression.^60,61^ We analyzed the sequence of CDKN2B-AS1 by using ORFfinder and SmProt online tools, and found that there was a small ORF encoding an unannotated peptide, 66CTG, using CUG as the start codon. When overexpressing the small peptide 66CTG in MDA-MB-231 and HCC1806 cell lines, we observed that 66CTG promoted cancer cell proliferation (Fig. 1c–f and Supplementary Fig. 1k–n). We further utilized CRISPR-Cas9 to mutate the start codon from CTG to CCG at the *66CTG* gene sequence in BT549 cell, and demonstrated that loss of 66CTG expression significantly inhibited cancer cell proliferation (Fig. 1i–n). These findings indicate that 66CTG promotes TNBC cell proliferation, which is independent of CDKN2B-AS1.

### Non-AUG initiation-coding products represent an underexplored area that warrants further investigation

The aggressive phenotype and target scarcity of TNBC render the discovery of specific therapeutic targets imperative. Recent advances in ribosome profiling, mass spectrometry peptidomics, and proteomics technologies have unveiled a significant presence of unannotated proteins in mammalian cells. The ORFs encoding these unannotated proteins are frequently located at the 5’ or 3’ ends of mRNA, as well as within lncRNAs and circRNAs, with a significant portion initiated by non-AUG codons.^7,9,17^ These unannotated proteins are not merely translation byproducts. Rather, they play crucial roles in many processes of physiology and pathology. For instance, the lncRNA LINC00961-encoded peptide, SPAR, is highly conserved in both human and mouse, and SPAR inhibits amino acid-mediated mTORC1 activation through the interaction with v-ATPase on the lysosomal membrane.^62^ Additionally, a small open reading frame at the 5’ end of the *MYC* gene mRNA encodes a secreted peptide, MPEP, composed of 114 amino acids that promotes glioma stem cell proliferation and tumor growth.^63^ The tumor suppressor protein PTEN has multiple isoforms translated from CUG or AUU start codons, including PTENα, β, and ε. Among these, PTENα regulates mitochondrial energy metabolism,^64^ PTENβ is involved in ribosomal DNA transcription and promotes ribosome biogenesis,^65^ and PTENε inhibits tumor metastasis by regulating filopodia formation.^66^ In this study, we revealed that CDKN2B-AS1 initiated the translation of the 66CTG peptide via CUG, which upregulated Cyclin D1 by stabilizing c-Myc, thereby promoting TNBC cell proliferation and tumor growth. Additionally, the expression level of 66CTG in clinical TNBC samples was not only highly elevated but also positively correlated with that of c-Myc and Cyclin D1. These findings suggest that unannotated proteins represent a significant resource for identifying specific diagnostic and therapeutic targets for malignant tumors.

### SCF^FBW7^ mediates the ubiquitination and degradation of 66CTG and c-Myc, which are phosphorylated by GSK-3β during the late G1 phase

During cell cycle, the protein level of c-Myc is tightly regulated by the SCF complex. Since SKP2 is degraded by the APC complex in the G1 phase,^67,68^ and interacts with c-Myc through the MB2 and bHLHZip domains^69^ rather than recognizing phosphorylated c-Myc, SKP2 primarily mediates the ubiquitination and degradation of c-Myc during the S phase. This is consistent with our finding that SKP2 silence did not restore the protein level of c-Myc during serum starvation-induced arrest of G1 phase (Fig. 5d and Supplementary Fig. 5c). In the late G1 phase, GSK-3β promotes c-Myc phosphorylation, and then FBW7 facilitates the ubiquitination and degradation of c-Myc by recognizing its CPD motifs (T58/S62 and T244/T248).^30,70^ This is also consistent with our observation that FBW7 knockdown significantly restored c-Myc protein levels during serum starvation-induced G1 phase arrest. Since the 66CTG peptide was also phosphorylated by GSK-3β in the late G1 phase (Fig. 7f, g and Supplementary Fig. 7e), FBW7 knockdown restored the protein level of 66CTG (Fig. 5d and Supplementary Fig. 5c). These could explain how 66CTG and c-Myc are able to stabilize each other during the late G1 phase.

This regulatory mechanism prompts us to consider a question: do all substrates of FBW7 have mutually stabilizing effects on each other? It is known that Cyclin E is also a substrate protein of FBW7 and promotes cells to transition from G1 to S phase. However, unlike c-Myc and 66CTG, Cyclin E degradation mainly occurs in the S phase.^71,72^ This is because the two phosphorylation sites on the CPD motif (T380/S384) of Cyclin E, which can be recognized by FBW7α, are mediated by GSK-3β and CDK2, respectively. During the late G1 phase, after Cyclin E is phosphorylated by GSK-3β at the T380 site, it needs to be further phosphorylated by CDK2 at the S384 site before it can be recognized by nucleoplasmic FBW7α. Despite recognizing Cyclin E, FBW7α does not mediate its ubiquitination. Instead, FBW7α cooperates with the prolyl *cis*-*trans* isomerase Pin1 to catalyze isomerization of the noncanonical proline (P381)-proline (P382) bond within the CPD^T380/S384^ motif of Cyclin E. This process is necessary for Cyclin E to be recognized by FBW7γ in the nucleolus after entering the S phase and to be ubiquitinated and degraded.^72,73^ In our research, neither 66CTG knockdown nor c-Myc knockdown affected the protein level of Cyclin E (Figs. 2d, 3f), which indicates that the mutual stabilization effect between 66CTG and c-Myc is specific. Therefore, the mutual stabilization of FBW7 substrate proteins depends on their recognition by the same FBW7 isoform at the same phase of cell cycle.

### FBW7α located in the nucleoplasm mediates the ubiquitination and degradation of 66CTG and c-Myc

The transcription products of the *FBW7* gene undergo alternative splicing at the N-terminus, generating three isoforms of protein: α, β, and γ. Besides their distinct N-terminal regions, all three FBW7 isoforms possess three common domains: the Dimerization domain (DD, facilitates the dimerization of FBW7), the F-box domain (interacts with SKP1), and the WD40 domain (engages with substrate proteins). Due to differences in the N-terminal signal peptides, the α isoform predominantly resides in the nucleoplasm, the β isoform is mainly located in the cytoplasm, and the γ isoform is primarily found in the nucleolus.^47^ It has been reported that overexpression of all three isoforms results in downregulation of c-Myc protein level, with the nucleoplasmic α isoform enhancing the ubiquitination of c-Myc phosphorylated by GSK-3β at the Thr58 site.^74^ In addition, the α and γ isoforms, but not the β isoform, interact with c-Myc, and the knockdown of the FBW7γ isoform promoted c-Myc accumulation in the nucleolus.^75^ These studies indicated that the FBW7α isoform mediates c-Myc degradation in the nucleoplasm and the FBW7γ isoform mediates c-Myc degradation in the nucleolus. Consistently, USP28 maintains c-Myc in the nucleoplasm by interacting with the FBW7α isoform.^76^ Meanwhile, USP36 stabilizes c-Myc in the nucleolus by interacting with the FBW7γ isoform.^77^ In our study, both 66CTG and c-Myc were predominantly distributed in the nucleus (Fig. 6e). Although the FBW7β isoform in the cytoplasm interacted with both 66CTG and c-Myc (Fig. 6c, d), it did not mediate their degradation (Fig. 6a, b). In contrast, the FBW7γ isoform in the nucleolus promoted c-Myc degradation, but its effect was less pronounced than that of the FBW7α (Fig. 6b). Additionally, the FBW7γ isoform did not downregulate 66CTG (Fig. 6a). However, the FBW7α isoform in the nucleoplasm not only significantly interacted with both 66CTG and c-Myc (Fig. 6c, d) but also dose-dependently downregulated their protein levels (Fig. 6a, b). Overexpression of 66CTG weakened the interaction between c-Myc and FBW7α (Fig. 6g) while increasing c-Myc protein levels in the presence of FBW7α (Fig. 6f and Supplementary Fig. 6g). These findings indicate that 66CTG is primarily localized in the nucleoplasm and competitively interacts with FBW7α, reducing the ubiquitin-mediated degradation of c-Myc, thereby stabilizing c-Myc protein level.

### 66CTG shows promise as a novel target for TNBC diagnosis and therapy

c-Myc, a substrate of FBW7, is highly expressed in TNBC. Unlike in other cancer types, FBW7 has a very low mutation rate in breast cancer.^32^ Although it has been reported that the transcription level of *FBW7* in TNBC was lower than in other breast cancer subtypes, our analysis and results showed that the transcription level of FBW7 in TNBC was higher than in non-TNBC (Supplementary Fig. 6a–e). So, it is very interesting and needed us to insight the mechanisms of c-Myc overexpression in TNBC. Here, we found that 66CTG stabilized c-Myc by competitive interaction with FBW7α, which further enhanced the Cyclin D1 transcription and promoted TNBC cell proliferation and tumor growth. By immunohistochemistry in clinical TNBC paraffin-embedded tissue samples, we also found that 66CTG, c-Myc, and Cyclin D1 were not only highly expressed in TNBC but also exhibited a strong positive correlation (Fig. 4g–i and Supplementary Fig. 4l). These findings indicate that 66CTG is a promising target for TNBC diagnosis and therapy. The development of small-molecule inhibitors targeting 66CTG holds significant potential. TNBC patients who overexpress 66CTG, c-Myc, and Cyclin D1 may benefit from a combined therapeutic approach using inhibitors of these molecules. Nevertheless, this hypothesis requires further clarification and validation in more clinical TNBC samples and through additional experiments. According to RNA-seq results, some of the 27 overlapping genes between 66CTG and c-Myc knockdown are associated with cell division, apoptosis, DNA damage, and transcription regulation (Fig. 3h). Due to the crucial role of c-Myc in cancer stemness, metastasis, and drug resistance, it is essential to further investigate the functions of 66CTG in TNBC metastasis and drug resistance. This will help determine whether 66CTG can serve as a target for TNBC treatment.

In conclusion, we demonstrated that the CDKN2B-AS1, which is highly expressed in TNBC, encoded the 66CTG peptide through CUG initiation. This peptide promoted TNBC cell proliferation, independent of CDKN2B-AS1. We also support a new mechanism of c-Myc overexpression in TNBC: 66CTG stabilized the c-Myc protein by supplying itself to FBW7α during the late G1 phase, thereby increasing Cyclin D1 transcription, promoting TNBC cell proliferation and tumor growth. As a novel peptide, 66CTG may be developed as a new target for TNBC diagnosis and therapy. Our findings may also help provide better treatment strategies for TNBC patients with co-overexpression of 66CTG, c-Myc, and Cyclin D1.

## Materials and methods

### Cell lines

The cell lines of HEK293T, MDA-MB-468, Hs578T and MCF7 were cultured in DMEM Medium (Gibco, Cat: C11995500BT) with 10% Fetal bovine serum (FBS, ExCell, Cat: FSD500). The cell lines of HCC1806 and HCC1937 were cultured in RPMI 1640 medium (Gibco, Cat: C11875500BT) with 10% FBS. The cell lines of BT549, BT474 and SK-BR-3 were cultured in 1640 medium with 10% FBS and 5 μg/ml Insulin (VivaCell Biosciences, Cat: C6010-1000). The cell line of SUM149PT was cultured in Ham’s F-12 medium (VivaCell, Cat: C3132-0500) with 5% FBS, 10 mM HEPES (Solarbio, Cat: H1095-100ml), 1 μg/ml Hydrocortisone (MedChemExpress, Cat: HY-N0583), and 5 μg/ml Insulin. The cell line of T47D was cultured in 1640 medium containing 10% FBS and 8 μg/ml Insulin. The cell lines of MDA-MB-231 and MDA-MB-231-Luc were cultured in DMEM/F12 (1:1) medium (Gibco, Cat: C11330500BT) containing 10% FBS. The cell line of MCF10A was cultured in DMEM/F12 (1:1) medium containing 5% horse serum (HS, Gibco, Cat: 16050122), 0.5 mg/ml Hydrocortisone, 20 ng/ml EGF (PeproTech, Cat: AF-100-15-100 μg), 10 μg/ml Insulin and 100 ng/ml cholera toxin (Beyotime, Cat: P7418-100 μg). All cell lines used in this study have been authenticated by STR analysis, and all cells were free from mycoplasma contamination and cultured in a humidified incubator (Thermo Scientific, Heracell 150i) at 37 °C with 5% CO_2_.

### Animals

The female BALB/c nude mice aged between 5 and 7 weeks were purchased from Slaccas (Changsha, Hunan, China). The feeding and experiments of animal were conducted in accordance with the protocol which is obtained the approvement of the animal ethics committee of Kunming Institute of Zoology, Chinese Academy of Sciences (IACUC-PA-2022-03-029).

### Patient samples and immunohistochemistry

All experiments (including RT-qPCR and IHC) involving in the clinical TNBC tissue samples were approved by the ethics committees of each participating hospital and Kunming Institute of Zoology, Chinese Academy of Sciences (KIZRKX-2022-012). The samples of tumor tissue were provided by patients with their agreement. The tissue samples of eight cases of clinical breast cancer (including cancer tissues and adjacent normal tissues) were obtained from the First Affiliated Hospital of Kunming Medical University. The paraffin-embedded tissue sample chips containing eighty-nine cases of triple-negative breast cancer were obtained from Henan Provincial People’s Hospital, which were prepared by continuous splicing. Hematoxylin-eosin (HE) staining and immunohistochemistry (IHC) were performed by the Pathology Department of The First Affiliated Hospital of Kunming Medical University using antibodies against 66CTG (GL Biochem, Cat: AB012233, 1.6 μg/ml), c-Myc (MedChemExpress, Cat: HY-P80626, 0.6 μg/ml), Cyclin D1 (ABclonal, Cat: A19038, 1 μg/ml). The specificity of the 66CTG antibody for IHC was confirmed by detecting the expression level of 66CTG in the MDA-MB-231-66CTG-3×Flag cell line (see Supplementary Fig. 4j, k for details). The interpretation and analysis of histopathological data were carried out by specialized pathologists. The expression levels of 66CTG, c-Myc, and Cyclin D1 were evaluated using the IRS Scoring Method, which combines the proportion of positive tumor cells (score range: 0 to 4) and staining intensity (score range: 0 to 3). The IRS total score is calculated as (Proportion Score) × (Intensity Score). Protein expression levels are categorized into four grades: (1) Grade 0, IRS score = 0, indicating negative expression (no detectable expression); (2) Grade 1, IRS score = 1–4, indicating low expression (weak or focal expression); (3) Grade 2, IRS score = 5–8, indicating moderate expression (intermediate expression); (4) Grade 3, IRS score = 9–12, indicating high expression (strong and diffuse overexpression). The clinicopathological data of 8 breast cancer and 89 TNBC tissue samples could be found in Supplementary Dataset 1 and 2, respectively. And the original images of immunohistochemical staining for 66CTG, c-Myc, and Cyclin D1 in the continuous splicing paraffin-embedded chips (related to Fig. 4g and Supplementary Fig. 4l) could be available from Supplementary Dataset 10.

### Cell transfection

For HEK293T cells, polyethyleneimine (PEI) (Polysciences, Cat: 24765-100) was used to transfect plasmids, and Lipofectamine 2000 (Thermo Fisher, Cat: 11668-019) was used to transfect siRNAs. For other breast cancer cell lines, Lipofectamine 2000 was used to transfect plasmids or siRNAs.

### Genome editing using CRISPR-Cas9 system

The sgRNAs targeting 66CTG were cloned into the LentiCRISPRv2 system. The ssODN (single-stranded DNA oligonucleotide) template was resuspended to a final concentration of 10 μM. HEK293T (2.5 × 10^5^/well) and BT549 (2.5 × 10^5^/well) cells were seeded in 12-well plates. On the next day, the LentiCRISPRv2-sgRNA (500 ng) and the ssODN template (1 μl) were co-transfected into either HEK293T or BT549 cells using Lipofectamine 2000. Forty-eight hours after transfection, cells were dissociated into single cell and diluted to a final concentration of 0.5 cells per 100 μl, before being seeded into 96-well plates. One week later, wells containing monoclonal cells were selected and allowed to expand for an additional 2–3 weeks. The cells were then harvested for RNA extraction. Screening of cloned cells with successfully inserted HA tag sequences was conducted using RT-qPCR. For positive clones, the corresponding bands were further amplified by PCR, and the amplified products were purified via gel recovery. The purified products were subsequently cloned into the pCE2-TA/Blunt-Zero vector, followed by sequencing for identification. The sequences of sgRNAs, ssODNs, and qPCR primers can be found in Supplementary Table 1.

### Gene overexpression (OE)

The DNA sequences of 66CTG and 66ATG, fused with a 3×Flag tag at the C-terminus, were cloned into the lentiviral vector of pCDH-CMV-MCS-EF1-puro (notag) at the BamHI and EcoRI restriction sites. This resulted in the construction of the plasmids pCDH-66CTG-3×Flag and pCDH-66ATG-3×Flag, facilitating the overexpression of 66CTG and 66ATG, respectively. The DNA sequence of c-Myc was cloned into the pCDH-CMV-3×Flag-MCS-EF1-puro (with the EcoRI and BamHI restriction sites) or pCDH-CMV-MCS-EF1-puro (notag) (with the BamHI and EcoRI restriction sites) lentiviral vector to create the plasmid pCDH-3×Flag-c-Myc or pCDH-c-Myc (notag) for the overexpression of c-Myc. A total of 5 μg of the overexpression lentiviral plasmid and 10 μg of packaging plasmids (including 5 μg pMDLg/pRRE, 3 μg pVSV-G, and 2 μg pRSV-Rev) were co-transfected into HEK293T cells using PEI. 48 h after transfection, the harvested viral supernatant was subsequently used to infect the TNBC cell lines, including MDA-MB-231, HCC1806, BT549, and MDA-MB-468. Puromycin selection was then carried out to obtain stable transfected TNBC cell lines that overexpressing 66CTG or c-Myc.

### Gene expression knockdown

Gene knockdown was achieved by using siRNA and shRNA. For transient transfection, siRNA is transfected into breast cancer cells with lipofectamine 2000. After 48 h of transfection, the gene knockdown is assessed using qPCR or Western blot assays. For stable knockdown, the shRNA sequence targeting 66CTG was cloned into the pSIH-H1-puro lentiviral vector. Subsequently, 2 µg of the pSIH-sh66CTG expression plasmid DNA, along with 3.3 µg each of the pPACKH1-GAG, pPACKH1-Rev, and pVSV-G packaging plasmids (totaling 12 µg), were transfected into HEK293T cells by using PEI to produce viral supernatant. MDA-MB-468 cell line was infected with this viral supernatant containing sh66CTG and subsequently selected with puromycin 48 h post-infection. The sequences of siRNAs and shRNAs can be found in Supplementary Table 1.

### Protein extraction and western blotting (WB) analysis

The lysis buffer (50 mM Tris-HCl pH 7.4, 1 mM EDTA, 150 mM NaCl, 1% Triton X-100) containing Protease Inhibitor Cocktail (MCE, Cat: HY-K0010) was used to extract the protein from cells and tumor tissues on ice for 30 min. WB was performed by using 11% SDS-PAGE gel and Thermo Scientific PageRuler Prestained Protein Ladder (Cat: #26616). The gel containing protein smaller than 35 KDa was transferred to 0.2 μm PVDF membrane (Merck millipore, Cat: ISEQ00010) at 100 V for 30 min, and the gel containing protein bigger than 35 KDa was transferred to 0.45 μm PVDF membrane (Merck millipore, Cat: IPVH00010) at 100 V for 90 min in cold room. After incubation with the primary and secondary antibodies, the substrate membrane was incubated in ECL solution (Abbkine, Cat: BMU102-CN) for 1 min and then was exposed on ImageQuant LAS 4000 gel imaging system (Cytiva, GE ImageQuant LAS 4000) and ChampChemi ® Chemiluminescent/Fluorescent/Gel Imaging and Analysis System (SINSAGE, cat: ChampChemi®910Plus). The detail information of the antibodies and reagents used in Western blotting is provided in Supplementary Table 2. The 66CTG antibody (rabbit polyclonal) was obtained from GL Biochem (Shanghai) Ltd. (Product No: AB012233). Its specificity was confirmed through 66CTG knockdown and overexpression experiments. The original and uncropped films of Western blot in this study could be found in Supplementary Dataset 8.

### RNA isolation and RT-qPCR assays

TRIzol (Thermo Fisher Scientific, Cat#15596026) was used to extract the total RNAs of cells and cancer tissues in accordance with its protocol. The concentration and quality of the extracted RNA were determined by a Nanodrop^TM^ 2000 Spectrophotometer (Thermo Fisher Scientific). Subsequently, total RNA was reverse transcribed into cDNA by using the HiScript IV 1^st^ Strand cDNA Synthesis Kit (+gDNA wiper) (Vazyme, Cat#R412-01) for long cDNA or the HiScript III RT SuperMix for qPCR (+gDNA wiper) (Vazyme, Cat# R323-01). qPCR assay was conducted using the Taq Pro Universal SYBR qPCR Master Mix (Vazyme, Cat# Q712-02) and detected on the Applied Biosystems 7900HT Fast Real-Time PCR System (Applied Biosystems, Cat# 4351405). The sequences of qPCR primers can be found in Supplementary Table 1.

### RNA sequencing

Total RNA of BT549 cells (5 × 10^5^ cells/sample) which have been transfected with the siRNAs of control (NC), 66CTG or c-Myc was extracted by using TRIzol (Thermo Fisher Scientific, Cat#15596026) to commercial RNA-seq analysis (Beijing Tsingke Biotech Co., Ltd., Beijing, China). The significantly differential expressions of genes were selected with log2 [Fold Change] < −0.4 and with *P*-value < 0.05. The analysis data could be found in Supplementary Dataset 3–7. The raw sequence data generated in this study have been archived in the Genome Sequence Archive (GSA; National Genomics Data Center, China National Center for Bioinformation) with the accession identifier HRA011629. These data can be accessed publicly via the GSA-Human portal: https://ngdc.cncb.ac.cn/gsa-human.

### Mass spectrometry and 4D label-free quantitative proteomics

The detection and analysis of the 4D label-free quantitative proteomics on endogenous 66CTG in BT549 cells were performed by Applied Protein Technology (Shanghai, China). Briefly, 5 × 10^6^ BT549 cells were harvested and lysed using 700 μl of SDT lysis buffer (100 mM Tris-HCl pH 7.6, 4% (w/v) SDS) with a Protease Inhibitor Cocktail (MCE, Cat: HY-K0010). The total protein was reduced and alkylated, then enzymatically digested into peptide fragments, followed by HPLC separation (nanoElute, Bruker). The separated peptide fragments were analyzed using a mass spectrometer (timsTOF Pro, Bruker). The analysis results of mass spectrometry were compared with the 66CTG sequence to determine the expression of endogenous 66CTG in cancer cells (OMIX ID: OMIX010222).

### SRB assays

For cell survival, BT549 (7 × 10^3^/well) and MDA-MB-468 (8 × 10^3^/well) cells were seeded in 96-well plates on the first day and transfected with siRNAs on the next day. Forty-eight hours post-transfection, the cells were fixed with Trichloroacetic acid (TCA, 10% (w/v)) (Macklin, Cat: T818878) for 30 min at room temperature. For cell proliferation, the 4 × 10^3^ cells (including BT549, MDA-MB-231, and HCC1806) were seeded for each well in 96-well plates. Twelve hours later, a portion of the cells was fixed with 10% TCA to serve as a seeding control. The remaining wells were fixed at 24, 48, 72, and 96 h, respectively. The fixed plates were then washed with ddH_2_O and air-dried at room temperature. Subsequently, the plates were stained with 0.04% (w/v) Sulforhodamine B sodium salt (SRB, 50 μl for each well) (Sigma-Aldrich, Cat: S1402) in 1% (v/v) acetic acid (Sangon Biotech, Cat: A501931) for 1 h at room temperature. After staining, the plates were washed five times with 1% (v/v) acetic acid in ddH_2_O and air-dried at room temperature. Finally, each well of plates was added 100 μl of 10 mM Tris base solution (pH 10.5), and then the plates were shaken for 20 min before measuring absorbance at 530 nm by using a microplate reader (Agilent, BioTek Epoch).

### Colony formation assays

A total of 1 × 10^3^ cells were seeded into each well of a 6-well plate, and then incubated in a CO_2_ incubator at 37 °C for 2–3 weeks to form colonies of substantial size (approximately 50 cells per colony). After washing with 1× PBS twice, cells were fixed with 4% paraformaldehyde (PFA) (Phygene, Cat: PH0427) for 30 min at room temperature. 1 ml of 0.5% (w/v) crystal violet solution (Solarbio, Cat: IC0600-100mg) was added to each well and used to stain the cells for 1 h at room temperature. The plate was washed five times with ddH_2_O and allowed to air-dry. After imaging, each well was added 800 μl of 33% (vol/vol) acetic acid to solubilize the dye, followed by shaking for 30 min. The absorbance was then measured by using a microplate reader (Agilent, BioTek Epoch) at 570 nm.

### Cell cycle analysis by flow cytometry

Each well of a 6-well plate was seeded with 2.0 × 10^5^ to 2.5 × 10^5^ cells. The next day, cells were transfected with siRNAs by using Lipofectamine 2000. Forty-eight hours after transfection, cells were collected and fixed with 75% ethanol for 30 min at room temperature (or stored for 1 to 2 weeks at 4 °C). Cells were then washed twice with 1× PBS and resuspended in 200 μl of 1× PBS containing 3 × 10^5^ cells. To treat the cells, 0.5 μl of 10 mg/ml RNase A (Beyotime, Cat: ST576) was added, followed by the addition of 3 ~ 5 μl of 10 mg/ml Propidium Iodide (PI) (Beyotime, Cat: ST1569-50mg) for staining in the dark for 30 min. After filtering the cells through a 200-mesh nylon membrane (Bioroyee, Cat: BH1811), flow cytometry was performed to analyze the cell cycle. Subsequently, FlowJo V10 software was used to analyze the data.

### Co-immunoprecipitation (co-IP) and ubiquitination assays

A total of 9 × 10^5^ HEK293T cells were seeded in a 6 cm dish, and then were transfected with plasmids by using PEI the following day. 48 h after transfection, MG132 (20 μM) was added to treat the cells for 4 h. Each dish of cells was lysed on ice with 400 μl of lysis buffer (50 mM Tris-HCl pH 7.4, 1 mM EDTA, 1% Triton X-100, 150 mM NaCl) with Protease Inhibitor Cocktail (MCE, Cat: HY-K0010) for 30 min. The protein lysate was divided into two equal portions: one portion is for normal co-IP; the other portion is for ubiquitination IP. The portion for ubiquitination IP was further lysed on ice for 15 min following the addition of 10 μl of 20% (w/v) SDS, and then boiled for 15 min to denature. After centrifugation at 12,000 rpm for 10 min at 4 °C, the supernatant of the portion for normal co-IP was collected in a new 1.5 ml tube. Some protein sample was prepared as the Input control. The remaining protein sample was incubated with Anti-Flag (MedChemExpress, Cat: HY-K0207) or Anti-Myc (MedChemExpress, Cat: HY-K0206) magnetic beads at 4 °C for 4–6 h. The immunoprecipitation (IP) complexes were washed with wash buffer (50 mM Tris-HCl pH 7.4, 150 mM NaCl, 0.1% NP-40, 2 mM EDTA pH 8.0) five times, each lasting 5 min with shaking at 4 °C. Each sample was added with 40 μL of 1x loading buffer and heated at 98 °C for 10 min to denature. The IP samples were analyzed by Western blotting (WB) using an 11% SDS-PAGE gel, and the samples of ubiquitination IP were analyzed by a 6% SDS-PAGE gel.

### Animal studies/breast orthotopic xenograft model

Female nude mice were anesthetized by using 7.5 mg/kg of xylazine and 75 mg/kg of ketamine. 2 × 10^5^ cells of MDA-MB-231-Luc (including MDA-MB-231-Luc-pCDH, MDA-MB-231-Luc-66CTG, and MDA-MB-231-Luc-66ATG), and 1 × 10^6^ cells of MDA-MB-468 (including MDA-MB-468-shCON, MDA-MB-468-sh66CTG#1, and MDA-MB-468-sh66CTG#5) were respectively resuspended in 100 μl of ice-cold medium/Matrigel (BD Biosciences, Cat: 354234) at a 1:1 ratio and orthotopically injected into the fourth pair of mammary fat pads. Using vernier caliper to measure the size of the transplanted tumors every 3 days from the eighth to the twelfth day after injection. The formula: tumor volume (cm³) = π (length × width²)/6 was used to calculate the tumor volume.

For the detection of bioluminescent, mouse was administered with D-luciferin (Bridgen, Cat: D12505) via intraperitoneal injection. 5 min later, the mouse was anesthetized with 7.5 mg/kg of xylazine and 75 mg/kg of ketamine. The IVIS Lumina XR system (Caliper Life Sciences, USA) was used to capture the bioluminescent images. The original bioluminescent images for Fig. 4a could be found in Supplementary Dataset 9. Each tumor tissue sample was divided into three parts: one for total RNA purification, one for protein extraction, and the third for paraffin embedding. In the xenograft model of MDA-MB-468, to ensure sufficient tumor tissue for paraffin samples, the left and right tumor tissues from the same mouse were pooled for RNA and protein extraction, as some tumors were too small to provide enough tissue individually.

### Quantification and statistical analysis

Data were analyzed by using the soft of GraphPad Prism 10.0. When the *P*-value is less than 0.05, the result can be considered statistically significant. In figures, the error bars represent the standard deviation derived from three or more independent experiments. Statistical significance was assessed using a two-tailed Student’s *t*-test, one-way or two-way ANOVA, followed by Dunnett’s test. The information of detailed statistics for each experiment is presented in the respective figure legends.

## Supplementary information

Supplementary Materials

Dataset 1

Dataset 2

Dataset 3

Dataset 4

Dataset 5

Dataset 6

Dataset 7

Dataset 8

Dataset 9

Dataset 10

## Data Availability

All data used to assess the conclusions in this study can be found in the article and supplementary materials. The raw data of RNA-seq (HRA011629) and the analysis data of proteomics (OMIX010222) can be available from GSA-Human and OMIX, respectively. In addition, the results based on TCGA data in this study were obtained from the third-party data analysis platform, including bc-GenExMiner v5.0 and BCIP.
