# Supplementary material for: A novel peptide 66CTG stabilizes Myc proto-oncogene protein to promote triple-negative breast cancer growth
Source: Signal Transduct Target Ther. 2025 Jul 9;10:217. doi: 10.1038/s41392-025-02298-5 (PMC12238259; doi:10.1038/s41392-025-02298-5)
Supplement: Supplementary file 1 — Supplementary Materials [file 41392_2025_2298_MOESM1_ESM.docx]

Supplementary Materials for

A novel peptide 66CTG stabilizes Myc proto-oncogene protein to promote triple-negative breast cancer growth

Huichun Liang, Fubing Li, Huan Fang, Wenlong Ren, Zhongmei Zhou, Jiecheng Wang, Jialing Liu, Yongjia Tang, Xue Liu, Yingying Wu, Jing Peng, Chuanyu Yang, Jiayi Chen, Yuting Fei, Yujie Shi, Dewei Jiang, Nu Zhang, Ceshi Chen

Correspondence to: chenc@kmmu.edu.cn (C.C.); zhangnu2@mail.sysu.edu.cn (N.Z.); or jiangdewei@mail.kiz.ac.cn (D.J.)

**This PDF file includes:**

Figures S1 to S7

Tables S1 to S4

Captions for Dataset 1 to 10

Figure. S1.

**
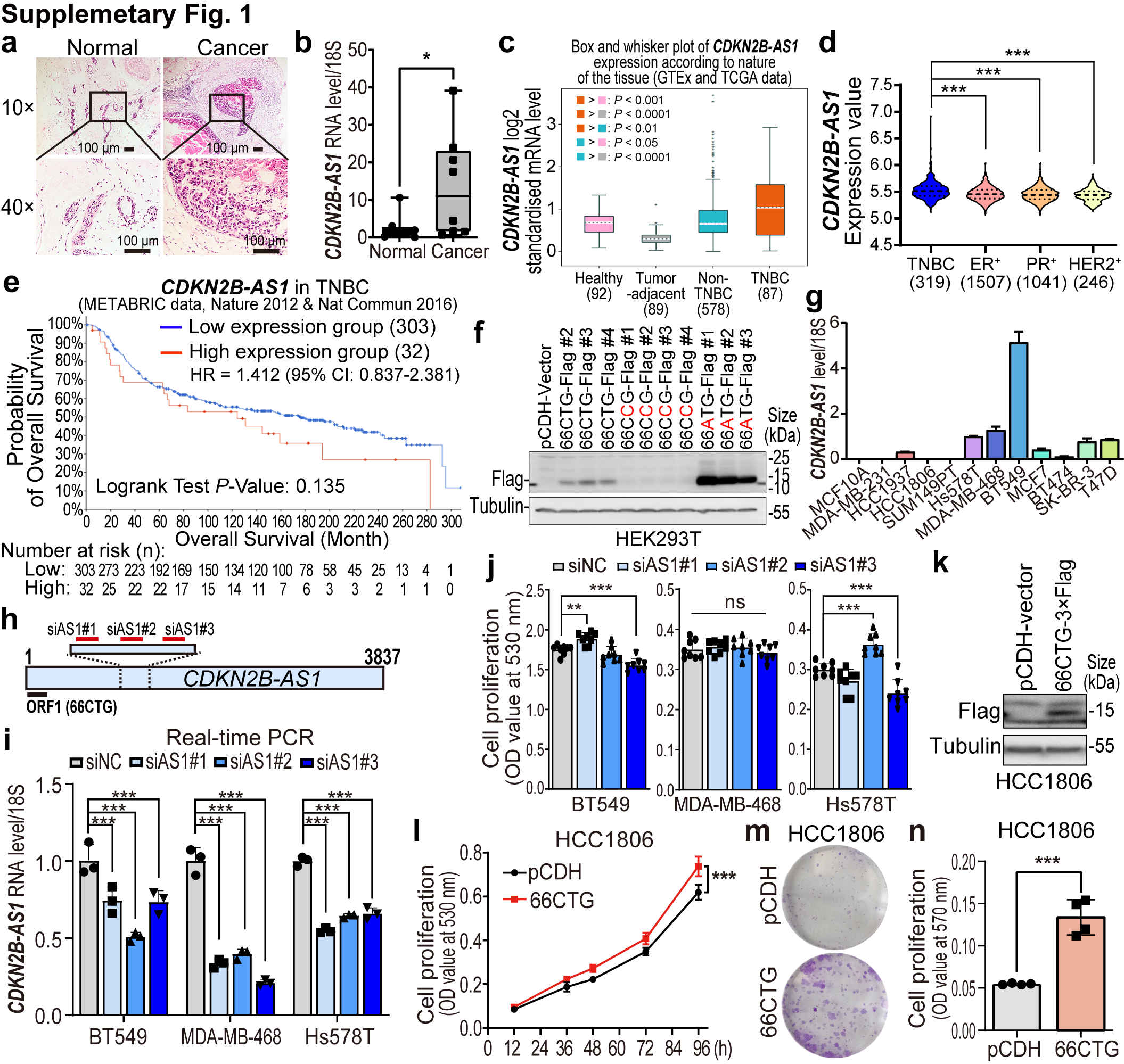
**

**Supplementary Figure 1. 66CTG encoded by CDKN2B-AS1 promotes TNBC cell proliferation. (a)** HE staining of 8 pairs of clinical breast cancer samples, including adjacent non-cancerous and cancerous tissues. Scale bar: 100 μm. **(b)** qPCR analysis of CDKN2B-AS1 expression levels in 8 pairs of clinical breast cancer samples. Error bars show the mean ± SD, * *P* < 0.05 by two-tailed Student’s *t* test. **(c)** Expression levels of CDKN2B-AS1 in Healthy (n = 92, Mean = 0.6684, SD = 0.2836), Tumor-adjacent (n = 89, Mean = 0.3188, SD = 0.1624), Non-TNBC (n = 578, Mean = 0.7639, SD = 0.4832), and TNBC (n = 87, Mean = 1.0548, SD = 0.7441) clinical samples from the GTEx and TCGA database via bc-GenExMiner v5.0. **(d)** Expression levels of CDKN2B-AS1 in TNBC (n = 319, Mean = 5.5317, SD = 0.1764), ER^+^ (n = 1507, Mean = 5.4598, SD = 0.1341), PR^+^ (n = 1041, Mean = 5.4520, SD = 0.1327), and HER2^+^ (n = 246, Mean = 5.4373, SD = 0.1223) clinical samples from the Metabric database via BCIP. Data were analyzed by using GraphPad Prism 10. *** *P* < 0.001 by one-way ANOVA followed by Dunnett’s tests. **(e)** Survival prognosis of TNBC patients with high CDKN2B-AS1 expression (n = 32) compared to those with low expression (n = 303) was analyzed by cBioPortal using the Metabric database (Nature 2012 & Nat Commun 2016). The grouping threshold was automatically generated by cBioPortal. **(f)** WB analysis of 66CTG expression encoded by ORF1 in HEK293T cells transfected with pCDH-66CTG-3×Flag, pCDH-66CCG-3×Flag or pCDH-66ATG-3×Flag. **(g)** qPCR analysis of CDKN2B-AS1 expression in breast cancer cell lines. **(h)** A schematic diagram of CDKN2B-AS1 illustrates three target sites for siRNA. “AS1” means CDNK2B-AS1. **(i)** CDKN2B-AS1 knockdown was detected in BT549, MDA-MB-468, and Hs578T cells via qPCR (n = 3). Error bars show the mean ± SD, *** *P* < 0.001 by two-way ANOVA followed by Dunnett’s tests. **(j)** The SRB assay assessed the impact of CDKN2B-AS1 knockdown on the proliferation of BT549, MDA-MB-468, and Hs578T cells (n = 8). Error bars show the mean ± SD, “ns” means no significant, ** *P* < 0.01, *** *P* < 0.001 by two-way ANOVA followed by Dunnett’s tests. **(k)** Overexpression of 66CTG-3×Flag was detected in HCC1806 cells via Western blotting. **(l)** SRB assays assessed the effect of 66CTG-3×Flag overexpression on the proliferation of HCC1806 (n = 6) cells. Error bars show the mean ± SD, *** *P* < 0.001 by two-way ANOVA. **(m)** The images of the colony formation of HCC1806 cells with 66CTG-3×Flag overexpression. **(n)** Statistical results of colony formation assay (n = 4) of (m). Error bars show the mean ± SD, *** *P* < 0.001 by two-tailed Student’s *t* test.

Figure. S2.

**
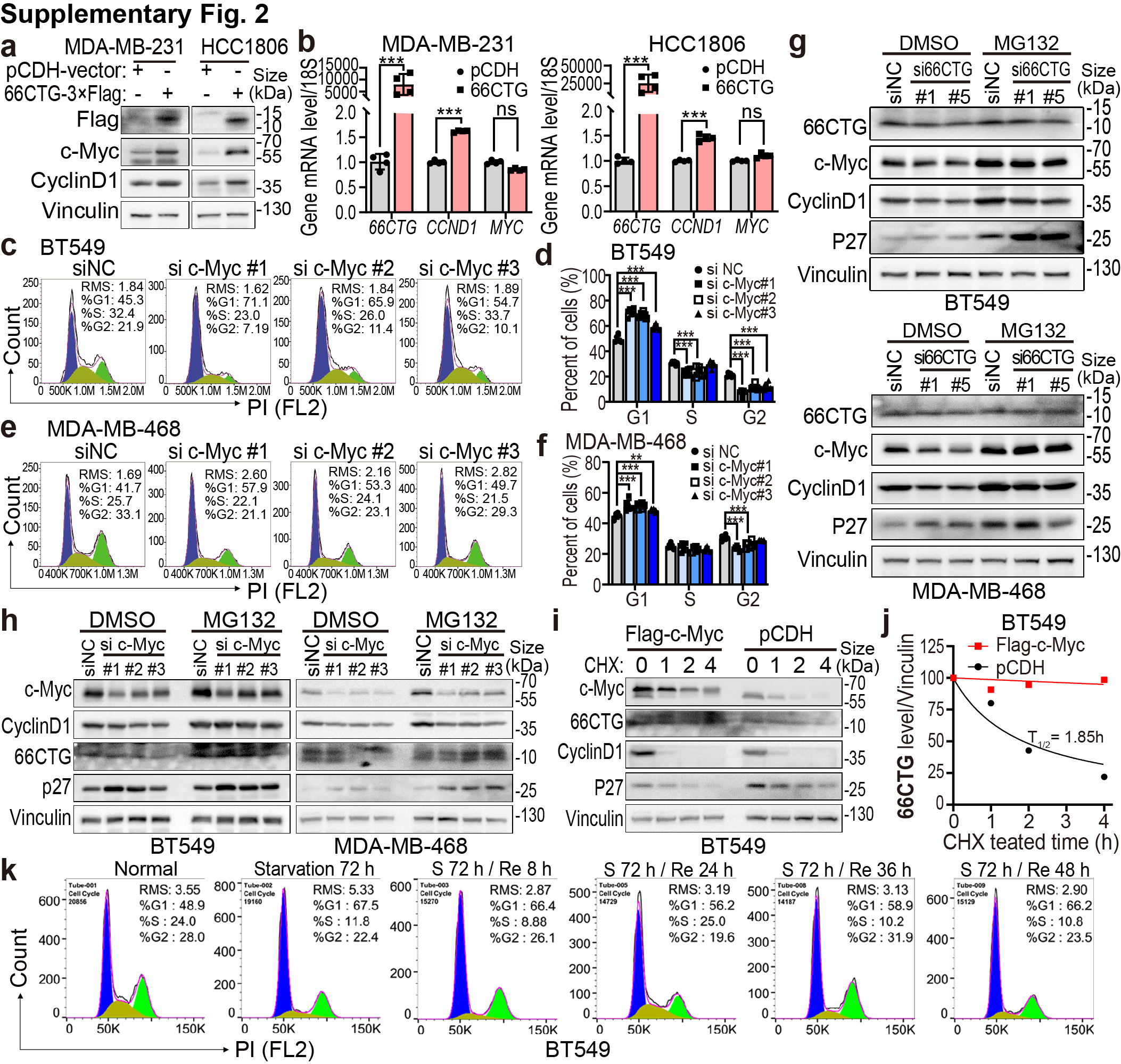
**

**Supplementary Figure 2. 66CTG upregulates Cyclin D1 through stabilizing c-Myc. (a)** WB analysis of c-Myc and Cyclin D1 in MDA-MB-231 and HCC1806 cells with 66CTG-3×Flag overexpressing. **(b)** qPCR detection of transcription levels of 66CTG, CyclinD1, and c-Myc in MDA-MB-231 and HCC1806 cells with 66CTG overexpressing (n = 4). Error bars show the mean ± SD, “ns” means no significant, *** *P* < 0.001 by two-way ANOVA followed by Dunnett’s tests. **(c)** Illustrated flow cytometry images showing cell cycle analysis of BT549 cells following c-Myc knockdown. **(d)** Statistical results of cell cycle of BT549 when c-Myc is knocked down in (c) (n = 6). Error bars show the mean ± SD, *** *P* < 0.001 by two-way ANOVA followed by Dunnett’s tests. **(e)** Illustrated flow cytometry images showing cell cycle analysis of MDA-MB-468 cells following c-Myc knockdown. **(f)** Statistical results of cell cycle of MDA-MB-468 when c-Myc is knocked down in (e) (n = 6). Error bars show the mean ± SD, *** *P* < 0.001 by two-way ANOVA followed by Dunnett’s tests. **(g)** WB analysis of c-Myc protein expression levels in BT549 and MDA-MB-468 cells after knocking down 66CTG and subsequent treatment with MG132 (20 μM) for 6 hours. **(h)** WB analysis of 66CTG protein expression levels in BT549 and MDA-MB-468 cells after knocking down c-Myc and subsequent treatment with MG132 (20 μM) for 6 hours. **(i)** WB analysis of 66CTG protein expression levels in BT549 cells after overexpressing 3×Flag-c-Myc and subsequent treatment with CHX (50 μg/ml) for 0, 1, 2, 4 hours. **(j)** The grayscale values of 66CTG protein levels and the fitting results of the half-life curve of (i). **(k)** Illustrated flow cytometry images showing cell cycle analysis of BT549 cells subjected to serum starvation for 72 hours, followed by reserum treatment for 8, 24, 36, and 48 hours. “S” means starvation, and “Re” means reserum.

Figure. S3.

**
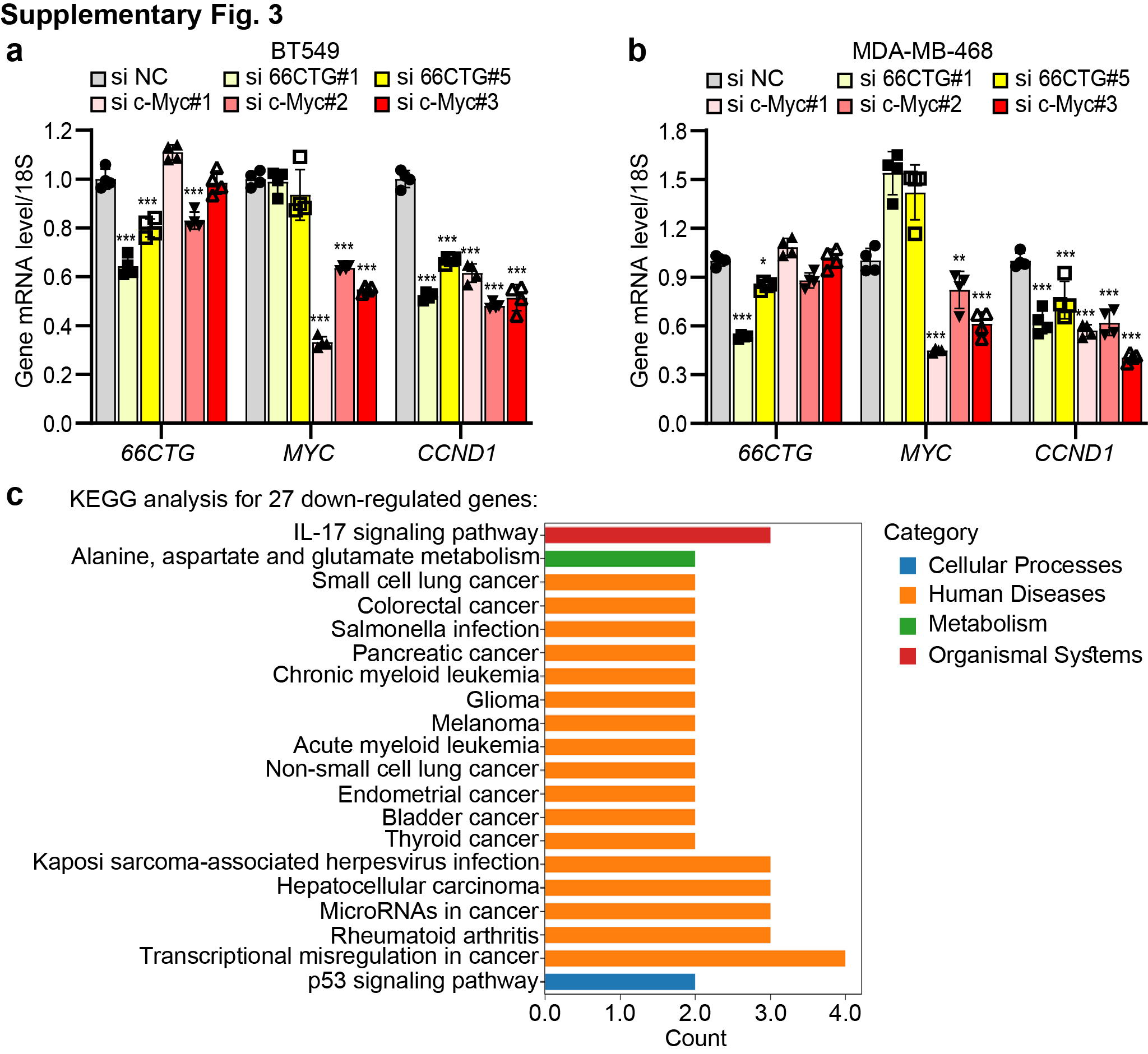
**

**Supplementary Figure 3. 66CTG promotes TNBC cell proliferation by up-regulating Cyclin D1.** **(a)** qPCR confirmed the *CCND1* transcription after 66CTG and c-Myc knocking down in BT549 cell line (n = 4). Error bars show the mean ± SD, *** *P* < 0.001 by two-way ANOVA followed by Dunnett’s tests. **(b)** qPCR confirmed the *CCND1* transcription after 66CTG and c-Myc knocking down in MDA-MB-468 cell line (n = 4). Error bars show the mean ± SD, * *P* < 0.05, ** *P* < 0.01, *** *P* < 0.001 by two-way ANOVA followed by Dunnett’s tests. **(c)** The KEGG enrichment analysis identified 27 genes that were concurrently downregulated in RNA-seq results following the knockdown of 66CTG and c-Myc in BT549 cells by using the online tool of KEGG enrichment analysis of Beijing Tsingke Biotech Co., Ltd.

Figure. S4.

**
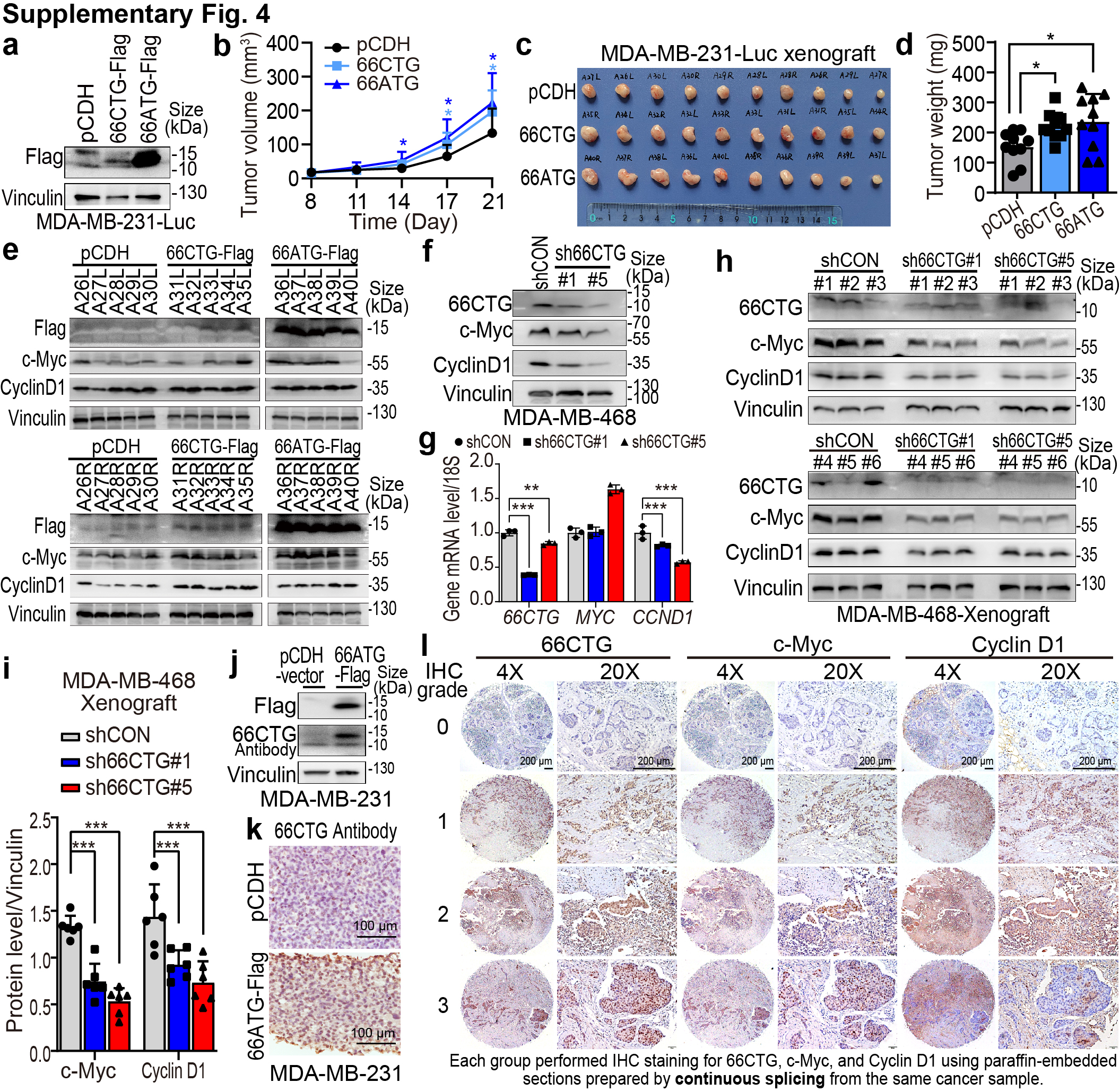
**

**Supplementary Figure 4. 66CTG promotes the TNBC tumor growth by upregulating the c-Myc/Cyclin D1 axis. (a)** WB analysis of 66CTG in MDA-MB-231-Luc with stable 66CTG-3×Flag and 66ATG-3×Flag expression. **(b)** Statistical results of tumor volumes in MDA-MB-231-Luc xenografts with 66CTG overexpression measured at different time points (n = 10). Error bars show the mean ± SD, * *P* < 0.05 by two-way ANOVA followed by Uncorrected Fisher’s LSD. **(c)** Image of MDA-MB-231-Luc xenograft with stable 66CTG overexpression. **(d)** Tumor weight of MDA-MB-231-Luc xenograft with stable 66CTG overexpression (n = 10). Error bars show the mean ± SD, * *P* < 0.05 by one-way ANOVA followed by Uncorrected Fisher’s LSD. **(e)** WB analysis of 66CTG, c-Myc, and Cyclin D1 in MDA-MB-231-Luc xenograft tissues. The protein samples from the 66ATG-Flag group and the protein samples from the pCDH and 66CTG-Flag groups were run on different gels, but the PVDF membranes of the same band from all three groups were incubated with the ECL substrate solution simultaneously and exposed together. **(f)** WB analysis of 66CTG, c-Myc, and Cyclin D1 in MDA-MB-468 cells with stable 66CTG knockdown. **(g)** qPCR detection of transcription levels of 66CTG, c-Myc and CyclinD1 in MDA-MB-468 cells with stable 66CTG knocking down (n = 3). Error bars show the mean ± SD, ** *P* < 0.01, *** *P* < 0.001 by two-way ANOVA followed by Dunnett’s tests. **(h)** WB analysis of 66CTG, c-Myc, and Cyclin D1 in MDA-MB-468 xenograft tissues with stable 66CTG knockdown. **(i)** Statistical analysis of grayscale values for c-Myc and Cyclin D1 protein levels in (h) (n = 6). Error bars show the mean ± SD, *** *P* < 0.001 by two-way ANOVA followed by Dunnett’s tests. **(j)** WB analysis of 66CTG by using 66CTG antibody in MDA-MB-231 with stable 66ATG-3×Flag expression. **(k)** IHC analysis of 66CTG expression by using 66CTG antibody in MDA-MB-231 with stable 66ATG-3×Flag expression. Scale bar: 100 μm. **(l)** IHC images of 66CTG, c-Myc, and Cyclin D1 expression in clinical TNBC paraffin-embedded continuous slicing samples according to the 66CTG pathological score. Scale bar: 200 μm. The original images could be found in Supplementary Dataset 10.

Figure. S5.

**
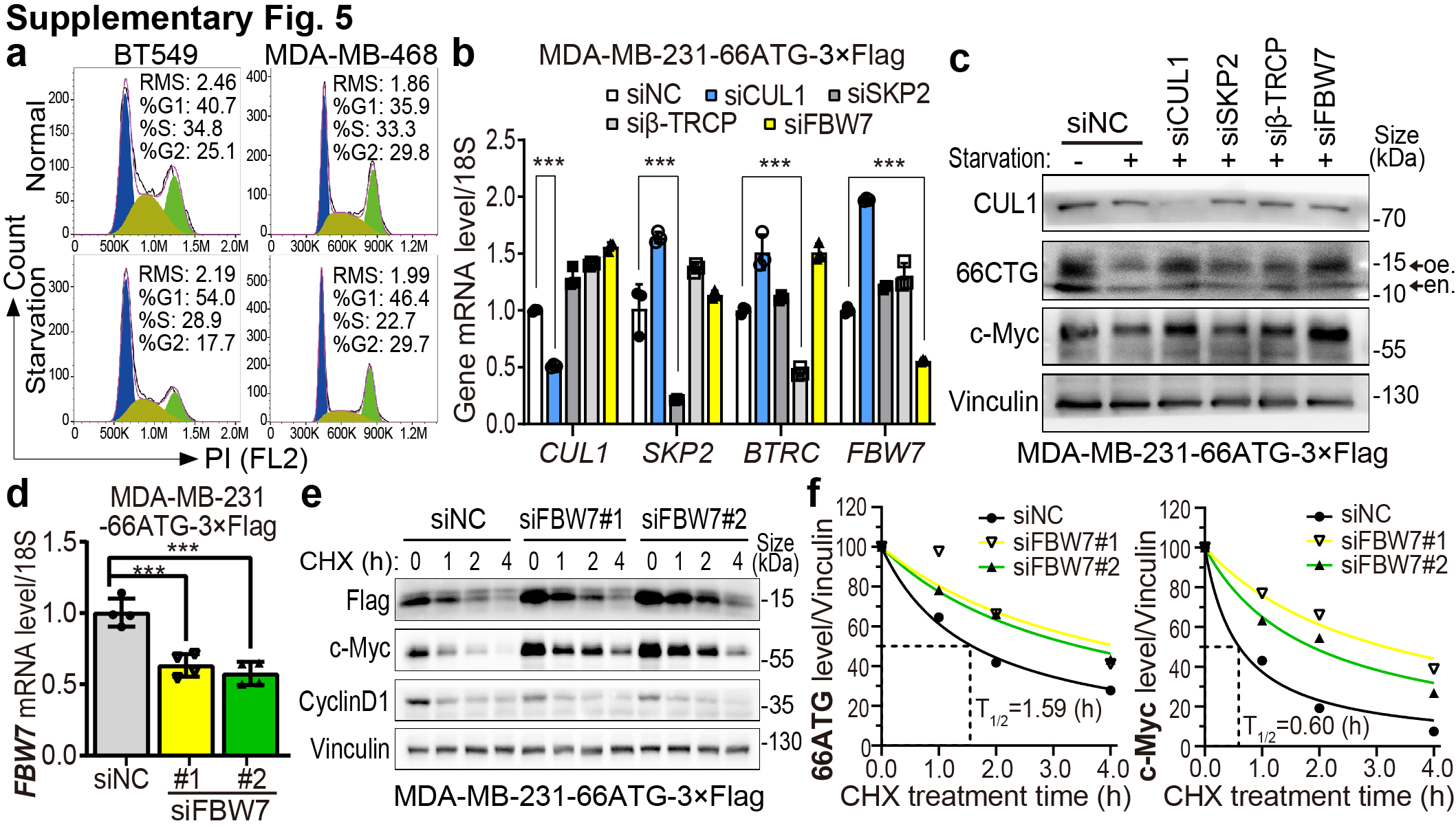
**

**Supplementary Figure 5. FBW7α mediates the ubiquitination and degradation of 66CTG via recognizing its CPD^S56/S60^ motif. (a)** Illustrated flow cytometry images showing cell cycle analysis of BT549 and MDA-MB-468 cells following 36 hours of serum starvation. **(b)** qPCR detection of the knockdown of CUL1, SKP2, β-TRCP, and FBW7 in MDA-MB-231-66ATG-3×Flag cells (n = 3). Error bars show the mean ± SD, *** *P* < 0.001 by two-way ANOVA followed by Dunnett’s tests. **(c)** WB analysis of 66CTG and c-Myc in MDA-MB-231-66ATG-3×Flag cells with knockdown of CUL1, SKP2, β-TRCP, and FBW7 followed by serum starvation for 36 hours. “oe.” means overexpressed 66CTG, and “en.” means endogenous 66CTG. **(d)** qPCR detection of the knockdown of FBW7 by using siRNAs in MDA-MB-231-66ATG-3×Flag cells (n = 4). Error bars show the mean ± SD, *** *P* < 0.001 by one-way ANOVA followed by Dunnett’s tests. **(e)** WB analysis of the protein expression levels of c-Myc, 66CTG, and Cyclin D1 in MDA-MB-231-66ATG-3×Flag cells with FBW7 knockdown followed by treatment with CHX (50 μg/ml) for 0, 1, 2, 4 hours. **(f)** The grayscale values of 66CTG and c-Myc protein levels and the fitting results of the half-life curve of (e).

Figure. S6.

**
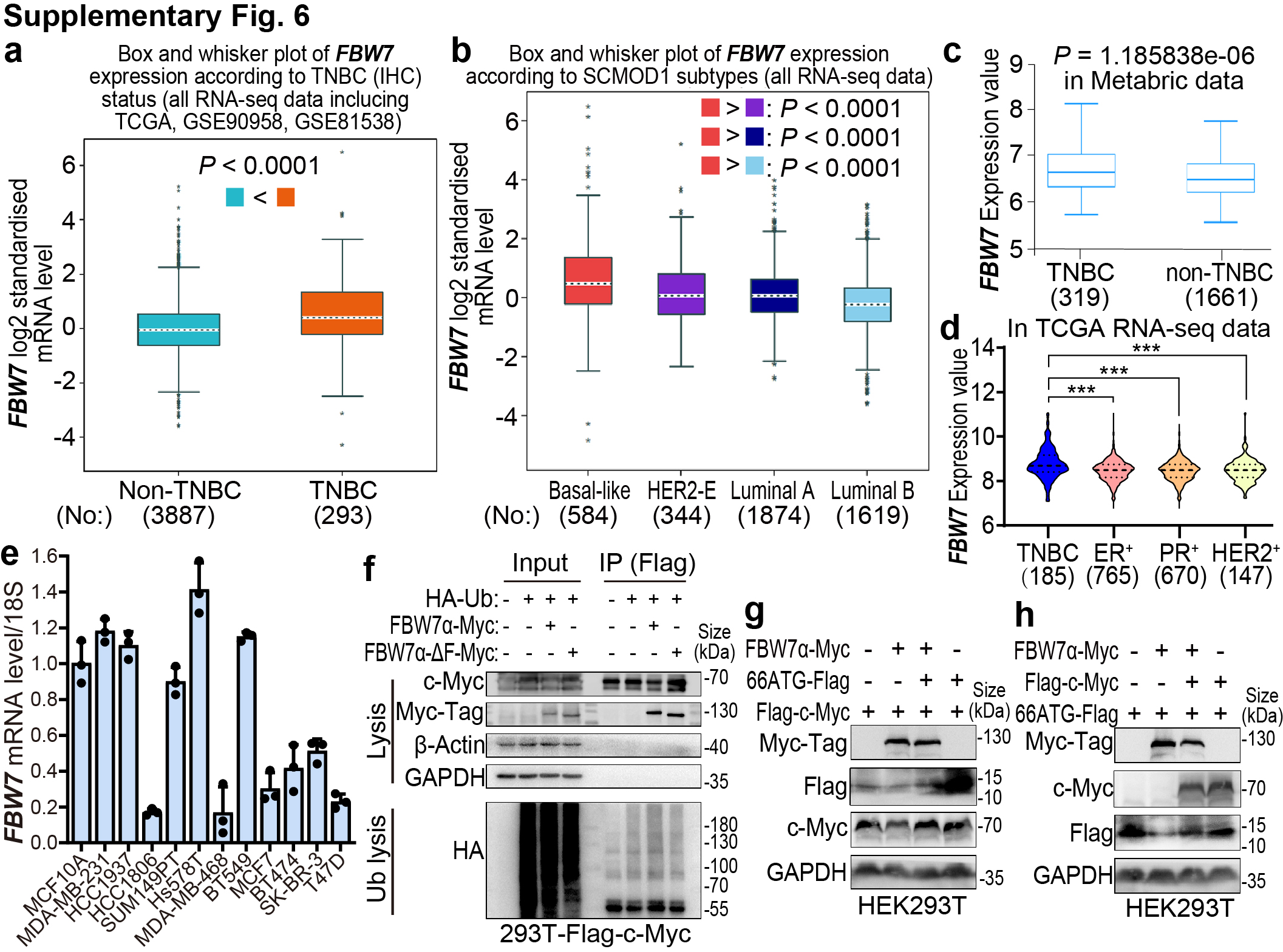
**

**Supplementary Figure 6. 66CTG stabilizes c-Myc via interacting with FBW7α. (a)** The transcription levels of *FBW7* gene in non-TNBC (n = 3887) and TNBC (n = 293) clinical samples from the TCGA, GSE90958, and GSE81538 databases, were analyzed using bc-GenExMiner v5.0. **(b)** The transcription levels of *FBW7* gene in Basal-like (n = 584), HER2-E (n = 344), Luminal A (n = 1874), and Luminal B (n = 1619) clinical samples from the TCGA, GSE90958, and GSE81538 databases, were analyzed using bc-GenExMiner v5.0. **(c)** The transcription levels of FBW7 in TNBC (n = 319) and non-TNBC (n = 1661) clinical samples from the Metabric database were analyzed by BCIP. **(d)** The transcription levels of FBW7 in TNBC (n = 185), ER-positive (n = 765), PR-positive (n = 670), and HER2-positive (n = 147) clinical samples from the TCGA RNA-seq data via BCIP. Data were analyzed by using GraphPad Prism 10. *** *P* < 0.001 by one-way ANOVA followed by Dunnett’s tests. **(e)** qPCR analysis of FBW7 expression in breast cancer cell lines. **(f)** IP and WB analyses of the ubiquitination level of 3×Flag-c-Myc mediated by FBW7α and FBW7α-ΔF-box in HEK293T cells treated with MG132 (20 μM) for 6 hours. **(g)** WB analysis of c-Myc expression levels in HEK293T-3×Flag-c-Myc cells transfected with pCDH-FBW7α-Myc(tag) and pCDH-66ATG-3×Flag. **(h)** WB analysis of 66CTG expression levels in HEK293T-66ATG-3×Flag cells transfected with pCDH-FBW7α-Myc(tag) and pCDH-3×Flag-c-Myc.

Figure. S7.

**
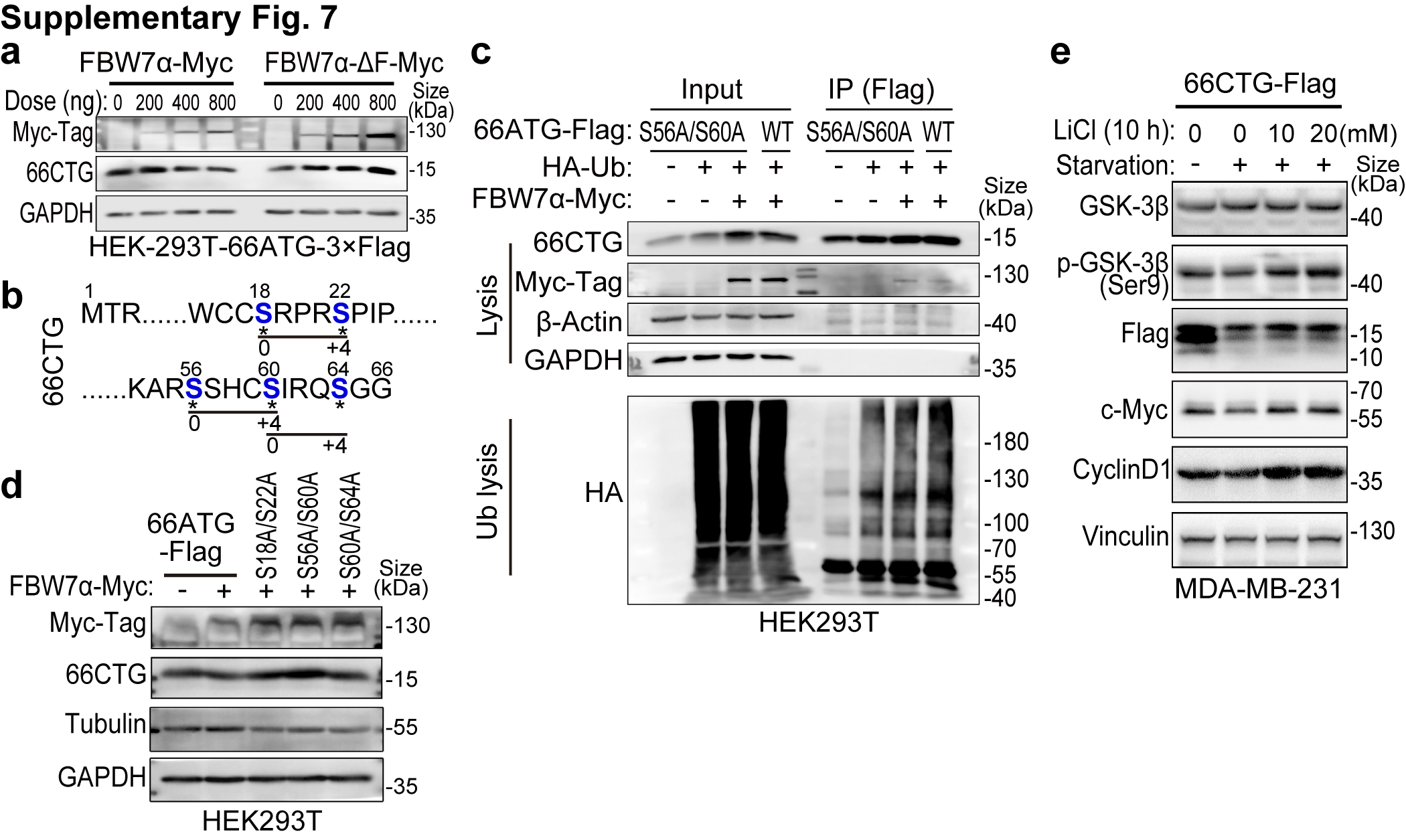
**

**Supplementary Figure 7. FBW7α mediates the ubiquitination and degradation of 66CTG via recognizing its CPD^S56/S60^ motif. (a)** WB analysis of 66CTG expression in HEK293T-66ATG-3×Flag cells transfected with varying doses of pCDH-FBW7α-Myc(tag) or pCDH-FBW7α-ΔF-Myc(tag) (0, 200, 400, and 800 ng). **(b)** Schematic diagram showing the positions of three potential CPD motifs in the amino acid sequence of 66CTG. **(c)** IP and WB analyses of the ubiquitination of 66ATG^S56A/S60A^-3×Flag and 66ATG-3×Flag mediated by FBW7α in HEK293T cells under MG132 (20 μM) treatment for 6 hours. **(d)** WB analysis of 66CTG in HEK293T cells co-transfected with pCDH-FBW7α-Myc(tag) and pCDH-66ATG-3×Flag or its three CPD motif mutants. **(e)** WB analysis of 66CTG and c-Myc protein levels in MDA-MB-231-66CTG-3×Flag cells treated with serum starvation for 36 hours, followed by LiCl (0, 10, 20 mM) treatment for 10 hours.

Table S1.

**Primers, siRNAs, and oligonucleotides used in experiments.**

| **Gene** | **Sequence (5’-3’)** | **Application** |
| --- | --- | --- |
| CDKN2B-AS1 primer | **Sense:** AGCCTCATTCTGATTCAACAGC | qPCR for CDKN2B-AS1 |
| CDKN2B-AS1 primer | **Antisense:** CAGCACACCTAACAGTGATGC | qPCR for CDKN2B-AS1 |
| 66CTG qPCR primer | **Sense:** GACACGGCCCTACCAGGAAC | qPCR for 66CTG |
| 66CTG qPCR primer | **Antisense:** CCTGATTGGCGGATAGAGCA | qPCR for 66CTG |
| c-Myc qPCR primer | **Sense:** AGTGGAAAACCAGCAGCCTC | qPCR for c-Myc |
| c-Myc qPCR primer | **Antisense:** TTCTCCTCCTCGTCGCAGTA | qPCR for c-Myc |
| Cyclin D1 qPCR primer | **Sense:** GATCAAGTGTGACCCGGACTG | qPCR for Cyclin D1 |
| Cyclin D1 qPCR primer | **Antisense:** CCTTGGGGTCCATGTTCTGC | qPCR for Cyclin D1 |
| CUL1 qPCR primer | **Sense:** AGGGTCTTGCAGCCATTGAA | qPCR for CUL1 |
| CUL1 qPCR primer | **Antisense:** TGAAGCGACCACAAGCCTTA | qPCR for CUL1 |
| SKP2 qPCR primer | **Sense:** TGCCCCAATCTTGTCCATCT | qPCR for SKP2 |
| SKP2 qPCR primer | **Antisense:** ACCATCTGGCACGATTCCAA | qPCR for SKP2 |
| β-TRCP qPCR primer | **Sense:** TTTGCAGTACAGGGACAGGC | qPCR for β-TRCP |
| β-TRCP qPCR primer | **Antisense:** CAGCCACAAGATCCCACACT | qPCR for β-TRCP |
| FBW7 qPCR primer | **Sense:** AGCTGGAGTGGACCAGAGAA | qPCR for FBW7 |
| FBW7 qPCR primer | **Antisense:** AGCAGGTCTTTGGGTTCCAG | qPCR for FBW7 |
| m/h18Sreg3 primer | **Sense:** CTCAACACGGGAAACCTCAC | qPCR for 18S |
| m/h18Sreg3 primer | **Antisense:** CGCTCCACCAACTAAGAACG | qPCR for 18S |
| si66CTG#1 | **Sense:** GUCUUCCAUUCUUCAAACUdTdT | siRNA targeting 66CTG |
| si66CTG#1 | **Antisense:** AGUUUGAAGAAUGGAAGACdTdT | siRNA targeting 66CTG |
| si66CTG#5 | **Sense:** CAGCCGCGCUCCCGCGGAUdTdT | siRNA targeting 66CTG |
| si66CTG#5 | **Antisense:** AUCCGCGGGAGCGCGGCUGdTdT | siRNA targeting 66CTG |
| sic-Myc#1 | **Sense:** GGAACUAUGACCUCGACUAdTdT | siRNA targeting c-Myc |
| sic-Myc#1 | **Antisense:** UAGUCGAGGUCAUAGUUCCdTdT | siRNA targeting c-Myc |
| sic-Myc#2 | **Sense:** CCAAGGUAGUUAUCCUUAAdTdT | siRNA targeting c-Myc |
| sic-Myc#2 | **Antisense:** UUAAGGAUAACUACCUUGGdTdT | siRNA targeting c-Myc |
| sic-Myc#3 | **Sense:** CCUGAGACAGAUCAGCAACAAdTdT | siRNA targeting c-Myc |
| sic-Myc#3 | **Antisense:** UUGUUGCUGAUCUGUCUCAGGdTdT | siRNA targeting c-Myc |
| siCUL1#1 | **Sense:** CAUCAAUGUGCCAAUGAAAdTdT | siRNA targeting CUL1 |
| siCUL1#1 | **Antisense:** UUUCAUUGGCACAUUGAUGdTdT | siRNA targeting CUL1 |
| siCUL1#2 | **Sense:** CAUCGAGGAAGACCGCAAAdTdT | siRNA targeting CUL1 |
| siCUL1#2 | **Antisense:** UUUGCGGUCUUCCUCGAUGdTdT | siRNA targeting CUL1 |
| siSKP2#1 | **Sense:** AAGUGAUAGUGUCAUGCUAAAdTdT | siRNA targeting SKP2 |
| siSKP2#1 | **Antisense:** UUUAGCAUGACACUAUCACUUdTdT | siRNA targeting SKP2 |
| siSKP2#2 | **Sense:** AACACCUAUCACUCAGUCGGUdTdT | siRNA targeting SKP2 |
| siSKP2#2 | **Antisense:** ACCGACUGAGUGAUAGGUGUUdTdT | siRNA targeting SKP2 |
| siβ-TRCP#1 | **Sense:** GGAGUUUACUGUUUACAGUdTdT | siRNA targeting β-TRCP |
| siβ-TRCP#1 | **Antisense:** ACUGUAAACAGUAAACUCCdTdT | siRNA targeting β-TRCP |
| siβ-TRCP#2 | **Sense:** GCAGUUCUGCACUUGCGUUdTdT | siRNA targeting β-TRCP |
| siβ-TRCP#2 | **Antisense:** AACGCAAGUGCAGAACUGCdTdT | siRNA targeting β-TRCP |
| siFBW7#1 | **Sense:** AAGAAACCAUGCAAAGUCUCAdTdT | siRNA targeting FBW7 |
| siFBW7#1 | **Antisense:** UGAGACUUUGCAUGGUUUCUUdTdT | siRNA targeting FBW7 |
| siFBW7#2 | **Sense:** AACCAACACAAGUAAAACAUAdTdT | siRNA targeting FBW7 |
| siFBW7#2 | **Antisense:** UAUGUUUUACUUGUGUUGGUUdTdT | siRNA targeting FBW7 |
| siGSK-3β#1 | **Sense:** GUAUUGCAGGACAAGAGAUdTdT | siRNA targeting GSK-3β |
| siGSK-3β#1 | **Antisense:** AUCUCUUGUCCUGCAAUACdTdT | siRNA targeting GSK-3β |
| siGSK-3β#2 | **Sense:** GCUAGAUCACUGUAACAUAdTdT | siRNA targeting GSK-3β |
| siGSK-3β#2 | **Antisense:** UAUGUUACAGUGAUCUAGCdTdT | siRNA targeting GSK-3β |
|  |  | siRNA targeting GSK-3β |
| sg66CTG#T4 | **Sense:** CACCGACATTCAGCCTCCTGATTGG | sgRNA at C-terminal |
| sg66CTG#T4 | **Antisense:** AAACCCAATCAGGAGGCTGAATGTC | sgRNA at C-terminal |
| sg66CTG#T6 | **Sense:** CACCGAATCTAGAAGAGGAGCGGAG | sgRNA at C-terminal |
| sg66CTG#T6 | **Antisense:** AAACCTCCGCTCCTCTTCTAGATTC | sgRNA at C-terminal |
| 66CTG ssODN#1 | **Sense:** CTTCCATTCTTCAAACTAGATTATTTAAAAATGAAAAAGGAAGAAAGGAAAGCGAGGTCATCTCATTGCTCTATCCGCCAATCAGGAGGCtacccgtatgatgttccggattacgctggctatccctacgacgtgcccgactatgccgggtacccctatgacgtcccagactacgcaTGAATGTCAGTTTTGAACTAAAAGCCGCTCCGCTCCTCTTCTAGATTTGGAAAACAAGCGAAATTAAACTAAACCGCTGCACGCCTCTGA | ssODN with 3×HA for HDR at C-terminal |
| 66CTG ssODN#1 | **Antisense:** TCAGAGGCGTGCAGCGGTTTAGTTTAATTTCGCTTGTTTTCCAAATCTAGAAGAGGAGCGGAGCGGCTTTTAGTTCAAAACTGACATTCATGCGTAGTCTGGGACGTCATAGGGGTACCCGGCATAGTCGGGCACGTCGTAGGGATAGCCAGCGTAATCCGGAACATCATACGGGTAGCCTCCTGATTGGCGGATAGAGCAATGAGATGACCTCGCTTTCCTTTCTTCCTTTTTCATTTTTAAATAATCTAGTTTGAAGAATGGAAG | ssODN with 3×HA for HDR at C-terminal |
| 66CTG-3×HA Primer | **Sense:** GACACGGCCCTACCAGGAAC | qPCR or PCR for 66CTG-3×HA |
| 66CTG-3×HA Primer | **Antisense:** ACATCATACGGGTAGCCTCCT | qPCR or PCR for 66CTG-3×HA |
| sg66CTG#T13 | **Sense:** CACCGCGCGGGAGCGCGGCTGTTCC | sgRNA at N-terminal |
| sg66CTG#T13 | **Antisense:** AAACGGAACAGCCGCGCTCCCGCGC | sgRNA at N-terminal |
| 66CTG ssODN#2 | **Sense:** CCTCAGCGCGGGCGCCCCGCGGTGACGGCCCAGGGGCCGGACGCCTGGAACGCAACTCCAGGCAGCTCGCCCCCTAGCTACATCCGTCACCTGtacccgtatgatgttccggattacgctggctatccctacgacgtgcccgactatgccgggtacccctatgacgtcccagactacgcaACACGGCCCTACCAGGAACAGCCGCGCTCCCGCGGATTCTGGTGCTGCTCGCGTCCCCGCTCCCCTATTCCCCTTATTTTATTCCTG | ssODN with CTG-3×HA for HDR at N-terminal |
| 66CTG ssODN#2 | **Antisense:** CAGGAATAAAATAAGGGGAATAGGGGAGCGGGGACGCGAGCAGCACCAGAATCCGCGGGAGCGCGGCTGTTCCTGGTAGGGCCGTGTTGCGTAGTCTGGGACGTCATAGGGGTACCCGGCATAGTCGGGCACGTCGTAGGGATAGCCAGCGTAATCCGGAACATCATACGGGTACAGGTGACGGATGTAGCTAGGGGGCGAGCTGCCTGGAGTTGCGTTCCAGGCGTCCGGCCCCTGGGCCGTCACCGCGGGGCGCCCGCGCTGAGG | ssODN with CTG-3×HA for HDR at N-terminal |
| 66CTG ssODN#3 | **Sense:** CCTCAGCGCGGGCGCCCCGCGGTGACGGCCCAGGGGCCGGACGCCTGGAACGCAACTCCAGGCAGCTCGCCCCCTAGCTACATCCGTCACCCGtacccgtatgatgttccggattacgctggctatccctacgacgtgcccgactatgccgggtacccctatgacgtcccagactacgcaACACGGCCCTACCAGGAACAGCCGCGCTCCCGCGGATTCTGGTGCTGCTCGCGTCCCCGCTCCCCTATTCCCCTTATTTTATTCCTG | ssODN with CCG-3×HA for HDR at N-terminal |
| 66CTG ssODN#3 | **Antisense:** CAGGAATAAAATAAGGGGAATAGGGGAGCGGGGACGCGAGCAGCACCAGAATCCGCGGGAGCGCGGCTGTTCCTGGTAGGGCCGTGTTGCGTAGTCTGGGACGTCATAGGGGTACCCGGCATAGTCGGGCACGTCGTAGGGATAGCCAGCGTAATCCGGAACATCATACGGGTACGGGTGACGGATGTAGCTAGGGGGCGAGCTGCCTGGAGTTGCGTTCCAGGCGTCCGGCCCCTGGGCCGTCACCGCGGGGCGCCCGCGCTGAGG | ssODN with CCG-3×HA for HDR at N-terminal |
| 3×HA-66CTG/CCG P1 | **Sense:** CGGATTACGCTGGCTATCCC | qPCR or PCR for 3×HA-66CTG/CCG |
| 3×HA-66CTG/CCG P1 | **Antisense:** TAAGGGGAATAGGGGAGCGG | qPCR or PCR for 3×HA-66CTG/CCG |
| 3×HA-66CTG/CCG P2 | **Sense:** GACGCCTGGAACGCAACTC | qPCR or PCR for 3×HA-66CTG/CCG |
| 3×HA-66CTG/CCG P2 | **Antisense:** GTCGTAGGGATAGCCAGCGT | qPCR or PCR for 3×HA-66CTG/CCG |
| 3×HA-66CTG/CCG P3 | **Sense:** GCCTGGAACGCAACTCCA | Primer for sequencing |
| 3×HA-66CTG/CCG P3 | **Antisense:** CTGCTGTTGAATCAGAATGAGG | Primer for sequencing |
| 66CTG Primer | **Sense:** CTGACACGGCCCTACCAGGAA | PCR amplify 66CTG from cDNA |
| 66CTG Primer | **Antisense:** TCAGCCTCCTGATTGGCGGAT | PCR amplify 66CTG from cDNA |
| 66CTG-3×Flag Primer | **Sense:** cttggtaccgagctcggatccGCCGCCACCCTGACACGG | PCR amplify 66CTG-3×Flag for pcDNA3.1(+) or pCDH-CMV-MCS-Puro (notag) with BamHI |
| 66ATG-3×Flag Primer | **Sense:** cttggtaccgagctcggatccGCCGCCACCATGACACGG | PCR amplify 66ATG-3×Flag for pcDNA3.1(+) or pCDH-CMV-MCS-Puro (notag) with BamHI |
| 66CCG-3×Flag Primer | **Sense:** cttggtaccgagctcggatccGCCGCCACCCCGACACGG | PCR amplify 66CCG-3×Flag for pcDNA3.1(+) or pCDH-CMV-MCS-Puro (notag) with BamHI |
| 66C/A/CTG-3×Flag Primer | **Antisense:** atcctcgagtctgcagaattcTCACTTGTCATCGTCATCCTTGTAATCGATGTCATGATCTTTATAATCACCGTCATGGTCTTTGTAGTCGCCTCCTGATTGGCGGAT | PCR amplify 66CTG/ATG/CCG-3×Flag for pcDNA3.1(+) or pCDH-CMV-MCS-Puro (notag) with EcoRI |
| c-Myc Primer F1 | **Sense:** gatgacaagtctagagaattcGATTTTTTTCGGGTAGTGG | PCR amplify c-Myc for pCDH-CMV-3×Flag-MCS-puro with EcoRI |
| c-Myc Primer R1 | **Antisense:** gagcccgggaatattggatccTTACGCACAAGAGTTCCGTAGC | PCR amplify c-Myc for pCDH-CMV-3×Flag-MCS-puro with BamHI |
| c-Myc Primer F2 | **Sense:** cttggtaccgagctcggatccGCCACCATGGATTTTTTTCGGGTAGTGG | PCR amplify c-Myc (notag) or c-Myc-3×Flag for pCDH-CMV-MCS-puro (notag) with BamHI |
| c-Myc Primer R2 | **Antisense:** atcctcgagtctgcagaattcTTACGCACAAGAGTTCCGTAGC | PCR amplify c-Myc (notag) for pCDH-CMV-MCS-puro (notag) with EcoRI |
| c-Myc-3×Flag Primer R | **Antisense:** atcctcgagtctgcagaattcTTACTTGTCATCGTCATCCTTGTAATCGATGTCATGATCTTTATAATCACCGTCATGGTCTTTGTAGTCCGCACAAGAGTTCCGTAGC | PCR amplify c-Myc-3×Flag for pCDH-CMV-MCS-puro (notag) with EcoRI |
| sh66CTG#1 | **Sense:** GATCCGTCTTCCATTCTTCAAACTCTTCCTGTCAGAAGTTTGAAGAATGGAAGACTTTTTG | shRNA targeting 66CTG |
| sh66CTG#1 | **Antisense:** AATTCAAAAAGTCTTCCATTCTTCAAACTTCTGACAGGAAGAGTTTGAAGAATGGAAGACG | shRNA targeting 66CTG |
| sh66CTG#5 | **Sense:** GATCCCAGCCGCGCTCCCGCGGATCTTCCTGTCAGAATCCGCGGGAGCGCGGCTGTTTTTG | shRNA targeting 66CTG |
| sh66CTG#5 | **Antisense:** AATTCAAAAACAGCCGCGCTCCCGCGGATTCTGACAGGAAGATCCGCGGGAGCGCGGCTGG | shRNA targeting 66CTG |
| siCDKN2B-AS1#1 | **Sense:** CACACAUCAAAGGAGAAUUdTdT | siRNA targeting CDKN2B-AS1 |
|  | **Antisense:** AAUUCUCCUUUGAUGUGUGdTdT | siRNA targeting CDKN2B-AS1 |
| siCDKN2B-AS1#2 | **Sense:** GAAAACCGGGGAGAUCUAUdTdT | siRNA targeting CDKN2B-AS1 |
|  | **Antisense:** AUAGAUCUCCCCGGUUUUCdTdT | siRNA targeting CDKN2B-AS1 |
| siCDKN2B-AS1#3 | **Sense:** CUCCAAAGAAACCAUCAGAdTdT | siRNA targeting CDKN2B-AS1 |
|  | **Antisense:** UCUGAUGGUUUCUUUGGAGdTdT | siRNA targeting CDKN2B-AS1 |

Table S2.

**Antibodies and reagents used in Western blot.**

| **Antibody name** | **Source** | **Identifier** |
| --- | --- | --- |
| Rabbit polyAb anti-66CTG | GL Biochem (Shanghai) Ltd | Product ID: AB012233  Animal ID: RB4551 |
| Mouse mAb anti-HA-tag  Clone (AMC0503) | ABclonal | Cat#AE008  RRID: AB_2770404 |
| Mouse mAb anti-HA-tag  Clone (T80) | Affinity Biosciences | Cat#T0008  RRID: AB_2839415 |
| Mouse mAb anti-HA-tag | Abways (Shanghai) | Cat#AB0004  RRID: N/A |
| Mouse mAb anti-alpha-Tubulin  Clone (B-5-1-2) | Sigma-Aldrich | Cat#T5168  RRID: AB_477579 |
| Mouse mAb anti-Flag (DDDDK-tag)  Clone (FLA-1) | MBL International | Cat#M185-3L  RRID: AB_11123930 |
| Rabbit polyAb anti-AKT | Proteintech | Cat#10176-2-AP  RRID: AB_2224574 |
| Rabbit mAb anti-phospho-AKT (Thr308)  Clone (D25E6) | Cell Signal Technology | Cat#13038  RRID: AB_2629447 |
| Rabbit mAb anti-CyclinD1  Clone (ARC0300) | ABclonal | Cat#A19038  RRID: AB_2862530 |
| Rabbit mAb anti-CDK4  Clone (D9G3E) | Cell Signal Technology | Cat#12790  RRID: AB_2631166 |
| Mouse mAb anti-CyclinE  Clone (HE12) | Santa Cruz Biotechnology | Cat#sc-247  RRID: AB_627357 |
| Mouse mAb anti-CDK2  Clone (D-12) | Santa Cruz Biotechnology | Cat#sc-6248  RRID: AB_627238 |
| Mouse mAb anti-Vinculin  Clone (hVIN-1) | Sigma-Aldrich | Cat#V9264  RRID: AB_10603627 |
| Rabbit mAb anti-c-Myc  Clone (YA497) | MedChemExpress | Cat#HY-P80626 |
| Rabbit mAb anti-p27 Kip1  Clone (D69C12) | Cell Signal Technology | Cat#3686  RRID: AB_2077850 |
| Rabbit polyAb anti-CUL1 | Proteintech | Cat#12895-1-AP  RRID: AB_2086291 |
| Rabbit mAb anti-Myc-tag  Clone (ARC5004-12) | ABclonal | Cat#AE070  RRID: AB_2863795 |
| Mouse mAb anti-GAPDH  Clone (1E6D9) | Proteintech | Cat#60004-1-Ig  RRID: AB_2107436 |
| Rabbit mAb anti-Lamin B1  Clone (D9V6H) | Cell Signal Technology | Cat#13435  RRID: AB_2737428 |
| Mouse mAb anti-β-Actin  Clone (AC-15) | Sigma-Aldrich | Cat#A5441  RRID: AB_476744 |
| Rabbit polyAb anti-GSK-3β | Proteintech | Cat#22104-1-AP  RRID: AB_2878997 |
| Rabbit mAb anti-phospho-GSK-3β (Ser9)  Clone (D85E12) | Cell Signal Technology | Cat#5558  RRID: AB_10013750 |
| Multi-rAb™ HRP-Goat Anti-Mouse Recombinant Secondary Antibody (H+L) | Proteintech | Cat#RGAM001  RRID: AB_3068333 |
| Multi-rAb™ HRP-Goat Anti-Rabbit Recombinant Secondary Antibody (H+L) | Proteintech | Cat#RGAR001  RRID: AB_3073505 |
| Anti-Flag magnetic beads | MedChemExpress | Cat#HY-K0207 |
| Anti-c-Myc magnetic beads | MedChemExpress | Cat: HY-K0206 |

Table S3.

**Results from ORFfinder related to Figure 1b.**

Table S4.

**Results from SmProt related to Figure 1b.**

Dataset 1. (separate file)

The clinicopathological data of 8 breast cancer tissue samples related to Supplementary Figure 1a-b.

Dataset 2. (separate file)

The clinicopathological data of 89 triple-negative breast cancer tissue samples related to Figure 4g-i, and Supplementary Figure 4l.

Dataset 3. (separate file)

The source data of RNA-seq for siNC vs. si66CTG#1 related to Figure 3h and Supplementary Figure 3c.

Dataset 4. (separate file)

The source data of RNA-seq for siNC vs. si66CTG#5 related to Figure 3h and Supplementary Figure 3c.

Dataset 5. (separate file)

The source data of RNA-seq for siNC vs. sic-Myc#1 related to Figure 3h and Supplementary Figure 3c.

Dataset 6. (separate file)

The source data of RNA-seq for siNC vs. sic-Myc#2 related to Figure 3h and Supplementary Figure 3c.

Dataset 7. (separate file)

The source data of RNA-seq for siNC vs. sic-Myc#3 related to Figure 3h and Supplementary Figure 3c.

Dataset 8. (separate file)

The original films of Western blot in this study.

Dataset 9. (separate file)

The original images of the MDA-MB-231-Luc xenograft related to Figure 4a.

Dataset 10. (separate file)

The original images of immunohistochemistry related to Figure 4g and Supplementary Fig. 4l.
