# Supplementary material for: A novel peptide 66CTG stabilizes Myc proto-oncogene protein to promote triple-negative breast cancer growth
Source: Signal Transduct Target Ther. 2025 Jul 9;10:217. doi: 10.1038/s41392-025-02298-5 (PMC12238259; doi:10.1038/s41392-025-02298-5)

Supplementary Dataset 8_Original data of western blot

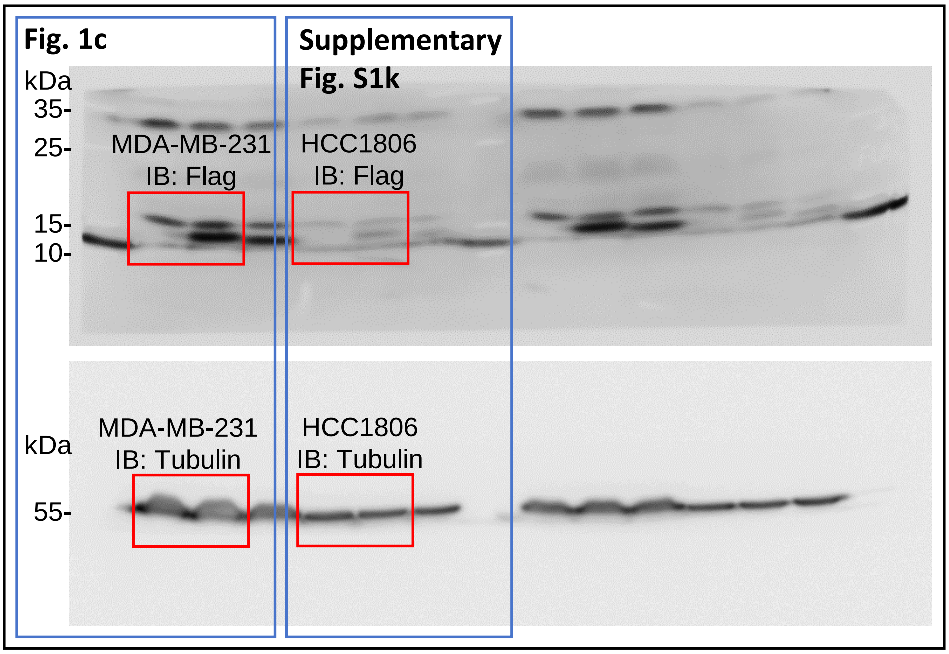

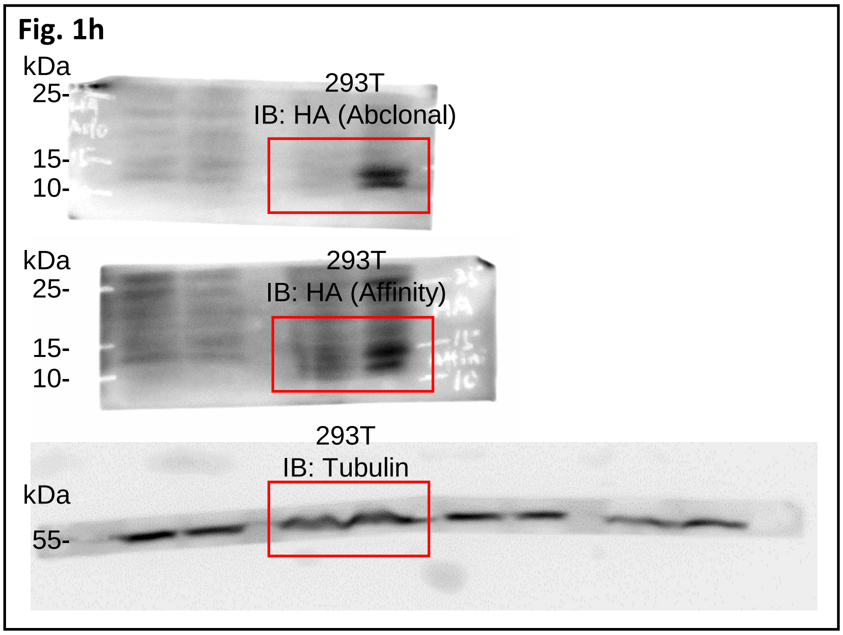

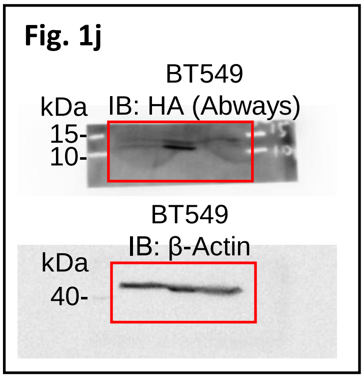

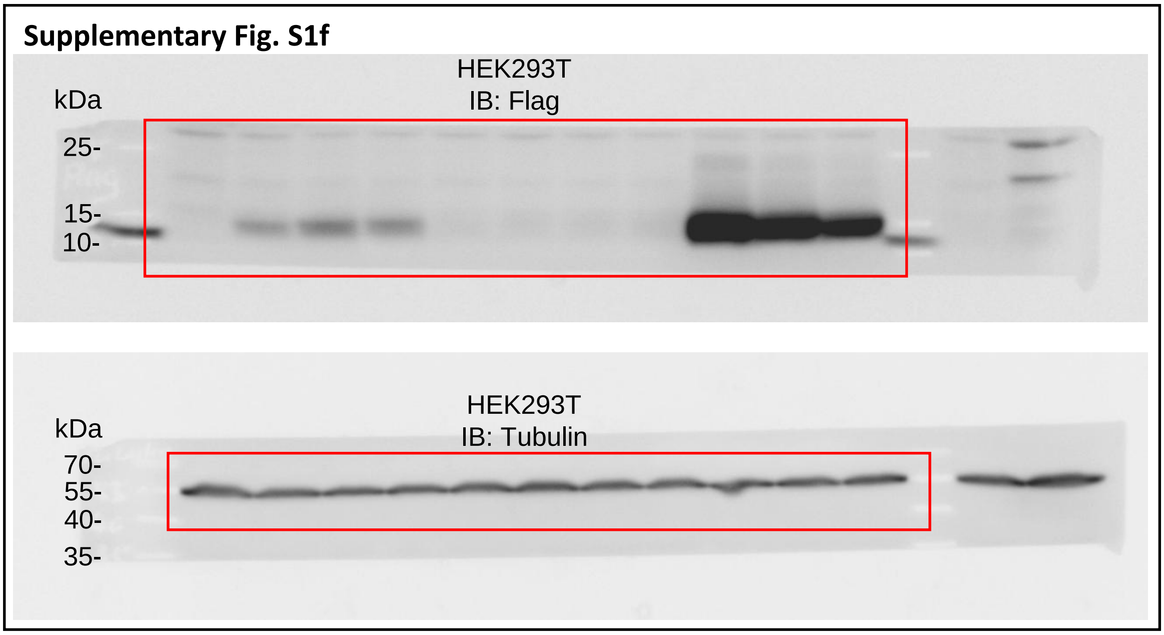

Fig. 2d

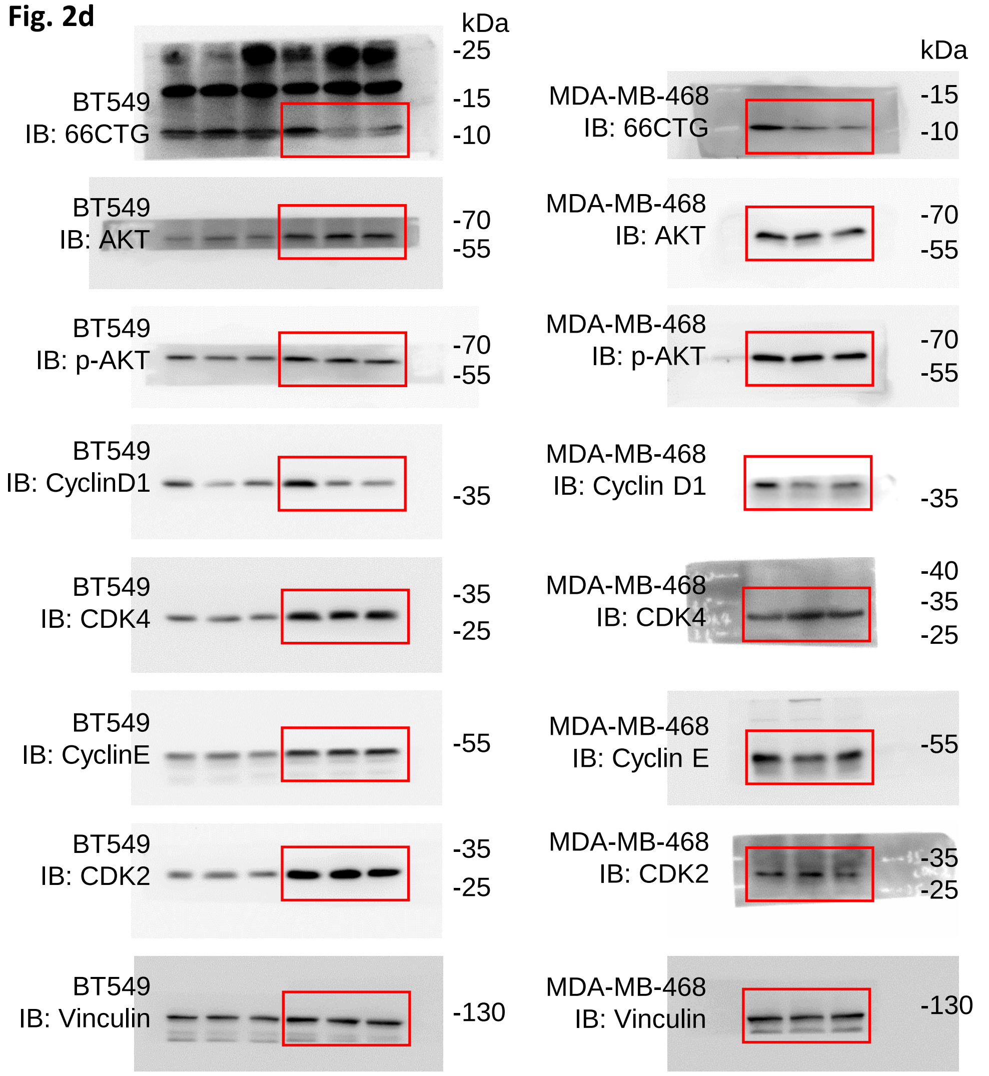
kDa
BT549
IB: 66CTG
-25
-15
-10
MDA-MB-468
IB: 66CTG
kDa
-15
-10
BT549
IB: AKT
-70
-55
MDA-MB-468
IB: AKT
-70
-55
BT549
IB: p-AKT
-70
-55
MDA-MB-468
IB: p-AKT
-70
-55
BT549
IB: CyclinD1
-35
MDA-MB-468
IB: Cyclin D1
-35
BT549
IB: CDK4
-35
-25
MDA-MB-468
IB: CDK4
-40
-35
-25
BT549
IB: CyclinE
-55
MDA-MB-468
IB: Cyclin E
-55
BT549
IB: CDK2
-35
-25
MDA-MB-468
IB: CDK2
-35
-25
BT549
IB: Vinculin
-130
MDA-MB-468
IB: Vinculin
-130

Fig. 2e

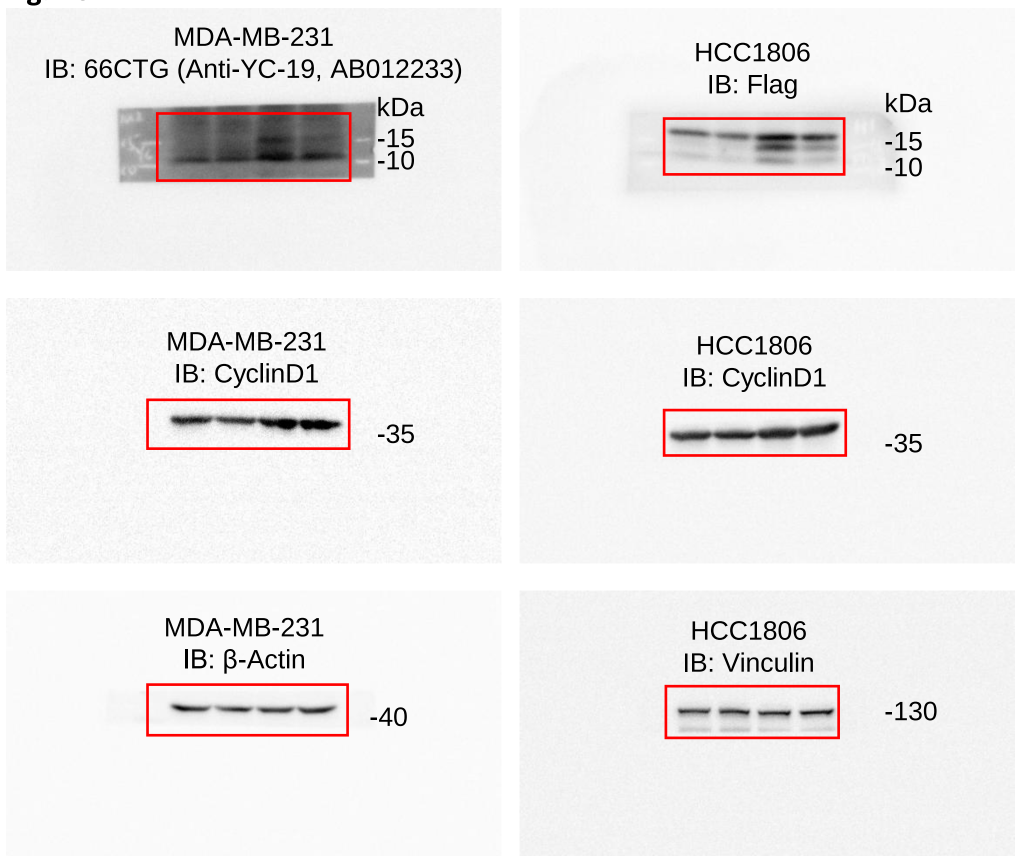
MDA-MB-231
IB: 66CTG (Anti-YC-19, AB012233)
kDa
-15
-10
HCC1806
IB: Flag
kDa
-15
-10
MDA-MB-231
IB: CyclinD1
-35
HCC1806
IB: CyclinD1
-35
MDA-MB-231
IB: β-Actin
-40
HCC1806
IB: Vinculin
-130

Fig. 3b

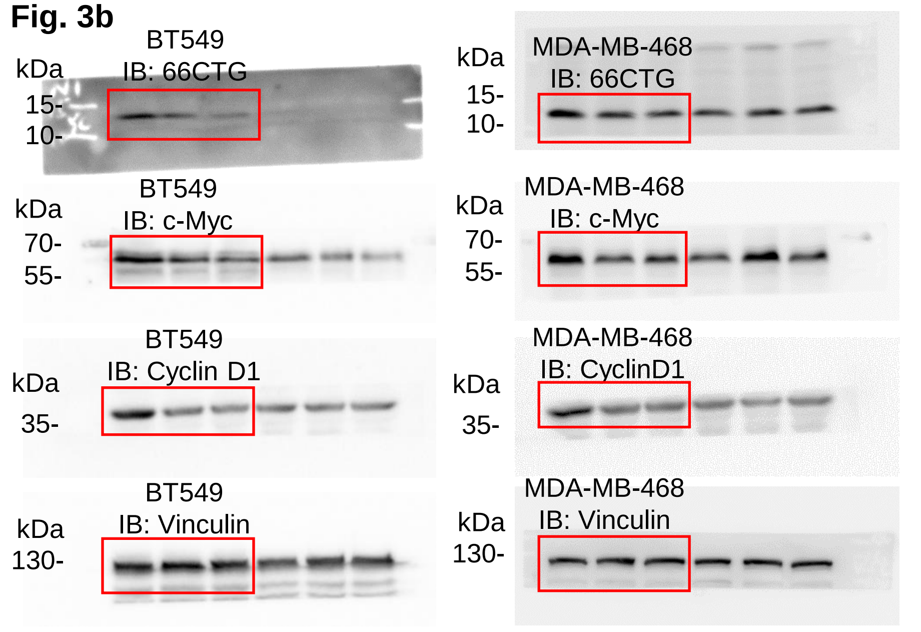

Fig. 3d

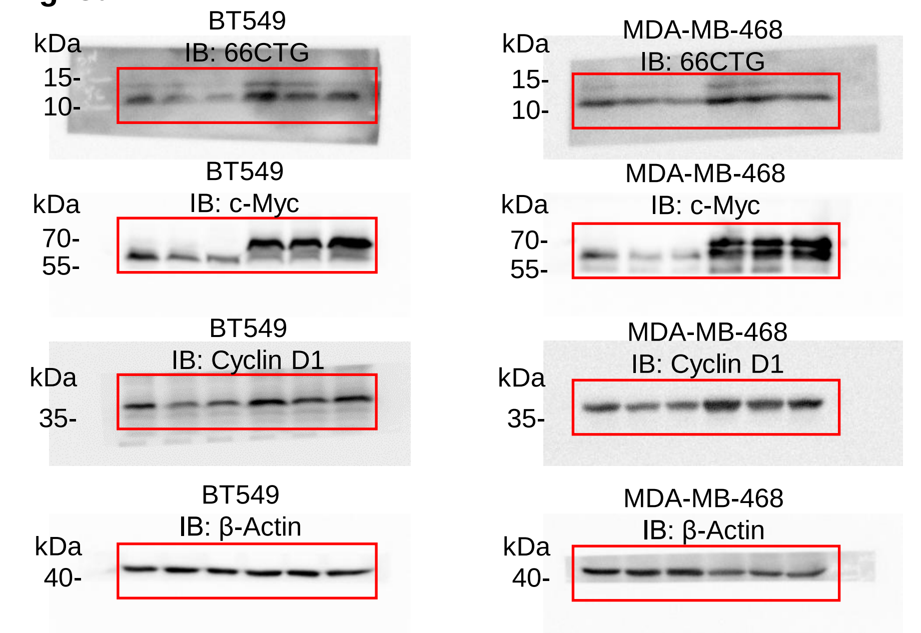

Fig. 3f

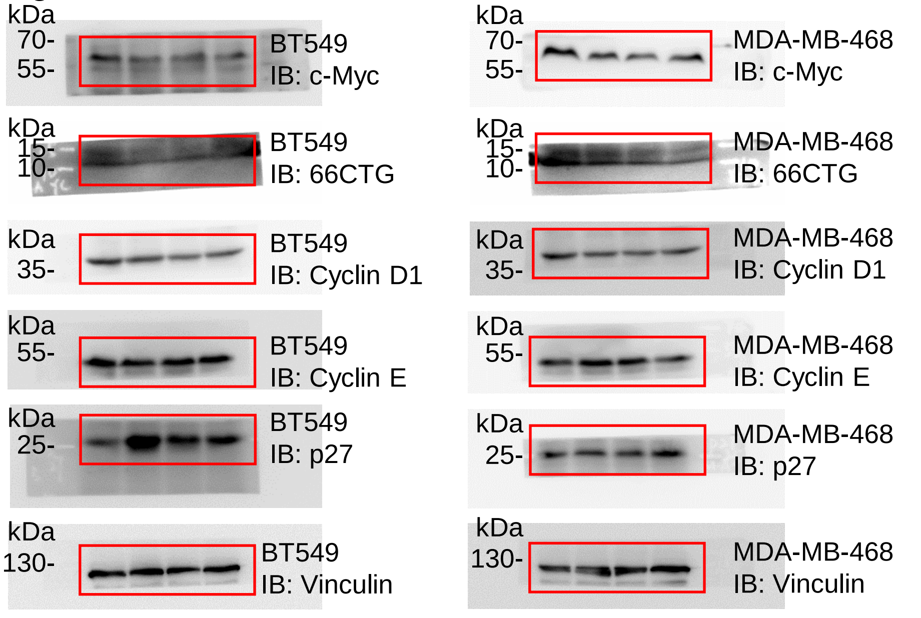

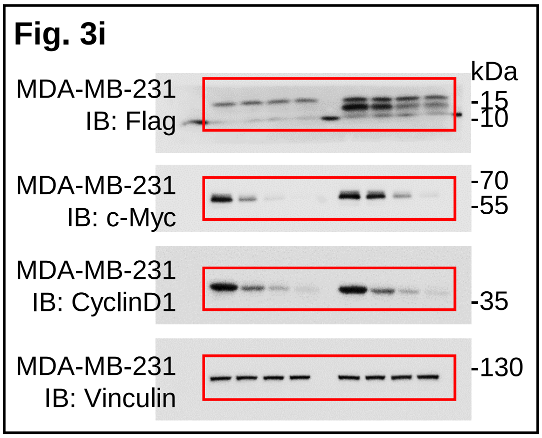
Fig. 3i
MDA-MB-231
IB: Flag
kDa
-15
-10
MDA-MB-231
IB: c-Myc
-70
-55
MDA-MB-231
IB: CyclinD1
-35
MDA-MB-231
IB: Vinculin
-130

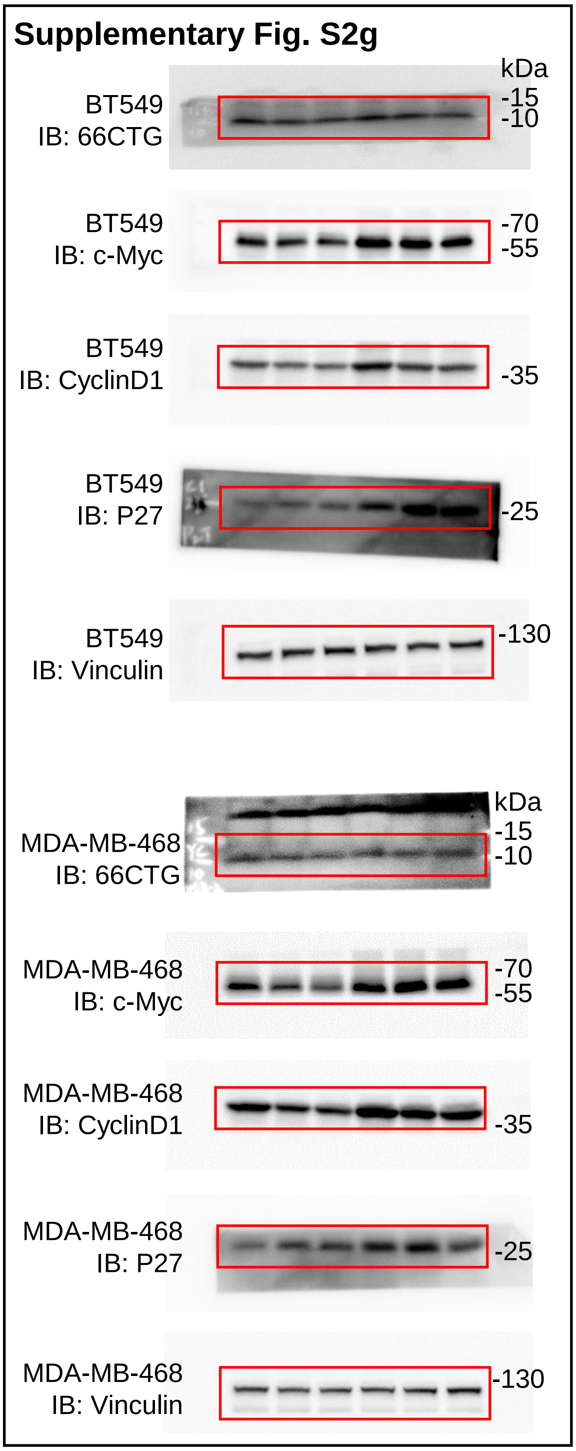
Supplementary Fig. S2g
BT549
IB: 66CTG
kDa
-15
-10
BT549
IB: c-Myc
-70
-55
BT549
IB: CyclinD1
-35
BT549
IB: P27
-25
BT549
IB: Vinculin
-130
MDA-MB-468
IB: 66CTG
kDa
-15
-10
MDA-MB-468
IB: c-Myc
-70
-55
MDA-MB-468
IB: CyclinD1
-35
MDA-MB-468
IB: P27
-25
MDA-MB-468
IB: Vinculin
-130

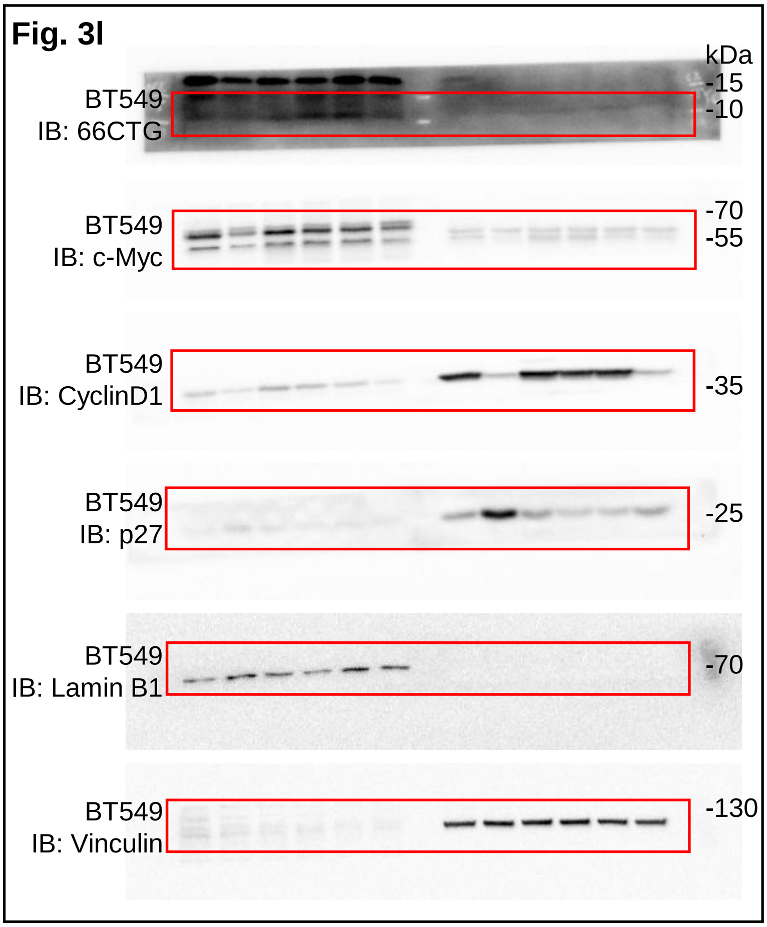
Fig. 3l
BT549
IB: 66CTG
kDa
-15
-10
BT549
IB: c-Myc
-70
-55
BT549
IB: CyclinD1
-35
BT549
IB: p27
-25
BT549
IB: Lamin B1
-70
BT549
IB: Vinculin
-130

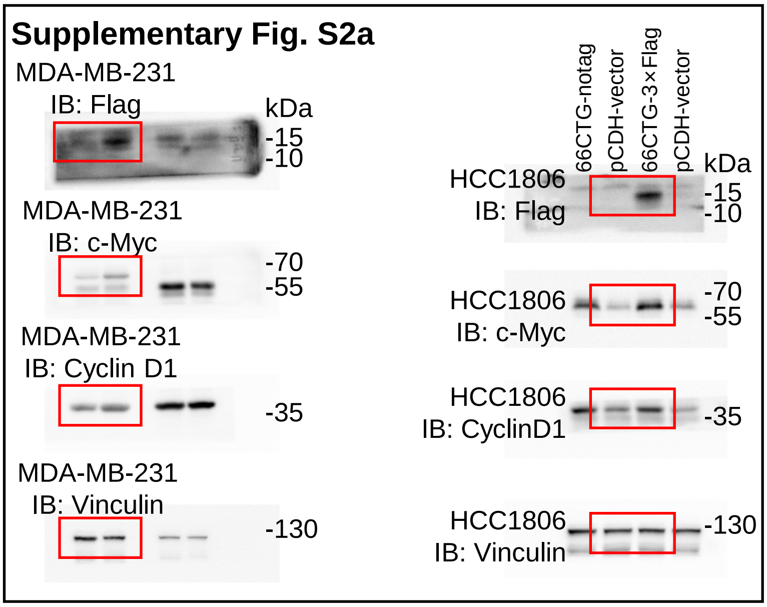
Supplementary Fig. S2a
MDA-MB-231
IB: Flag
kDa
-15
-10
MDA-MB-231
IB: c-Myc
-70
-55
MDA-MB-231
IB: Cyclin D1
-35
MDA-MB-231
IB: Vinculin
-130
66CTG-notag
pCDH-vector
66CTG-3×Flag
pCDH-vector
HCC1806
IB: Flag
kDa
-15
-10
HCC1806
IB: c-Myc
-70
-55
HCC1806
IB: CyclinD1
-35
HCC1806
IB: Vinculin
-130

Supplementary Fig. S2h

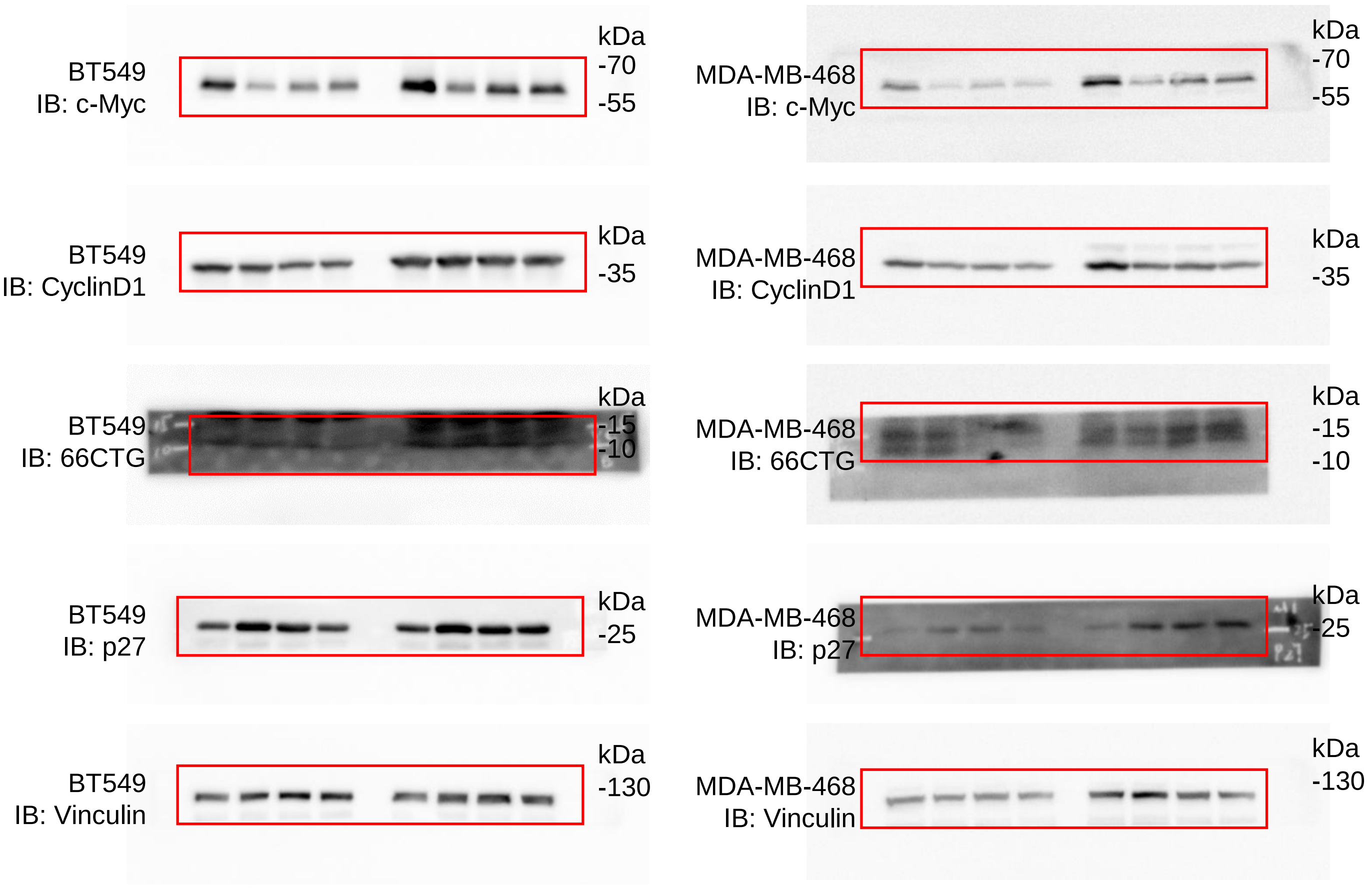

Supplementary Fig. S2i

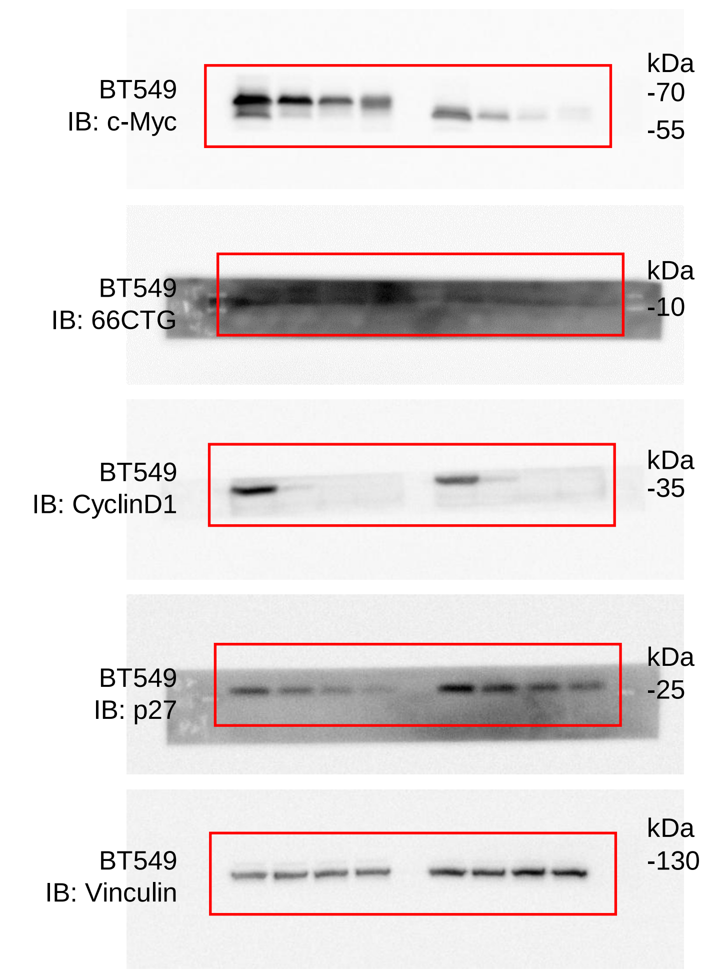

Supplementary Fig. S4a

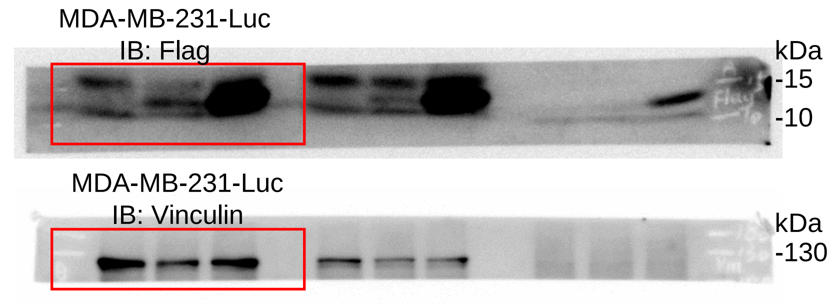

Supplementary Fig. S4e

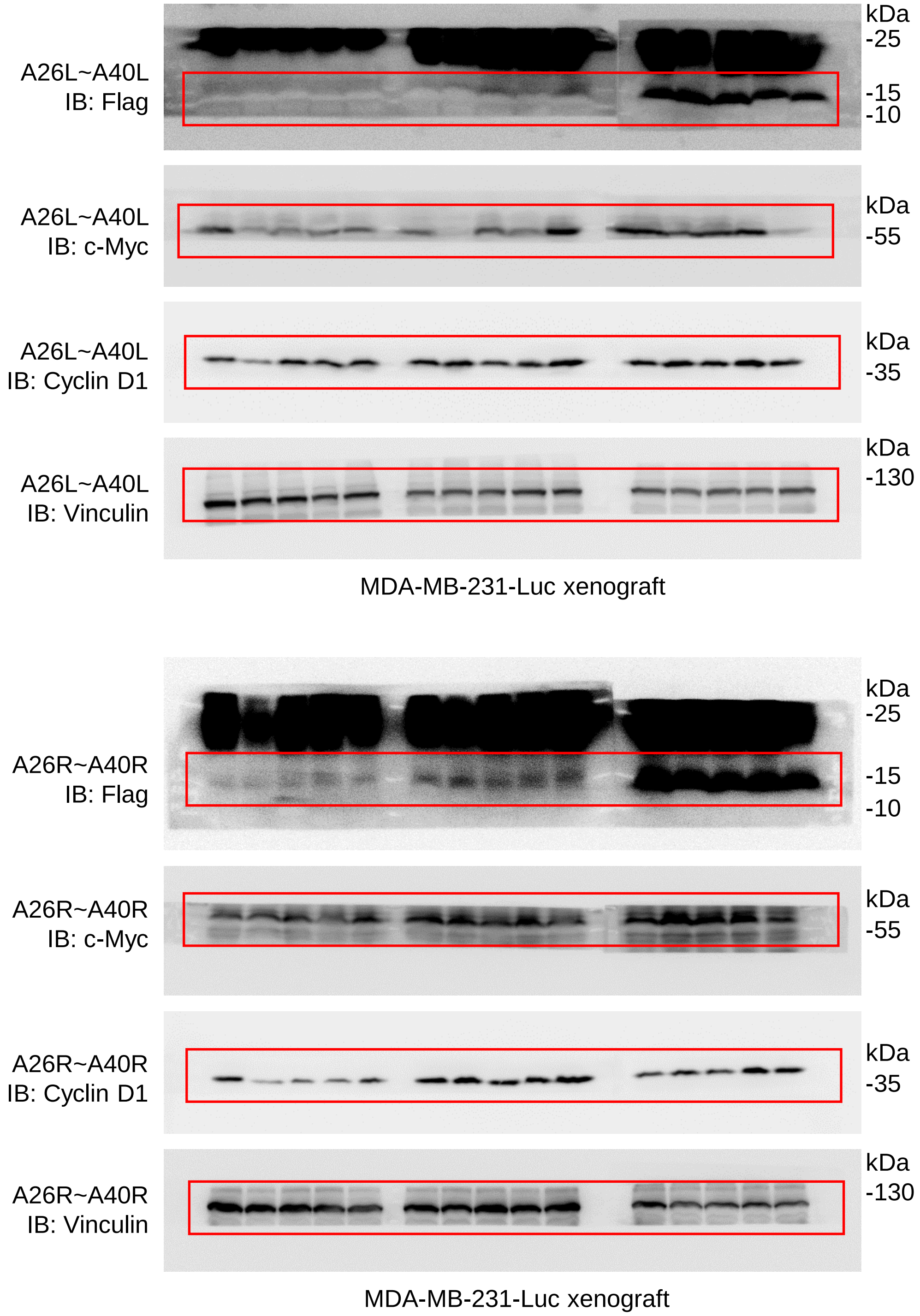

Supplementary Fig. S4f

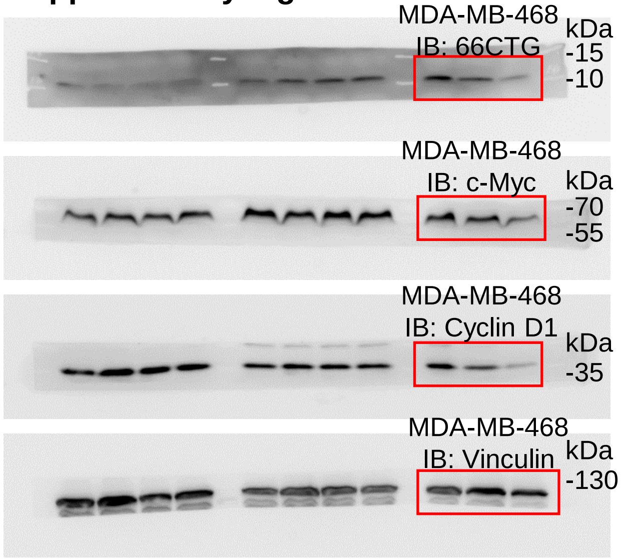

Supplementary Fig. S4j

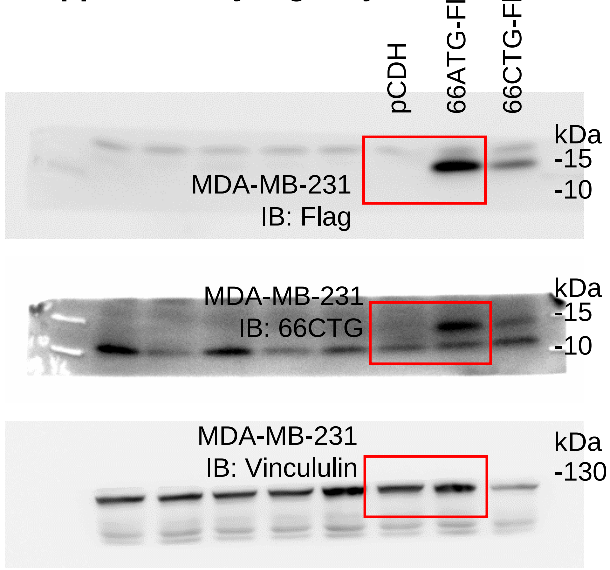

Supplementary Fig. S4h

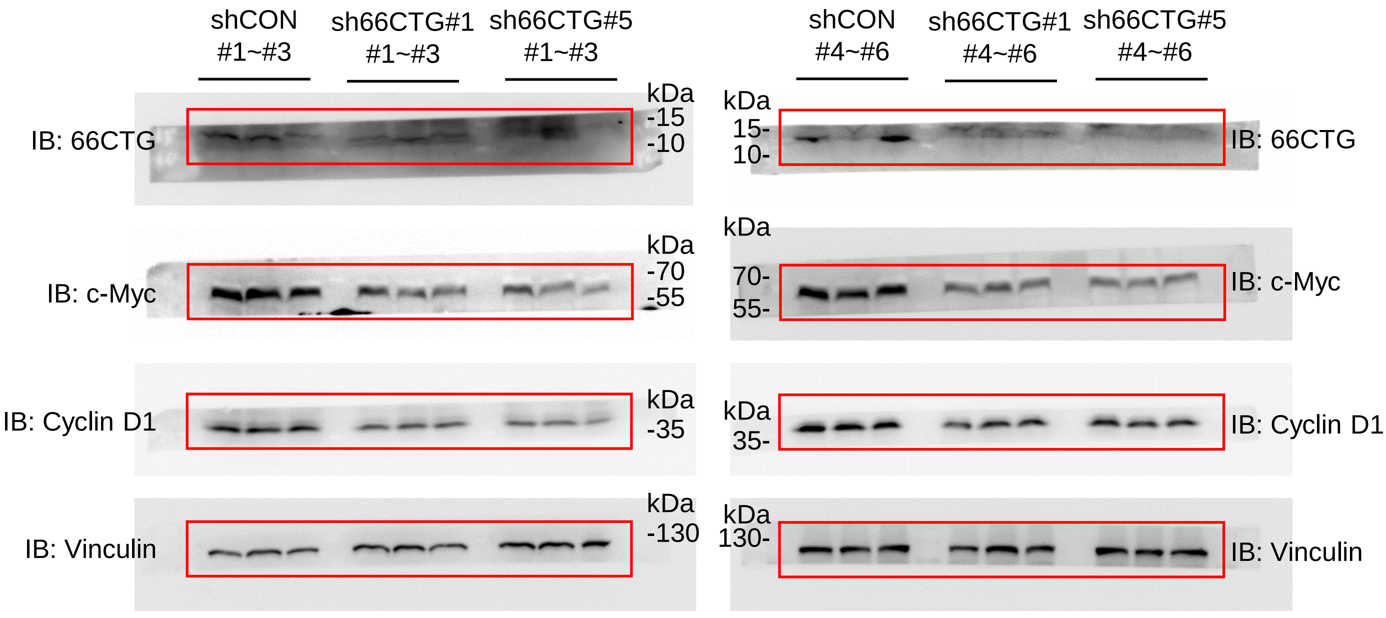

MDA-MB-468 xenograft

Fig. 5b

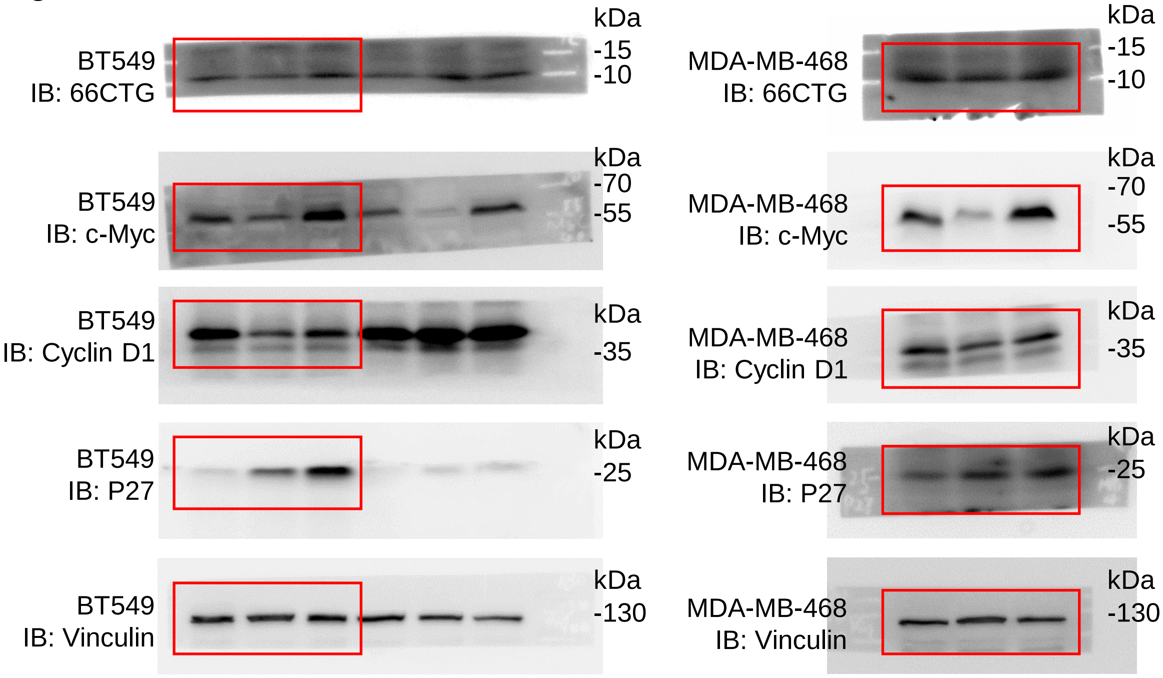
BT549
IB: 66CTG
kDa
-15
-10
MDA-MB-468
IB: 66CTG
kDa
-15
-10
BT549
IB: c-Myc
kDa
-70
-55
MDA-MB-468
IB: c-Myc
kDa
-70
-55
BT549
IB: Cyclin D1
kDa
-35
MDA-MB-468
IB: Cyclin D1
kDa
-35
BT549
IB: P27
kDa
-25
MDA-MB-468
IB: P27
kDa
-25
BT549
IB: Vinculin
kDa
-130
MDA-MB-468
IB: Vinculin
kDa
-130

Fig. 5d

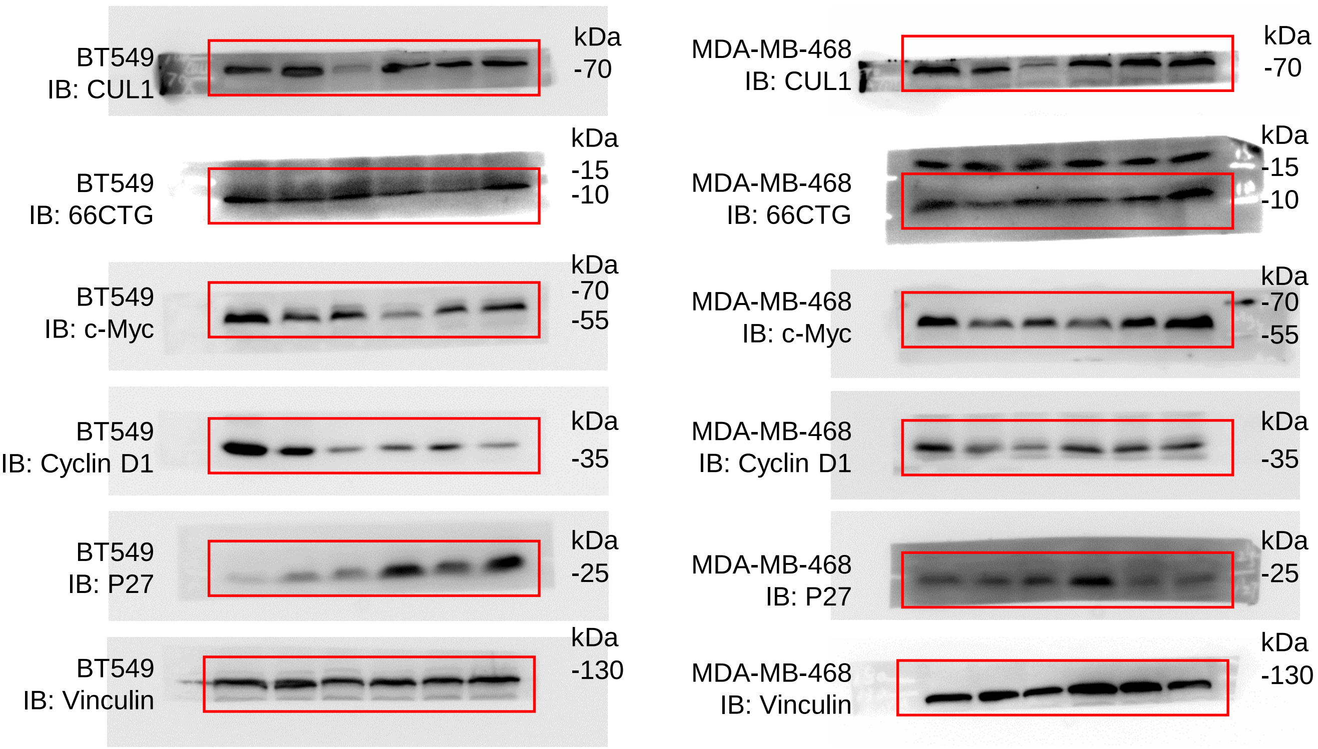
BT549
IB: CUL1
kDa
-70
MDA-MB-468
IB: CUL1
kDa
-70
BT549
IB: 66CTG
kDa
-15
-10
MDA-MB-468
IB: 66CTG
kDa
-15
-10
BT549
IB: c-Myc
kDa
-70
-55
MDA-MB-468
IB: c-Myc
kDa
-70
-55
BT549
IB: Cyclin D1
kDa
-35
MDA-MB-468
IB: Cyclin D1
kDa
-35
BT549
IB: P27
kDa
-25
MDA-MB-468
IB: P27
kDa
-25
BT549
IB: Vinculin
kDa
-130
MDA-MB-468
IB: Vinculin
kDa
-130

Fig. 5f

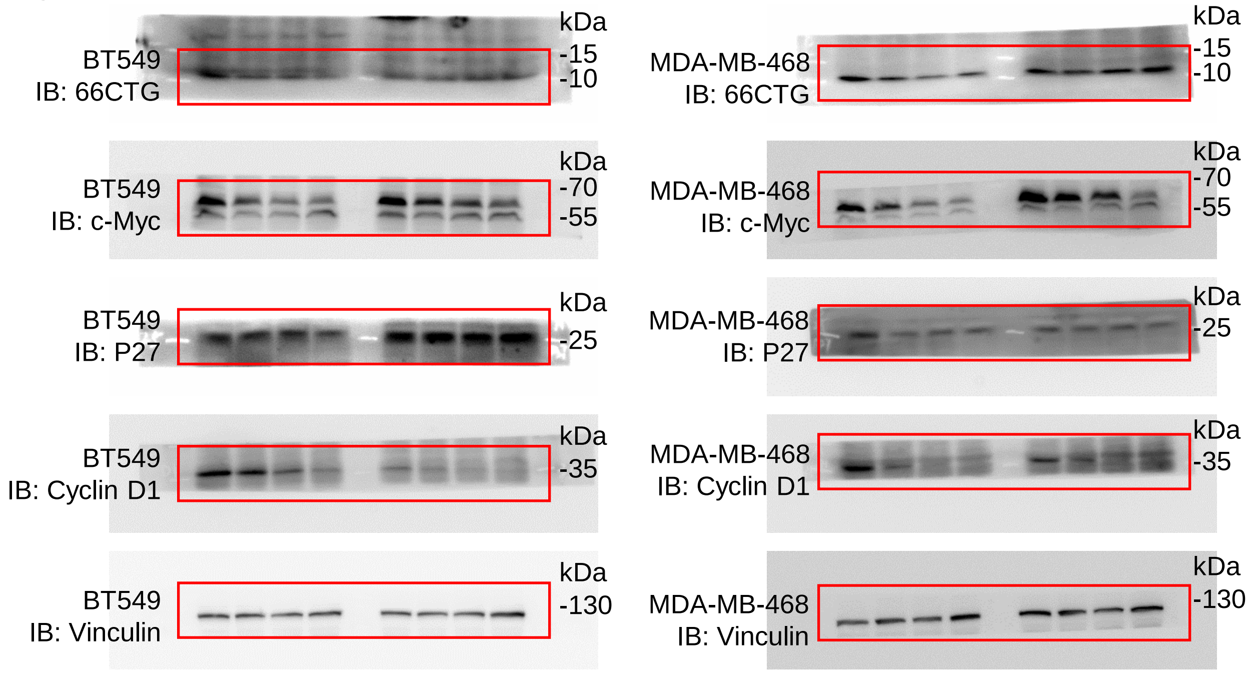

Supplementary Fig. S5c

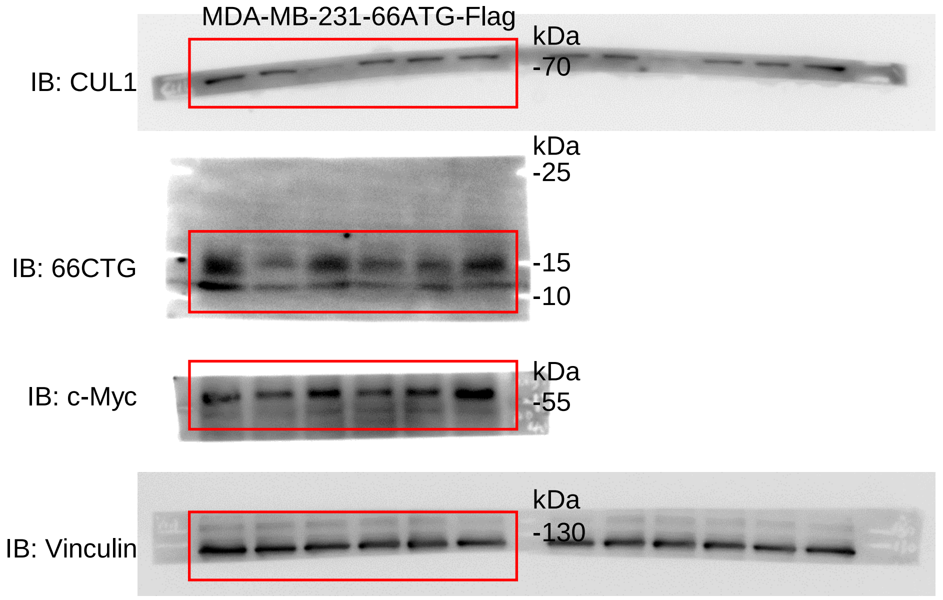

Supplementary Fig. S5e

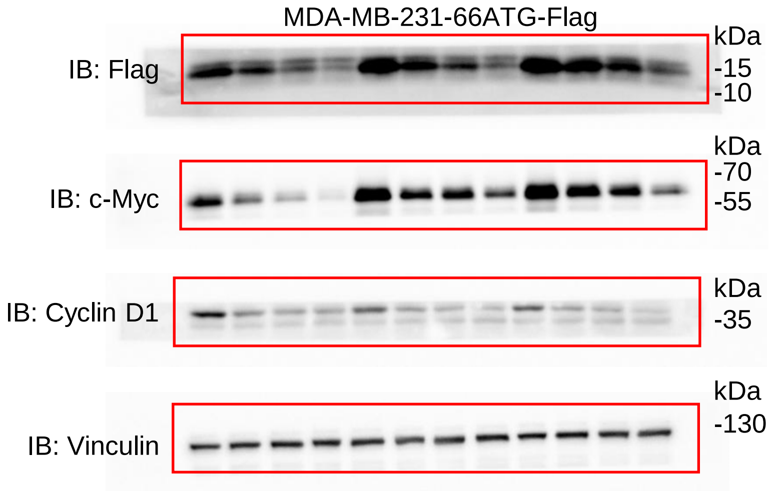

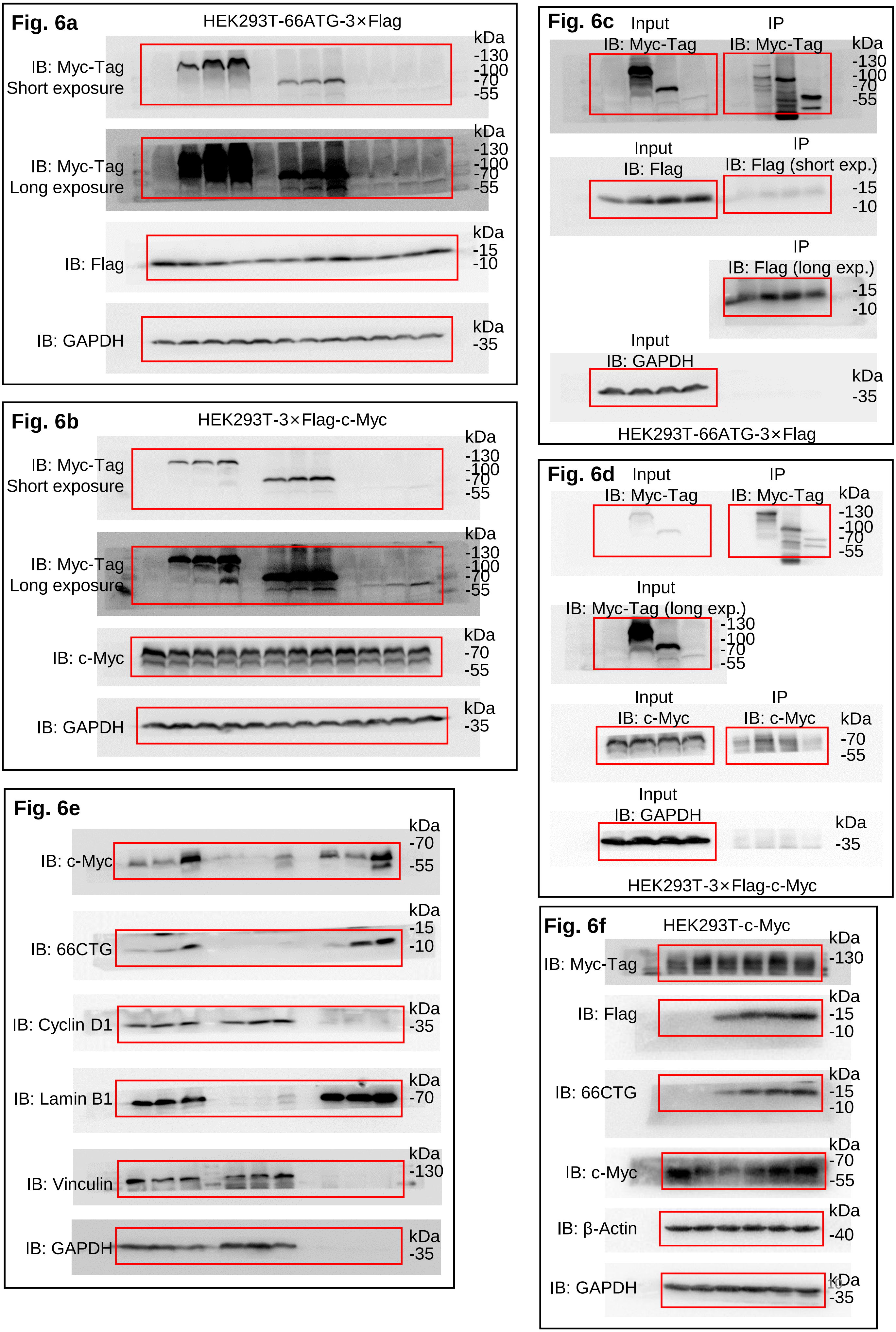
Fig. 6a
HEK293T-66ATG-3×Flag
IB: Myc-Tag Short exposure
IB: Myc-Tag Long exposure
IB: Flag
IB: GAPDH
kDa -130 -100 -70 -55
kDa -15 -10
kDa -35
Fig. 6b
HEK293T-3×Flag-c-Myc
IB: Myc-Tag Short exposure
IB: Myc-Tag Long exposure
IB: c-Myc
IB: GAPDH
kDa -130 -100 -70 -55
kDa -70 -55
kDa -35
Fig. 6c
Input IB: Myc-Tag
IP IB: Myc-Tag
Input IB: Flag
IP IB: Flag (short exp.)
IP IB: Flag (long exp.)
Input IB: GAPDH
kDa -130 -100 -70 -55
-15 -10
kDa -35
HEK293T-66ATG-3×Flag
Fig. 6d
Input IB: Myc-Tag
IP IB: Myc-Tag
Input IB: Myc-Tag (long exp.)
Input IB: c-Myc
IP IB: c-Myc
Input IB: GAPDH
kDa -130 -100 -70 -55
kDa -70 -55
kDa -35
HEK293T-3×Flag-c-Myc
Fig. 6e
IB: c-Myc
IB: 66CTG
IB: Cyclin D1
IB: Lamin B1
IB: Vinculin
IB: GAPDH
kDa -70 -55
kDa -15 -10
kDa -35
kDa -70
kDa -130
Fig. 6f
HEK293T-c-Myc
IB: Myc-Tag
IB: Flag
IB: 66CTG
IB: c-Myc
IB: β-Actin
IB: GAPDH
kDa -130
kDa -15 -10
kDa -70 -55
kDa -40
kDa -35

Fig. 6g

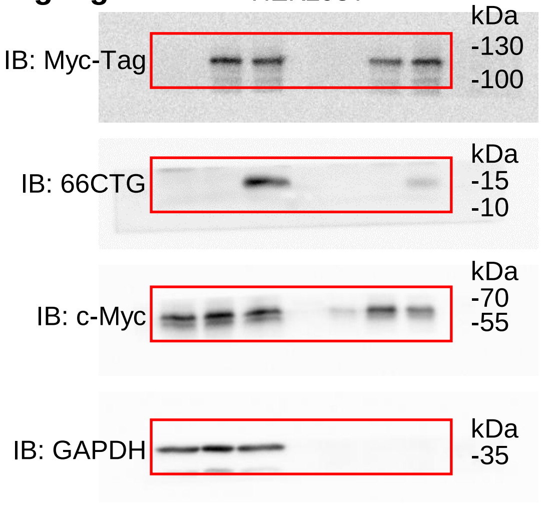

Fig. 6h

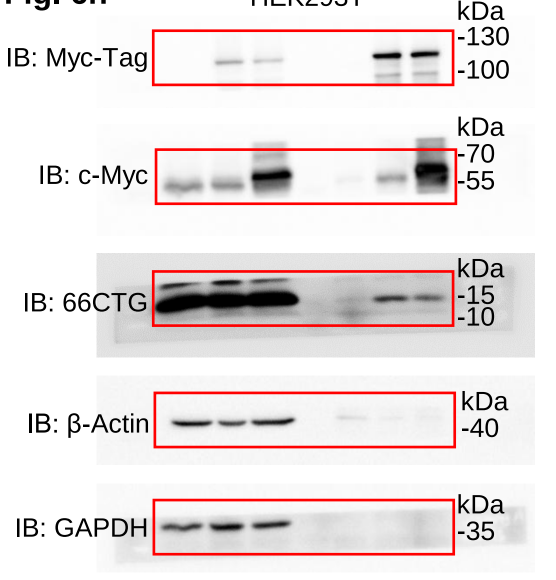

Fig. 6i

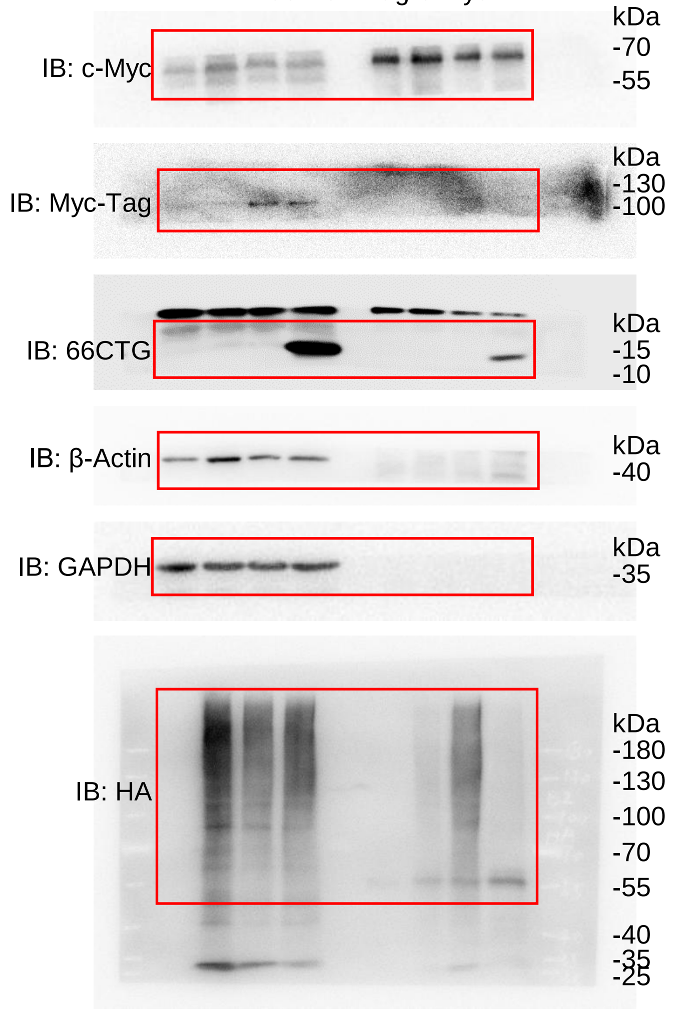

Suppplementary Fig. S6f

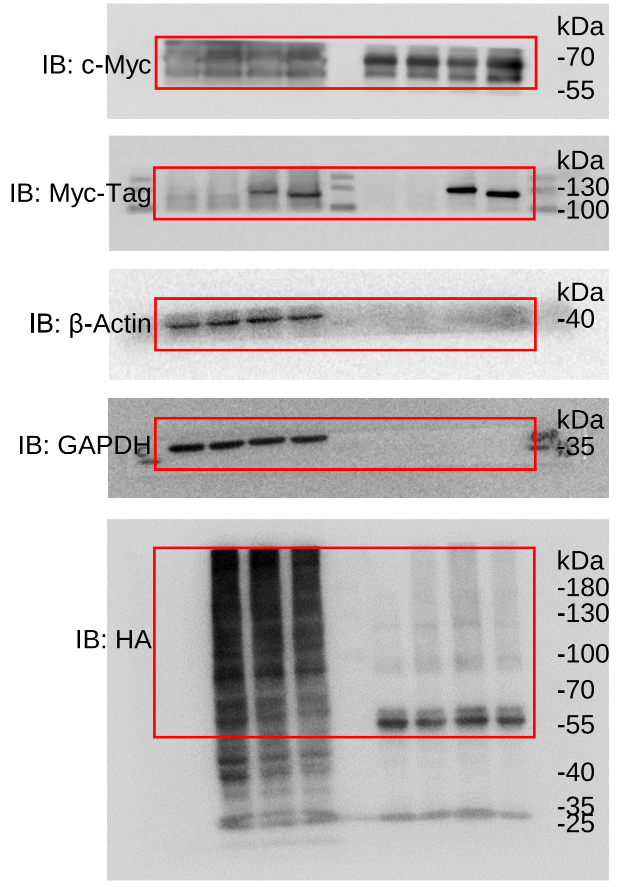

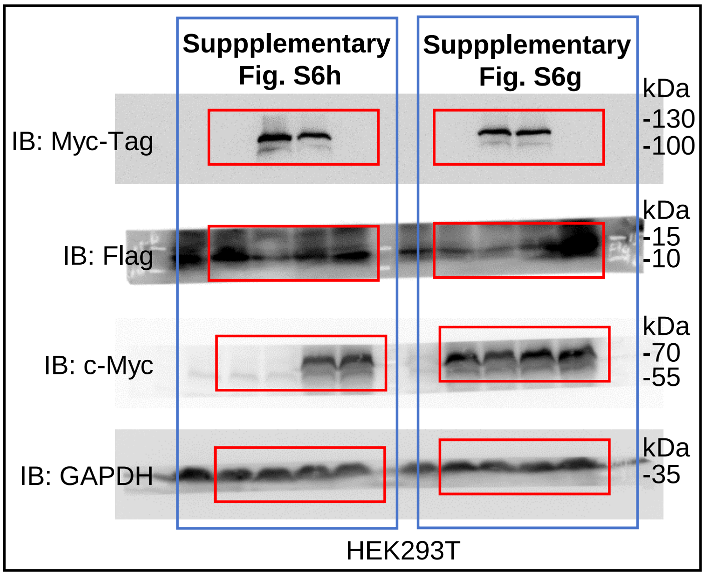
Suppplementary Fig. S6h
Suppplementary Fig. S6g
IB: Myc-Tag
IB: Flag
IB: c-Myc
IB: GAPDH
kDa
-130
-100
kDa
-15
-10
kDa
-70
-55
kDa
-35
HEK293T

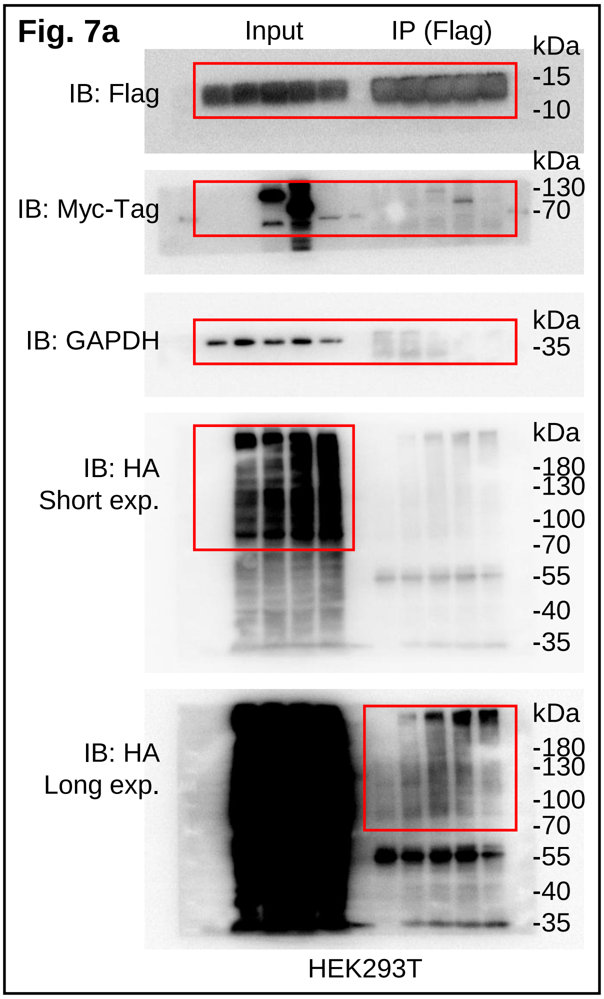
Fig. 7a
Input
IP (Flag)
IB: Flag
IB: Myc-Tag
IB: GAPDH
IB: HA
Short exp.
IB: HA
Long exp.
kDa
-15
-10
kDa
-130
-70
kDa
-35
kDa
-180
-130
-100
-70
-55
-40
-35
kDa
-180
-130
-100
-70
-55
-40
-35
HEK293T

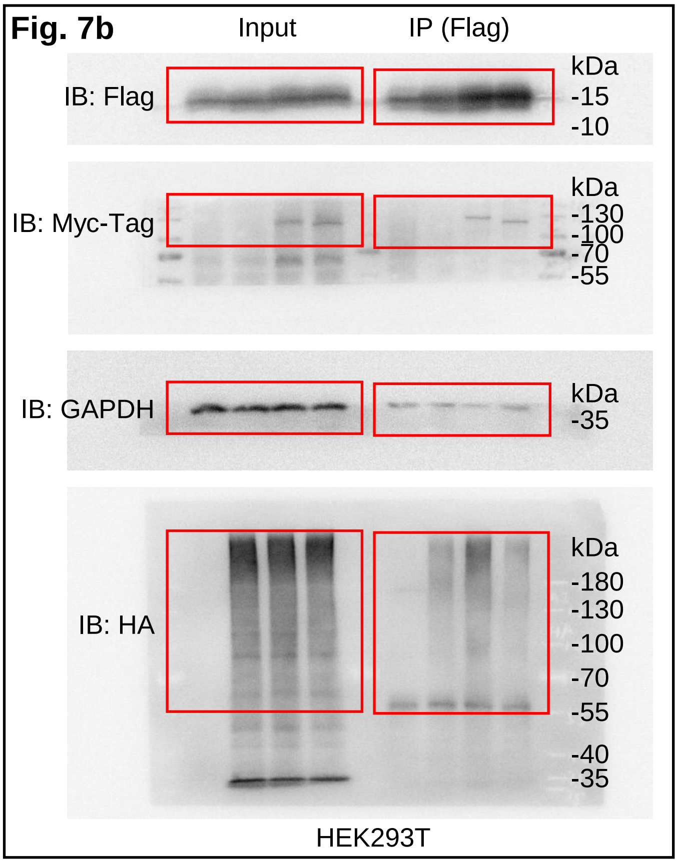
Fig. 7b
Input
IP (Flag)
IB: Flag
IB: Myc-Tag
IB: GAPDH
IB: HA
kDa
-15
-10
kDa
-130
-100
-70
-55
kDa
-35
kDa
-180
-130
-100
-70
-55
-40
-35
HEK293T

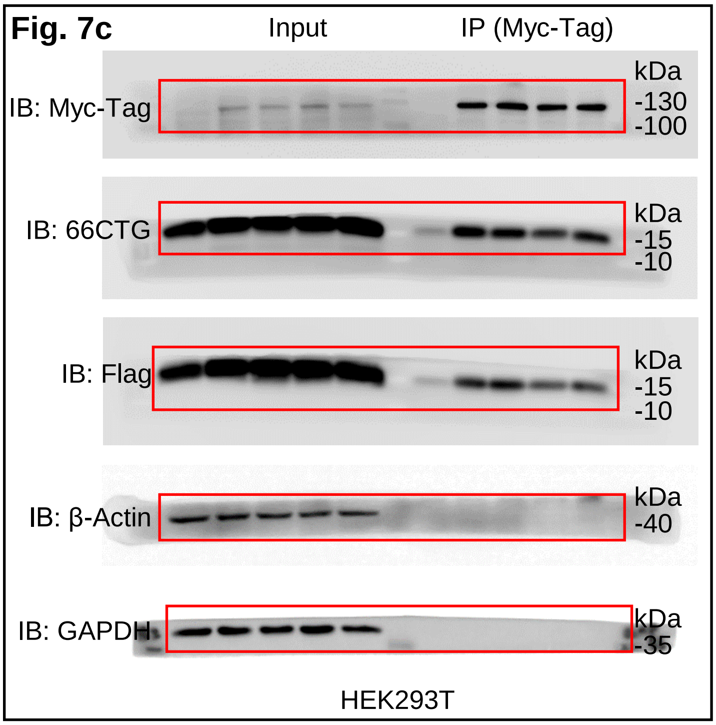
Fig. 7c
Input
IP (Myc-Tag)
IB: Myc-Tag
IB: 66CTG
IB: Flag
IB: β-Actin
IB: GAPDH
kDa
-130
-100
kDa
-15
-10
kDa
-15
-10
kDa
-40
kDa
-35
HEK293T

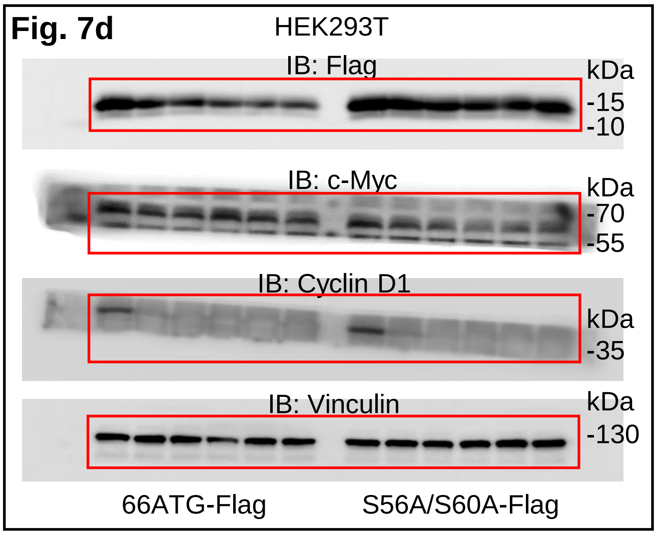
Fig. 7d
HEK293T
IB: Flag
IB: c-Myc
IB: Cyclin D1
IB: Vinculin
kDa
-15
-10
kDa
-70
-55
kDa
-35
kDa
-130
66ATG-Flag
S56A/S60A-Flag

Fig. 7f

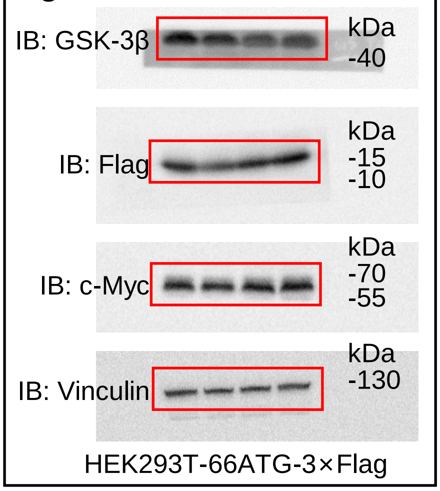

Fig. 7g

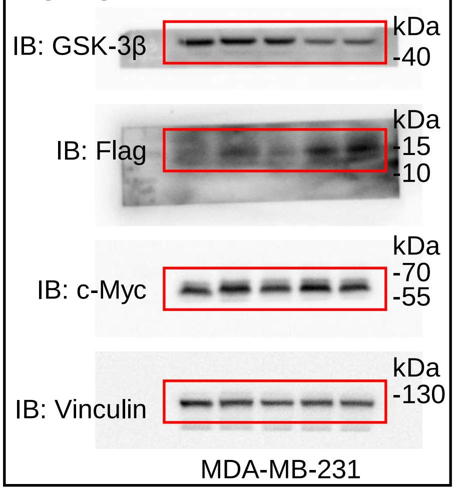

Suppplementary Fig. S7a

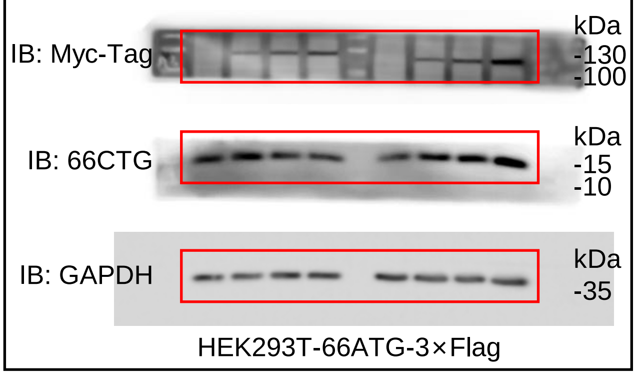

Suppplementary Fig. S7d

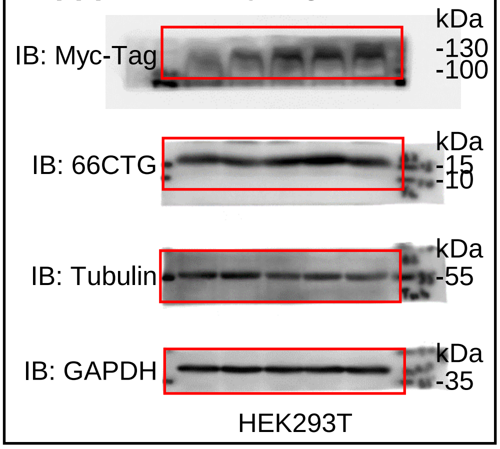

Suppplementary Fig. S7c

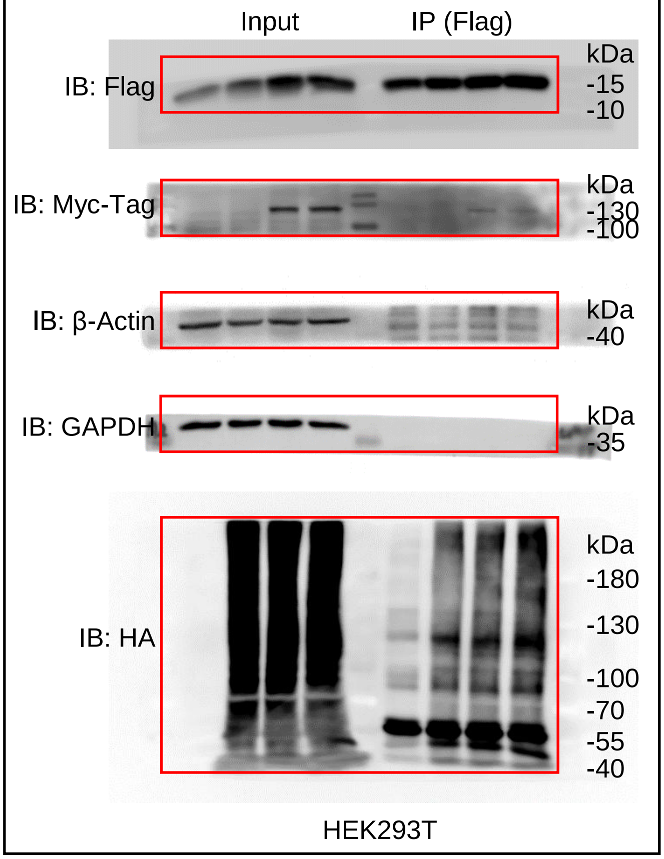

Suppplementary Fig. S7e

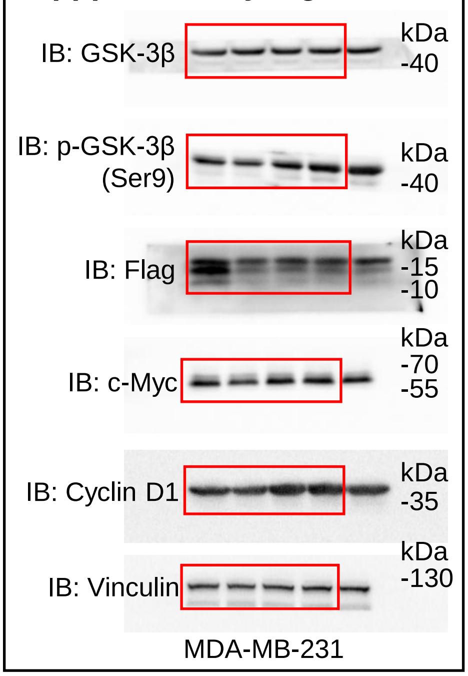

Supplement: Supplementary file 9 — Dataset 8 [file 41392_2025_2298_MOESM9_ESM.pdf]
