# Supplementary material for: A novel peptide 66CTG stabilizes Myc proto-oncogene protein to promote triple-negative breast cancer growth
Source: Signal Transduct Target Ther. 2025 Jul 9;10:217. doi: 10.1038/s41392-025-02298-5 (PMC12238259; doi:10.1038/s41392-025-02298-5)
Supplement: Supplementary file 10 — Dataset 9 [file 41392_2025_2298_MOESM10_ESM.pptx]

## Slide 1
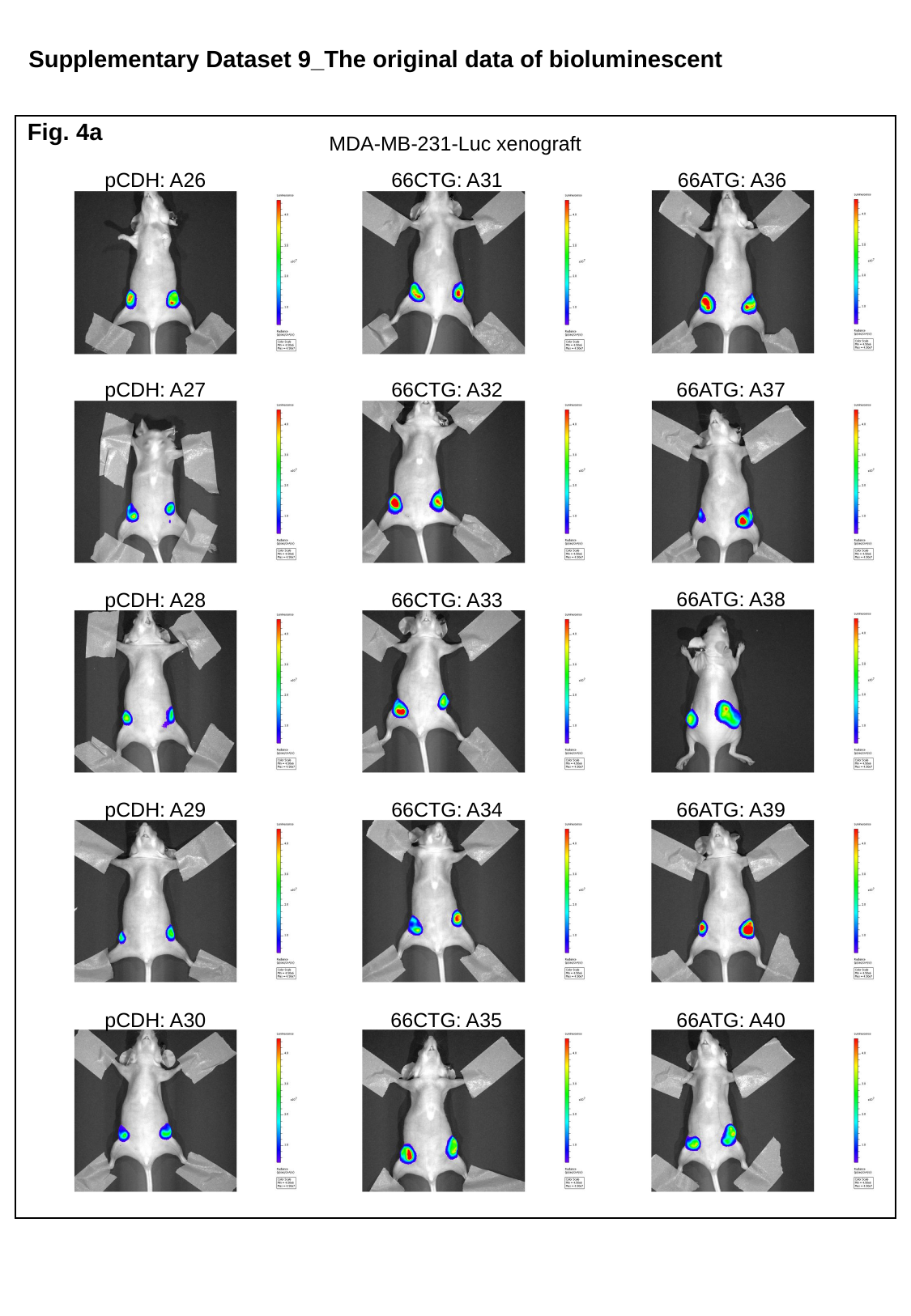

Supplementary Dataset 9_The original data of bioluminescent
Fig. 4a
MDA-MB-231-Luc xenograft
pCDH: A26
66CTG: A31
66ATG: A36
66ATG: A37
pCDH: A27
66CTG: A32
66ATG: A38
66CTG: A33
pCDH: A28
pCDH: A29
66ATG: A39
66CTG: A34
66ATG: A40
66CTG: A35
pCDH: A30
