# Supplementary material for: A novel peptide 66CTG stabilizes Myc proto-oncogene protein to promote triple-negative breast cancer growth
Source: Signal Transduct Target Ther. 2025 Jul 9;10:217. doi: 10.1038/s41392-025-02298-5 (PMC12238259; doi:10.1038/s41392-025-02298-5)
Supplement: Supplementary file 11 — Dataset 10 [file 41392_2025_2298_MOESM11_ESM.pptx]

## Slide 1
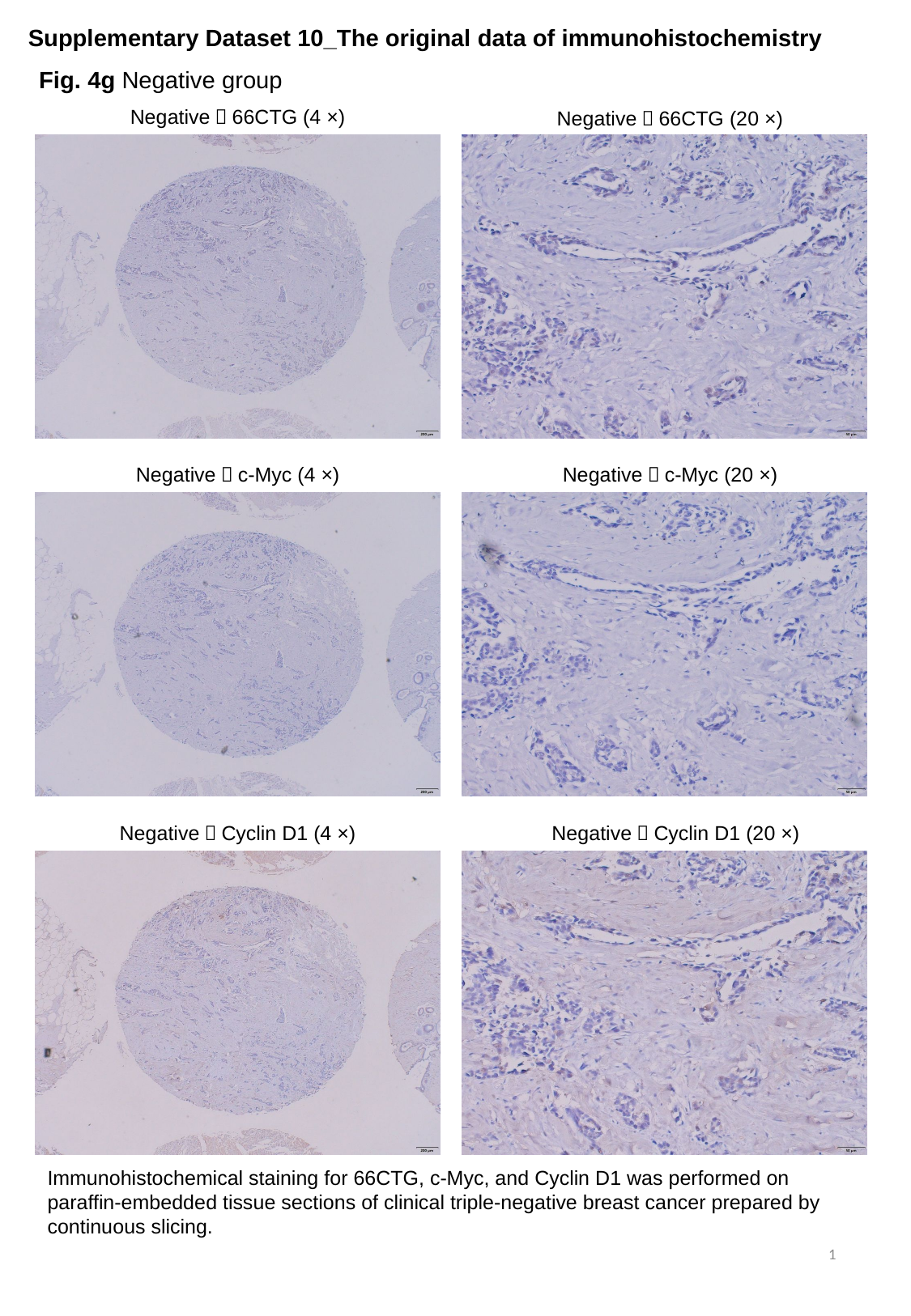

Supplementary Dataset 10_The original data of immunohistochemistry
Fig. 4g Negative group
Negative：66CTG (4 ×)
Negative：66CTG (20 ×)
Negative：c-Myc (20 ×)
Negative：c-Myc (4 ×)
Negative：Cyclin D1 (4 ×)
Negative：Cyclin D1 (20 ×)
Immunohistochemical staining for 66CTG, c-Myc, and Cyclin D1 was performed on paraffin-embedded tissue sections of clinical triple-negative breast cancer prepared by continuous slicing.
1

## Slide 2
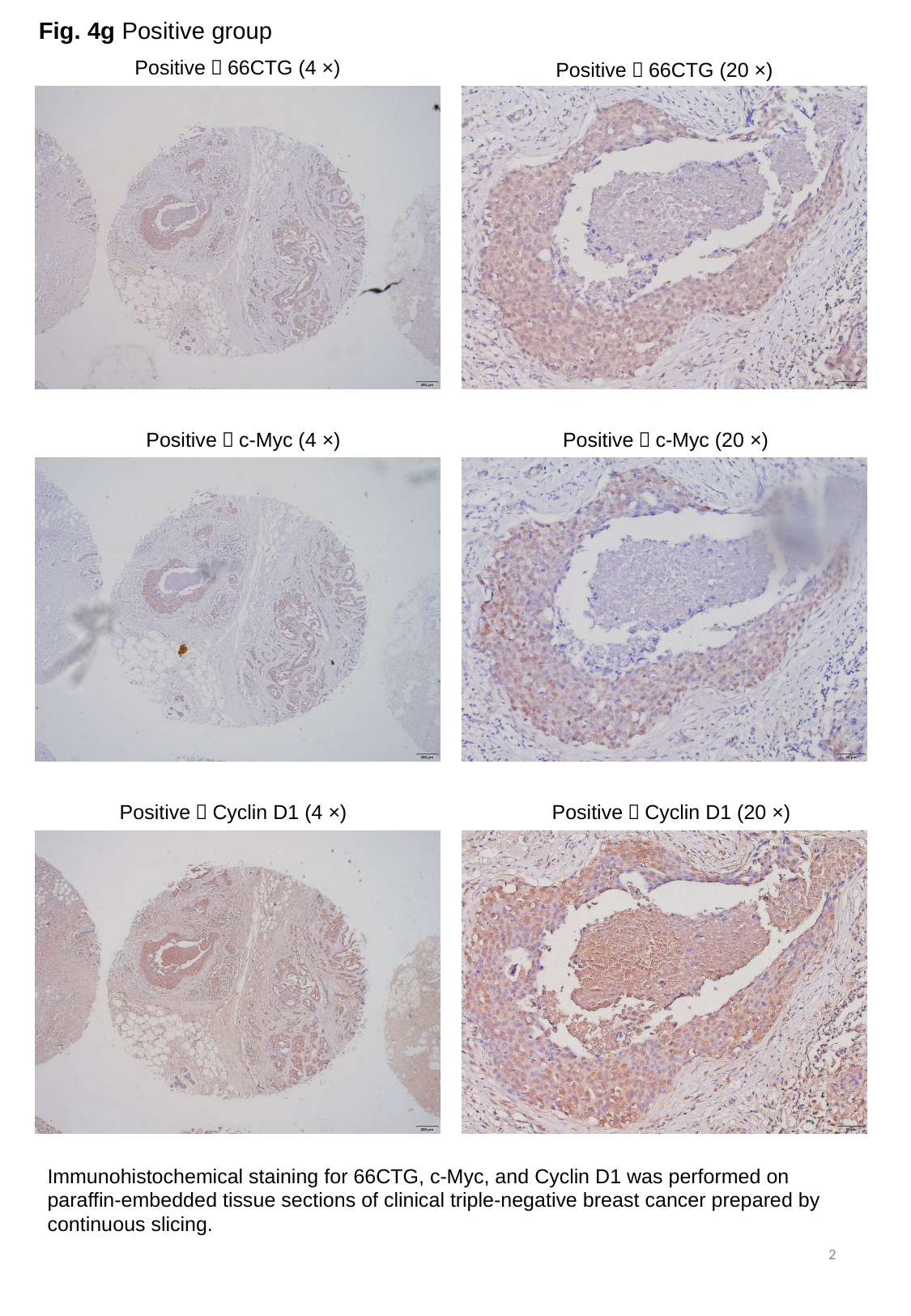

Fig. 4g Positive group
Positive：66CTG (4 ×)
Positive：66CTG (20 ×)
Positive：c-Myc (4 ×)
Positive：c-Myc (20 ×)
Positive：Cyclin D1 (4 ×)
Positive：Cyclin D1 (20 ×)
Immunohistochemical staining for 66CTG, c-Myc, and Cyclin D1 was performed on paraffin-embedded tissue sections of clinical triple-negative breast cancer prepared by continuous slicing.
2

## Slide 3
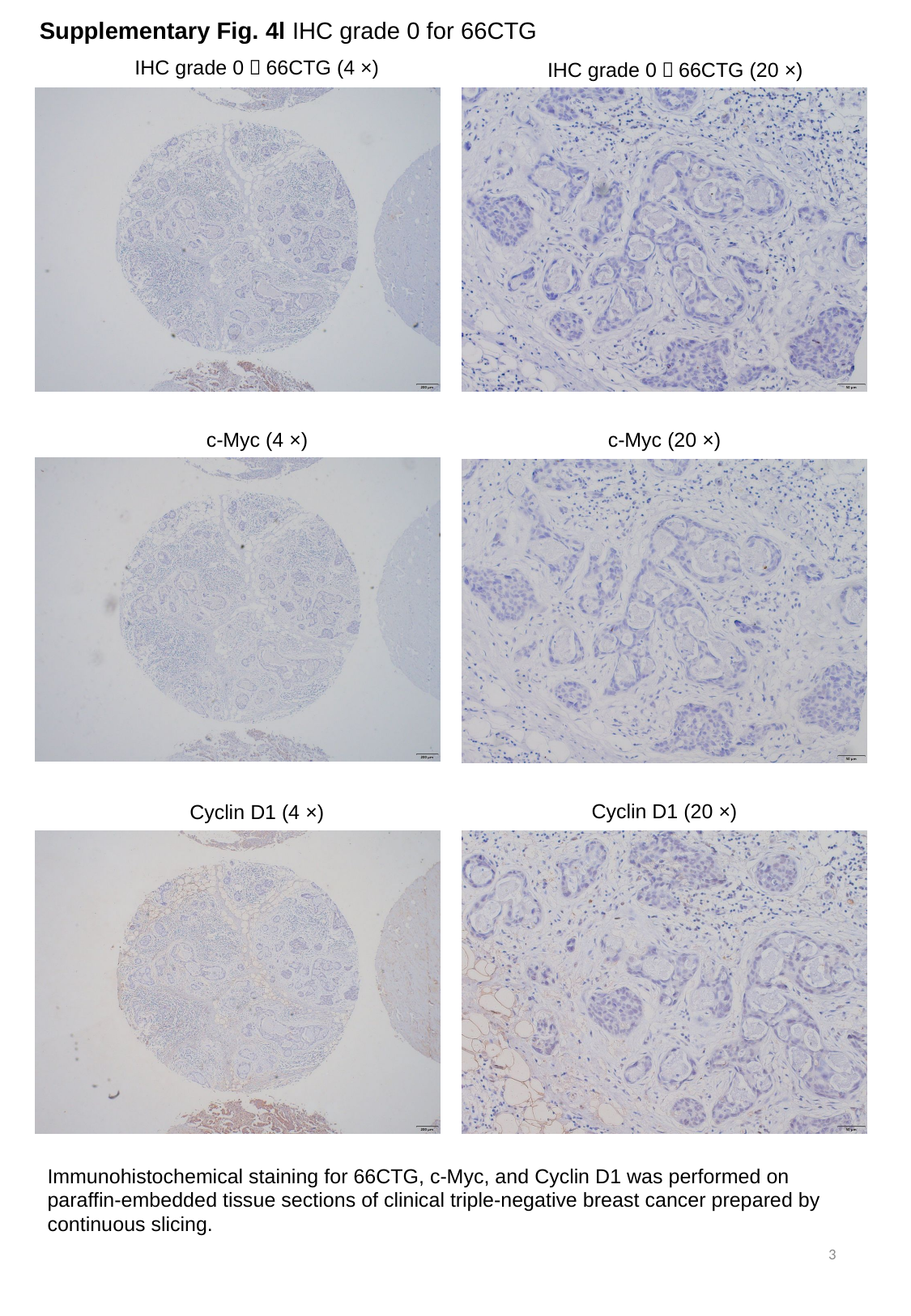

Supplementary Fig. 4l IHC grade 0 for 66CTG
IHC grade 0：66CTG (4 ×)
IHC grade 0：66CTG (20 ×)
c-Myc (4 ×)
c-Myc (20 ×)
Cyclin D1 (20 ×)
Cyclin D1 (4 ×)
Immunohistochemical staining for 66CTG, c-Myc, and Cyclin D1 was performed on paraffin-embedded tissue sections of clinical triple-negative breast cancer prepared by continuous slicing.
3

## Slide 4
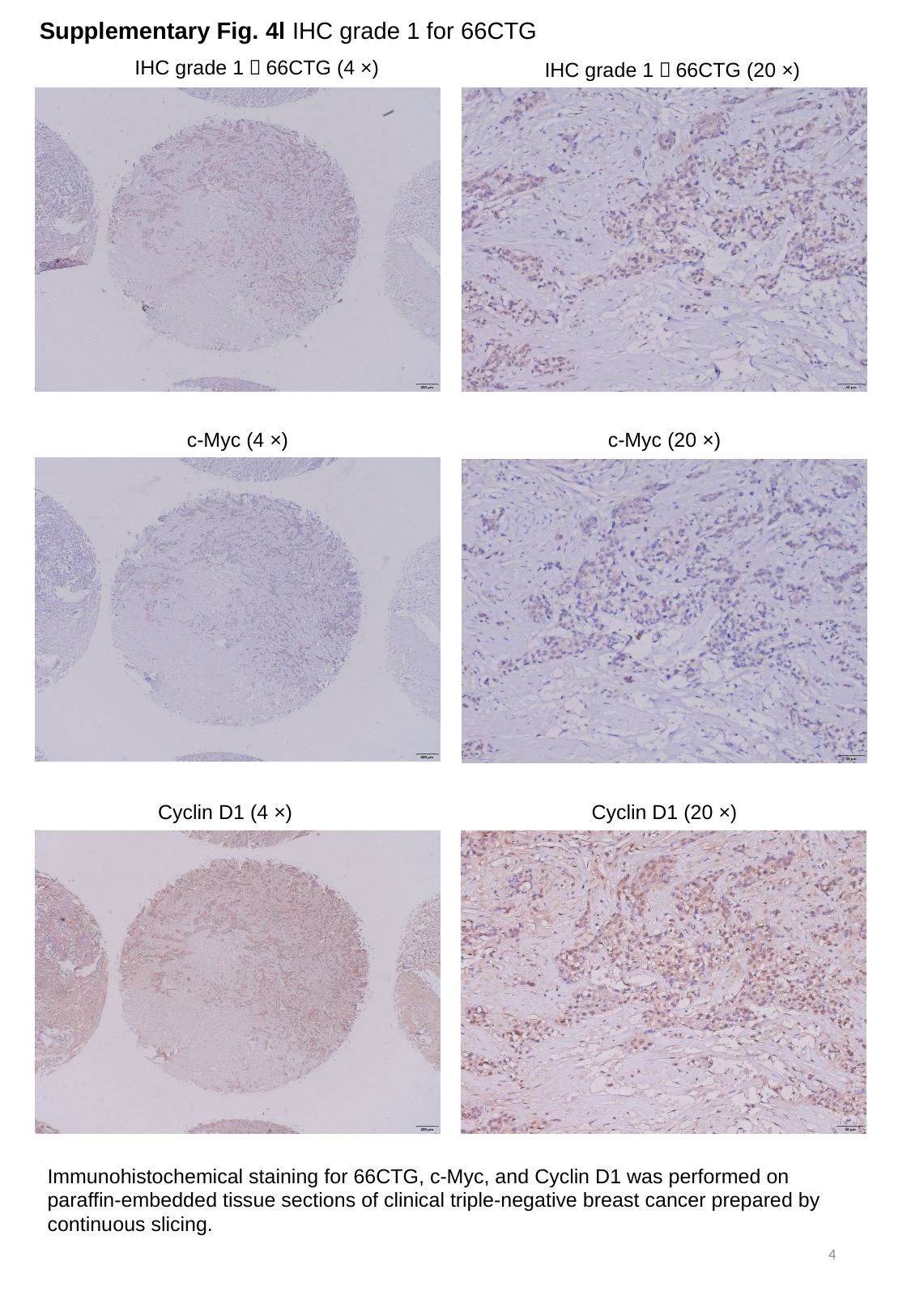

Supplementary Fig. 4l IHC grade 1 for 66CTG
IHC grade 1：66CTG (4 ×)
IHC grade 1：66CTG (20 ×)
c-Myc (20 ×)
c-Myc (4 ×)
Cyclin D1 (4 ×)
Cyclin D1 (20 ×)
Immunohistochemical staining for 66CTG, c-Myc, and Cyclin D1 was performed on paraffin-embedded tissue sections of clinical triple-negative breast cancer prepared by continuous slicing.
4

## Slide 5
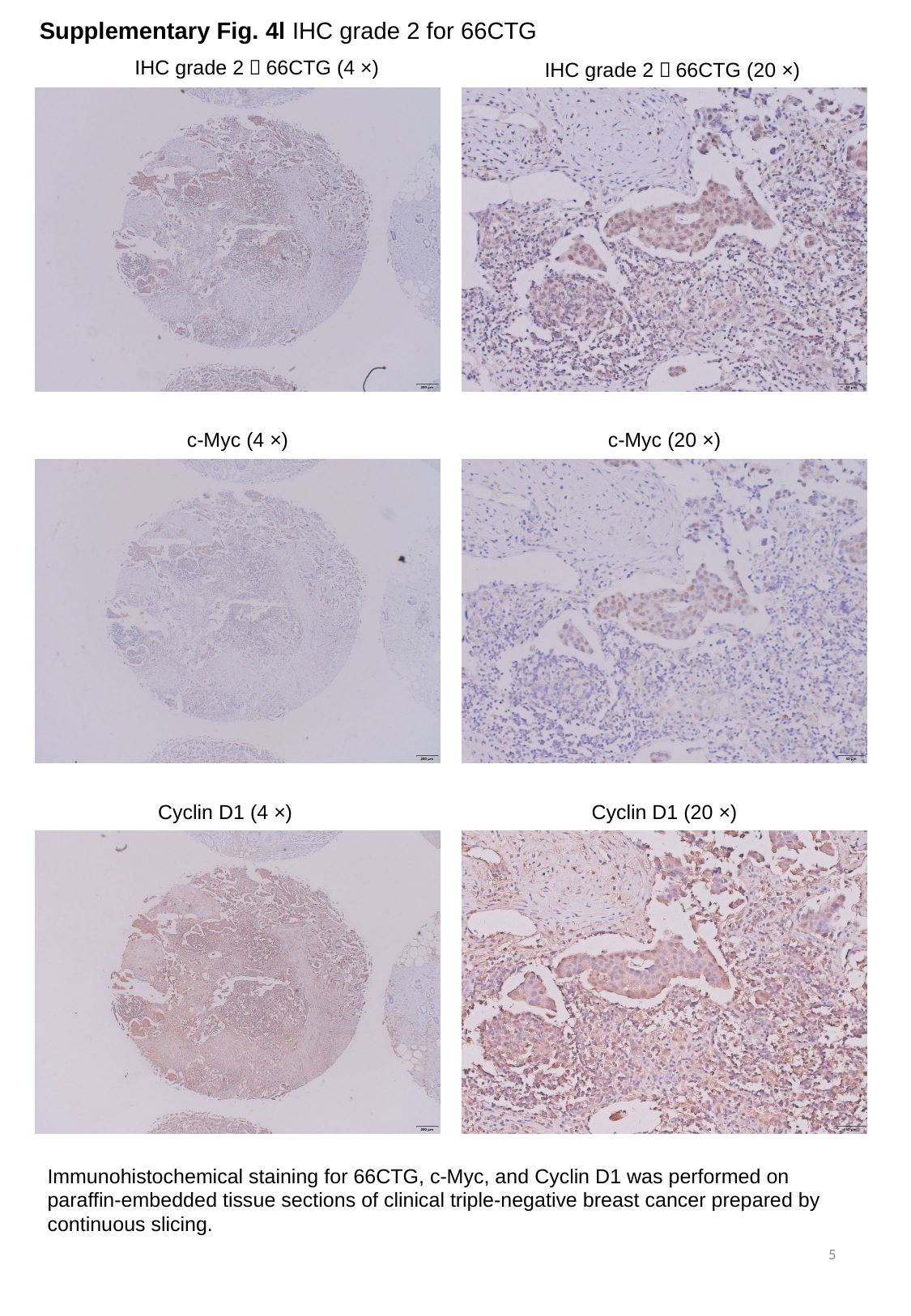

Supplementary Fig. 4l IHC grade 2 for 66CTG
IHC grade 2：66CTG (4 ×)
IHC grade 2：66CTG (20 ×)
c-Myc (20 ×)
c-Myc (4 ×)
Cyclin D1 (4 ×)
Cyclin D1 (20 ×)
Immunohistochemical staining for 66CTG, c-Myc, and Cyclin D1 was performed on paraffin-embedded tissue sections of clinical triple-negative breast cancer prepared by continuous slicing.
5

## Slide 6
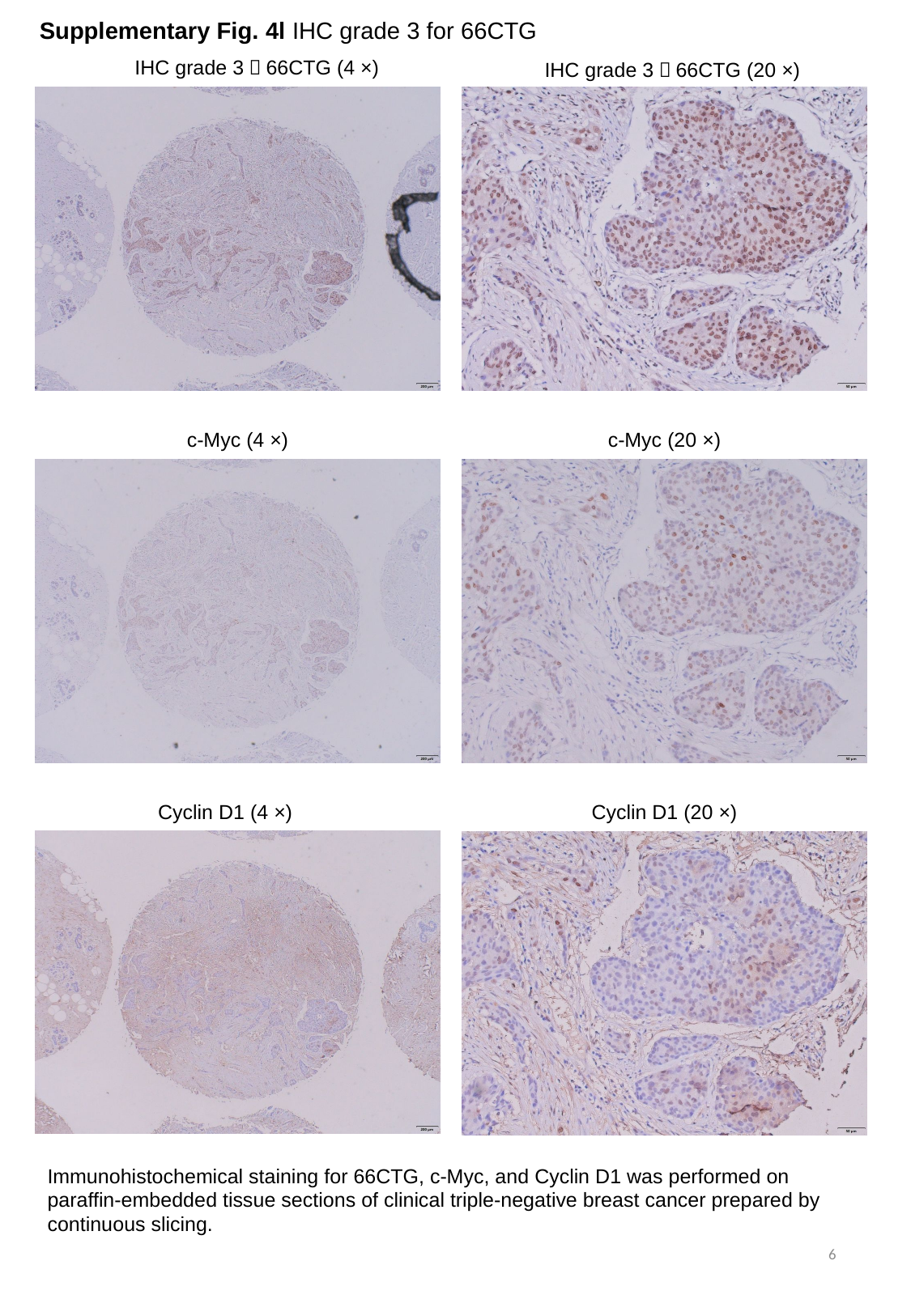

Supplementary Fig. 4l IHC grade 3 for 66CTG
IHC grade 3：66CTG (4 ×)
IHC grade 3：66CTG (20 ×)
c-Myc (20 ×)
c-Myc (4 ×)
Cyclin D1 (4 ×)
Cyclin D1 (20 ×)
Immunohistochemical staining for 66CTG, c-Myc, and Cyclin D1 was performed on paraffin-embedded tissue sections of clinical triple-negative breast cancer prepared by continuous slicing.
6
